# The efficacy and effectiveness of enterovirus A71 vaccines against hand, foot, and mouth disease: A systematic review and meta-analysis

**DOI:** 10.1371/journal.pone.0323782

**Published:** 2025-05-22

**Authors:** Xuemei Yan, Yuanhua Liu, Fengkun Chen, Zhaorui Chang, Zhijie Zhang, Margarita Pons-Salort, Nicholas C. Grassly

**Affiliations:** 1 MRC Centre for Global Infectious Disease Analysis, School of Public Health, Imperial College London, London, United Kingdom; 2 Department of Epidemiology and Health Statistics, School of Public Health, Fudan University, Shanghai, China; 3 Division of Infectious Disease, Key Laboratory of Surveillance and Early-warning on Infectious Disease, Chinese Center for Disease Control and Prevention, Beijing, China; National Taiwan Ocean University, TAIWAN

## Abstract

Enterovirus A71 (EV-A71) is a major cause of severe hand, foot, and mouth disease (HFMD) among children in the Asia-Pacific region. Here we review randomised clinical trial data and post-licensure effectiveness studies of inactivated EV-A71 vaccines following their development and licensure in China since late 2015.

We searched PubMed, Web of Science Core Collection, Elsevier ScienceDirect, China National Knowledge Infrastructure (CNKI), and Wanfang Data on 20 May 2024 with no date restriction in English and Mandarin, using key terms including “EV-A71”, “enterovirus 71”, “hand, foot, and mouth disease”, “HFMD”, “vaccine”, “efficacy”, “effectiveness”, “protection” and “impact”. Phase III randomized controlled trials (RCTs) reporting vaccine efficacy and observational studies on effectiveness were eligible for inclusion. We excluded studies that evaluated non-laboratory confirmed HFMD associated outcomes, abstracts, reviews, comments, animal studies, cross-sectional studies, and modelling studies. Summary measures of vaccine efficacy and effectiveness were based on random-effects models.

After screening, 14 articles were eligible for inclusion, including 6 reporting 4 different phase III RCTs. Estimated efficacy of 2 doses of EV-A71 vaccine against EV-A71 associated HFMD 1 year after vaccination ranged from 90.0% to 97.9%, with an overall estimate for all products of 95.6% (95% Confidence Interval: 92.1, 97.5). Efficacy remained high at 26 months post-vaccination, ranging from 94.7% to 94.8%. The estimated overall effectiveness of 2-dose vaccination against any EV-A71-HFMD across five test-negative case-control studies was 84.3% (95% CI: 75.2, 90.0) among children aged 0–12 years. Effectiveness was higher in older children compared to younger children (85.3% (72.9, 92.1) vs. 79.8% (61.2, 89.5)) and higher against severe compared with non-severe EV-A71-HFMD (90.0% (80.0, 95.0) vs. 76.5% (50.0, 89.0)). The effectiveness declined in more recent studies with longer follow-up.

Inactivated EV-A71 vaccines offer a high level of protection against EV-A71 HFMD. Longer term studies are needed to evaluate the persistence of protection beyond 2 years post-vaccination.

## Introduction

Over 100 serotypes and 4 species of enterovirus have been reported worldwide and serve as causative agents for a broad spectrum of illnesses, ranging from common colds to more severe conditions such as poliomyelitis and aseptic meningitis [1]. Notably, hand, foot, and mouth disease (HFMD) is caused by several well-known enterovirus serotypes from species A, with enterovirus A71 (EV-A71), coxsackievirus A16 (CVA16), CVA6 and CVA10 being prominent examples. Children younger than 5 years are especially prone to HFMD, and most patients show self-limiting illness typically including fever, and a rash on hands, feet, mouth, and buttocks [2]. While the majority of HFMD cases follow a benign course, some patients can rapidly develop neurological and systemic complications, which pose a serious risk and can potentially lead to fatal outcomes. Notably, cases associated with EV-A71 infections are more likely to be linked with severe manifestations [3]. By the late 1990’s, EV-A71 became endemic in Asia-Pacific region and has since caused major outbreaks, placing a substantial disease burden [4].

No specific antiviral against HFMD and no WHO-approved vaccines against any HFMD-related enterovirus are currently available. A range of candidate monovalent and multivalent vaccines against HFMD-related enterovirus serotypes are in various stages of development, with some of them having already entered into clinical development [5]. Particularly, three monovalent C4 strain-based inactivated EV-A71 vaccines reached the licensing stage and were approved by the National Medical Products Administration (NMPA) in late 2015 and 2016 in mainland China [6], and two B4 strain-based inactivated EV-A71 vaccines were approved in Taiwan, China in 2023 [7]. Those vaccines were developed by Sinovac Biotech, Beijing Vigoo, the Chinese Academy of Medical Science (CAMS), Medigen Vaccine Biologic’s Hsinchu (MVC), and ENIMMUNE respectively. Since the C4 strain-based vaccines’ approval and introduction to the private market in 2016, the cumulative vaccination coverage of the EV-A71 vaccines in mainland China has slowly increased and has been estimated at 25% in 2021 among 6-month to 5-year-old children [8]. Coverage varies substantially by province. No countries other than China are currently using these vaccines.

Understanding the effectiveness of these EV-A71 vaccines post-licensure is important to establish evidence of vaccine programme impact and refine vaccination strategies, including the need for booster doses and development of multivalent HFMD vaccines. In this study, we conducted a systematic review and meta-analysis of studies reporting on the efficacy in clinical trials and the effectiveness in the real world of EV-A71 vaccines, to provide a comprehensive overview of EV-A71 vaccine protection.

## Methods

### Search strategy and selection criteria

We conducted a search for published articles on efficacy and effectiveness of EV-A71 vaccines across multiple databases, with the search timeframe extending from the beginning up to May 20, 2024. Databases included PubMed, Web of Science Core Collection, Elsevier ScienceDirect, as well as two widely used Chinese databases: China National Knowledge Infrastructure (CNKI) and Wanfang Data. The inclusion of Chinese databases was crucial as vaccines against EV-A71 are exclusively approved for marketing in China, and numerous articles on EV-A71 vaccine effectiveness have been published in Chinese journals. Our search strategy was tailored to each database, employing key search terms such as “EV-A71”, “enterovirus 71”, “hand, foot, and mouth disease”, “HFMD”, “vaccine”, “efficacy”, “effectiveness”, “protection” and “impact”. The full search query is available in the **Supplementary Information** S1 Table.

To ensure the quality of retrieved Chinese articles, we refined our search criteria within the Chinese databases, selecting academic articles exclusively from core journals, including Peking University core journals, Chinese Social Sciences Citation Index (CSSCI) journals, and Chinese Science Citation Database (CSCD) journals. For PubMed and Web of Science Core Collection, we restrained language to English to avoid duplicates from Chinese articles in CNKI and Wanfang Data. Phase III randomized controlled trials (RCTs) investigating EV-A71 vaccine efficacy and observational studies examining EV-A71 vaccine effectiveness were deemed eligible for inclusion. Studies encompassing individuals with/without the clinical outcome under investigation, and with/without EV-A71 vaccination, were considered. Exclusion criteria included studies that evaluated non-laboratory confirmed HFMD associated outcomes, abstracts, reviews, comments, study protocols, and author responses. Studies involving non-human animal samples, cross-sectional designs, modelling studies, and impact assessments were also excluded. The systematic review followed the PRISMA 2020 guidelines (S2 Table).

### Data analysis

Two independent reviewers searched the included databases (Yan XM and Liu YH). All citations were managed in Covidence and Endnote 20 to remove duplicates and perform the screening. Evaluation of title, abstract and full text were screened separately by (Yan XM, Liu YH, and Chen FK) and disagreements were resolved by discussion. Studies were considered eligible for full-text screening if they reported effectiveness, efficacy, protection, or impact of the EV-A71 vaccines. Essential variables of each article included in the systematic review were extracted by Yan XM (and checked by Liu YH and Chen FK), including literature information, study design, characteristics of study participants, exposure history and outcomes, diagnostic methods, number of subjects in each group, statistical analysis, and estimates of vaccine effectiveness/efficacy. To assess study quality, we used the Risk of Bias in Non-randomized Studies – of Interventions (ROBINS-I) assessment tool for observational studies and Cochrane Risk of Bias (RoB) assessment tool for RCT studies.

The primary endpoint was efficacy and effectiveness of EV-A71 vaccines against EV-A71 related HFMD, and secondary endpoints included efficacy and effectiveness against EV-A71 related mild and severe HFMD, hospitalization, herpangina, and all EV-A71 relate diseases. We provide a detailed description for each of the eligible studies. Forest plots were generated to present the estimates of vaccine efficacy and effectiveness across the selected studies, and random-effects models were used to estimate the overall effect accounting for heterogeneity across studies. As a sensitivity analysis, we also estimated the overall effect using a fixed-effects model. Funnel plots were used to assess publication bias. The effect size was reported as vaccine efficacy or effectiveness based on the relative risk (RR) or odds ratio (OR) respectively and associated 95% confidence interval (CI) level.

All analyses and funnel plots were conducted with meta package, and forest plots were visualized with forestploter package in R 4.3.1 [9,10].

## Results

**Fig 1:**
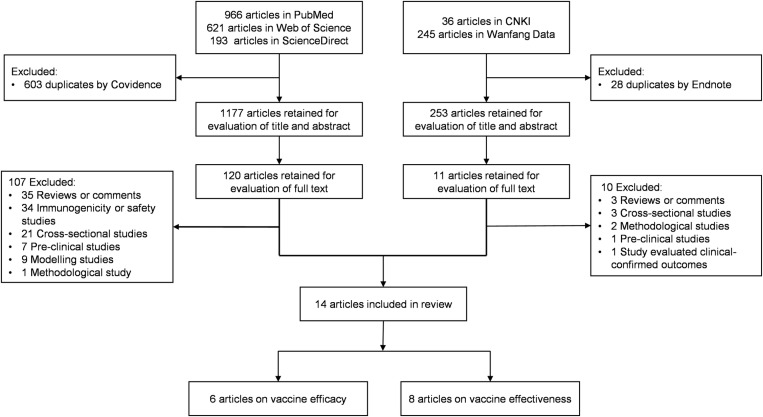
Selection flowchart of studies reporting on EV-A71 vaccines efficacy and effectiveness.

On May 20, 2024, the initial search returned 2,061 articles from the five databases, of which 1,177 articles in English and 253 articles in Chinese were retained for further screening, following the removal of duplicates. Upon evaluation of titles and abstracts, 131 articles met the criteria for further assessment (S3 Table). After full-text review of those, 14 articles were deemed eligible and included for analysis in the review (S4 Table), of which 6 reported on EV-A71 vaccine efficacy measured in RCTs and 8 on EV-A71 vaccine effectiveness estimated in post-licensure studies (Fig 1). The overall quality of the studies included in this review was deemed to be high. All the observational studies were at low or moderate risk of bias for all assessed domains, while randomized controlled trials (RCTs) were determined to be at low risk of bias. Details can be found in the **Supplementary Information** S5 Table and S1 Fig.

### Vaccine efficacy

**Fig 2:**
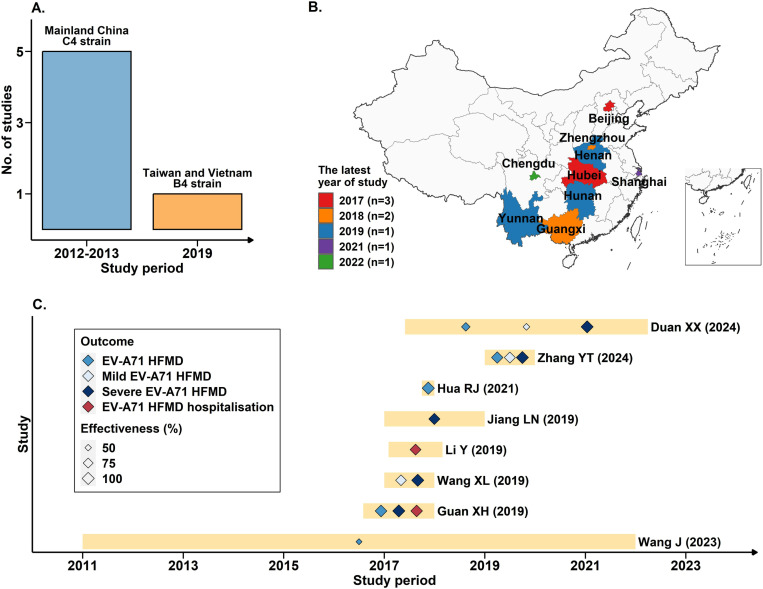
Spatial and temporal characteristics of eligible studies included in the meta-analysis. (A) Study period and number of publications reporting on EV-A71 vaccine efficacy in clinical trials. (B) Location of study sites, coloured according to the last year of the study. n indicates the number of studies that end each year. Note that two studies had sites in Shanghai; one ended in 2017 and one in 2021. (C) Study period (yellow bands) and main results of studies on EV-A71 vaccine effectiveness in real world settings.

6 studies of efficacy evaluation of EV-A71 inactivated vaccines were included, reporting results from 4 large phase III RCTs [11–16]. 5 of those studies were from 3 RCTs conducted in mainland China between 2012 and 2013, and all focused on C4 strain-based EV-A71 vaccines developed by Sinovac, Vigoo and CAMS. The other study was conducted in 2019 and targeted a B4 strain-based EV-A71 vaccine developed by MVC (Fig 2A). The immunization schedule of these 2 vaccine groups were different in their corresponding RCTs. For the C4 strain-based vaccines RCTs, two doses were administered with a 28-day interval between doses to all participants aged 6–35 and 6–71 months old, respectively. For the B4 strain-based vaccine RCT, two doses were administered with a 56-day interval to participants aged over 23 months old, with an additional third dose administered one year after the initial dose for those aged between 2–23 months old. The maximum follow-up period across all studies was 26 months post complete vaccination, while the minimum period was 11 months. All outcomes were rigorously laboratory-confirmed using PCR or viral isolation techniques. Detailed information on the eligible studies is shown in Table 1.

**Table 1:** Characteristics of selected studies reporting on the efficacy of EV-A71 inactivated vaccines.

Study	Location	Study time	Participant age	Sample	Vaccine, sub-genotype	Manufacture	Vaccination schedule (Dose, interval)	Case confirmation	Outcome	Follow up period
Zhu FC (2014) [11]	Jiangsu, China	2012.1-2013.3	6–35 months	10,077	Vero cell based, C4	Sinovac	2 doses, 28 days	PCR and viral isolation	EV-A71-HFMD, EV-A71-herpangina	12 months
Li JX (2016) [12]	Jiangsu, China	2012.1-2013.3	6–35 months	10,077	Vero cell based, C4	Sinovac	2 doses, 28 days	PCR and viral isolation	EV-A71-HFMD	26 months
Zhu FC (2013) [13]	Jiangsu and Beijing, China	2012-2013	6–35 months	10,245	Vero cell based, C4	Vigoo	2 doses, 28 days	PCR	EV-A71-HFMD	12 months
Wei MW (2017) [14]	Jiangsu and Beijing, China	2012-2013	6–35 months	10,245	Vero cell based, C4	Vigoo	2 doses, 28 days	PCR	EV-A71-HFMD	26 months
Li RC (2014) [15]	Guangxi, China	2012.3-2013.2	6–71 months	12,000	Human diploid cell based, C4	CAMS	2 doses, 28 days	PCR	EV-A71-HFMD	11 months
Nguyen TT (2022) [16]	Taiwan, Vietnam	2019.4-2019.12	2–71 months	3,049	Whole-virus, B4	MVC	2 doses, 56 days; Children aged 2–23 months received a third booster dose on day 366	CODEHOP assays, PCR or viral isolation	EV-A71-disease	2 years

**Fig 3:**
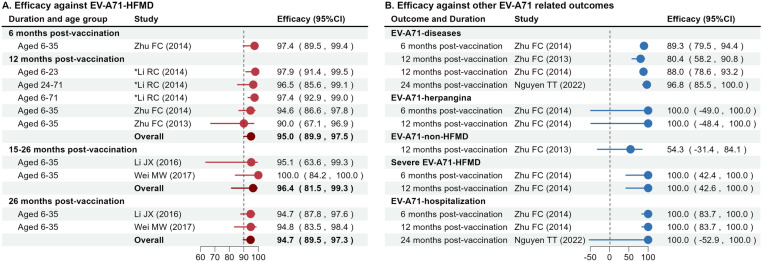
Estimates of vaccine efficacy of a 2-dose vaccination schedule against EV-A71 infection-related outcomes. (A) Efficacy against EV-A71 related HFMD. The overall efficacy was estimated by random-effects models, and only the main results from the selected studies were used to estimate the overall efficacy in each post-vaccination group, excluding age-group specific results. * The follow up period was 11 months post-vaccination. (B) Efficacy against EV-A71 related outcomes other than HFMD. EV-A71-non-HFMD referred to upper respiratory tract infection and diarrhoea.

The efficacy of EV-A71 vaccines against HFMD was reported in 4 time periods following the completion of the two-dose vaccination schedule. Zhu et al. study [11] reported an efficacy of 97.4% (95% CI: 89.5, 99.4) among children aged 6–35 months within 6 months post-vaccination. One year after vaccination, estimates of efficacy against HFMD ranged from 90.0% to 97.9% [11,13,15], with an overall efficacy estimated at 95.0% (95% CI: 89.9, 97.5) across children aged 6–71 months. The reported heterogeneity determined by I² statistic was 31.9% (95% CI: 0, 92.9) for these studies (S2 Fig). Two studies [12,14] reported efficacy after an extended follow up period of 26 months. The efficacy at 26 months post-vaccination was 94.7% (95% CI: 87.8, 97.6) and 94.8% (95% CI: 83.5, 98.4), while the immediate efficacy within 15–26 months was 95.1% (95% CI: 63.6, 99.3) and 100% (95% CI: 84.2, 100). The overall efficacy within 26 months and within 1526 months, estimated with the random-effects models was 94.7 (95% CI: 89.5, 97.3) and 96.4% (95% CI: 81.5, 99.3), respectively (Fig 3A). No statistical heterogeneity and publication bias were estimated, probably due to the small number of studies (S6 Table and S2-S3 Figs). Efficacy against EV-A71 infection-related outcomes other than HFMD varied across studies. Estimates of efficacy against all EV-A71 related diseases ranged from 80.4% to 96.8%, while efficacy against herpangina and non-HFMD disease (i.e., upper respiratory tract infection and diarrhoea) was not statistically significant. For severe outcomes, efficacy was reported at 100% against severe EV-A71 HFMD and EV-A71 hospitalization (Fig 3B).

### Vaccine effectiveness

In total, 8 studies were eligible for evaluating the effectiveness of inactivated EV-A71 vaccines in real-world settings, including 5 test-negative design (TND) case-control studies [17–21], 1 case-control study [22], and 2 cohort studies [23,24]. These studies were conducted in southwest, middle, north and east China (Fig 2B). Among these, 5 studies focused on the time period between 2017 and 2018 [17–19,23,24], corresponding to 1–2 years after the initiation of the EV-A71 vaccination programme, and 1 study was conducted in 2019 [21]. Only 2 studies had a timeframe exceeding five years. One conducted from 2017 to 2022, with most participants receiving vaccines within a year before their hospital visits [20]. The other study spanned from 2011 to 2021 and included participants diagnosed before 2016, prior to the market availability of the vaccines [22] (Fig 2C). The data sources for these studies predominantly included the Chinese HFMD national surveillance network and China CDC immunization information system. One study obtained data through a questionnaire survey administered to HFMD patients and their parents. All studies relied on laboratory confirmation of outcomes, utilizing techniques such as RT-PCR. The detailed information of the eligible studies is shown in Table 2.

**Table 2:** Characteristics of selected studies reporting on the effectiveness of EV-A71 inactivated vaccines.

Author	Study location	Study period	Data source	Design	Sample size	Participant age	Case confirmation	Negative control	Vaccine	Reference/ unvaccinated group
Li Y (2019) [17]	Zhengzhou, China	2017.2-2018.2	Medical records and questionnaire survey	TND	1,803	6-71 months	RT-PCR	tested negative for EV-A71	All inactivated EV-A71 vaccines	unvaccinated
Jiang LN (2019) [18]	Guangxi, China	2017.1-2018.12	Local CDC and electronic immunization system	TND	2,779	0-12 years	RT-PCR	tested negative for EV-A71 or tested positive for CV-A16 or pan-enterovirus	All inactivated EV-A71 vaccines	unvaccinated
Wang XL (2019) [19]	Beijing, China	2017.1-2017.12	National notifiable infectious diseases reporting information system and Beijing management system of information for the immunization program	TND	2,184	6-59 months	RT-PCR	tested negative for EV-A71 or tested positive for non-EV-A71	All inactivated EV-A71 vaccines	unvaccinated
Duan XX (2024) [20]	Chengdu, China	2017.6-2022.3	Clinical electronic medical records system and Sichuan management system of information for the immunization program	TND	4,883	6 months and over	RT-PCR	tested negative for EV-A71	All inactivated EV-A71 vaccines	unvaccinated
Zhang YT (2024) [21]	Henan, Hunan, and Yunnan, China	2019.1-2019.12	Sentinel hospitals, local CDCs, and vaccination information registration system, vaccination record book, and parental self-report	TND	3,223	6-71 months	RT-PCR	tested negative for EV-A71	All inactivated EV-A71 vaccines	unvaccinated
Wang J (2023) [22]	Shanghai	2011.1-2021.12	China CDC information system and Shanghai immunization information system	Case-control	146	2 months to 26 years old	RT-PCR	tested negative for EV-A71 and positive for other enterovirus	All inactivated EV-A71 vaccines	unvaccinated and partially vaccinated
Guan XH (2019) [23]	Hubei, China	2016.9-2017.12	Notifiable infectious diseases network and childhood immunization information management system	Cohort	155,995	6-71 months	q-RT-PCR	–	CAMS vaccine	unvaccinated and partially vaccinated
Hua RJ (2021) [24]	Shanghai, China	2017.10-2017.12	Local CDC and immunization clinics data	Cohort	3,018	8-20 months	PCR	–	CAMS vaccine	unvaccinated

**Fig 4:**
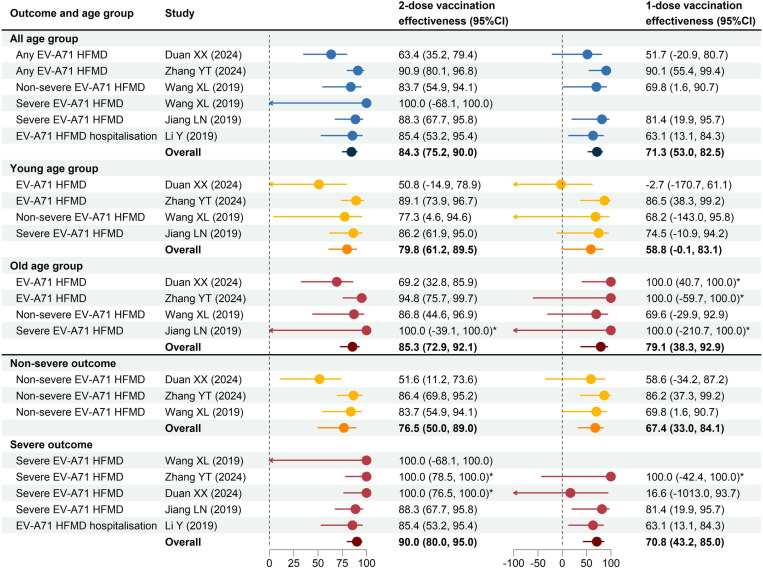
Estimates of vaccine effectiveness against EV-A71 related HFMD outcomes. The blue dots refer to the effectiveness against any EV-A71-HFMD among individuals aged from 0 to 12 years; the yellow dots refer to the effectiveness among the younger age groups (including 6-23 months, 6-35 months and 6-36 months) and against non-severe outcomes; the red dots refer to effectiveness among the older age groups (including 24-71 months, 24 months and over, 36-59 months and 37-60 months) and against severe outcomes. The overall effectiveness was estimated using random-effects models. * For these estimates of effectiveness of 100% without reporting a 95% CI, we provided a 95% CI calculated by Fisher’s exact test.

We selected only those TND studies that used unvaccinated individuals as the reference group to compare the estimates of effectiveness against EV-A71 related HFMD (Fig 4). The estimated overall effectiveness of 2-dose vaccination against any EV-A71-related HFMD across five studies was 84.3% (95% CI: 75.2, 90.0) among children aged 0–12 years. Effectiveness was observed to be higher among older children compared to younger children across all outcomes evaluated, estimated at 85.3% (95% CI: 72.9, 92.1) and 79.8% (95% CI: 61.2, 89.5), respectively. Additionally, 2-dose vaccination effectiveness was higher against severe HFMD compared to non-severe HFMD, with overall effectiveness estimated at 90.0% (95% CI: 80.0, 95.0) and 76.5% (95% CI: 50.0, 89.0), respectively. The effectiveness of a single-dose vaccination was observed to be low, with an overall effectiveness of 71.3% against any EV-A71-HFMD, 67.4% against non-severe EV-A71-HFMD and 70.8% against severe EV-A71-HFMD. However, most of the estimates of one-dose effectiveness from these studies displayed less significance. Low heterogeneity was found in most analyses except for the 2-dose vaccination effectiveness against non-severe HFMD, where moderate heterogeneity was observed (I² = 63%, P = 0.07). The detailed meta-analysis results and estimated heterogeneity can be found in S6 Table and S4–S8 Figs. No publication bias was observed (S6 Table and S9 and S10 Figs).

### Sensitivity analysis

We also estimated the overall efficacy and effectiveness using fixed-effect models, assuming no heterogeneity across studies. The point estimates were close to those estimated with the random-effects models, but had narrower confidence intervals. Detailed results for the fixed-effects models can be found in S2 and S4–S8 Figs.

## Discussion

This systematic review provides evidence that the inactivated EV-A71 vaccines, both in phase III trials and after licensure, confer significant levels of protection against EV-A71 HFMD. Clinical trials have demonstrated an overall efficacy of over 95% against EV-A71 related HFMD after one year of follow-up, and the efficacy of these vaccines remained consistently high two years post-vaccination, without showing signs of attenuation over time during those first two years. However, in the 1-year follow-up studies, all possible patients with any medically significant symptoms or illness were tested for EV-A71 during follow-up, whereas in the 2-year extended follow-up studies, only HFMD-like patients were tested for EV-A71. This discrepancy may have led to underreporting of HFMD cases with mild or atypical symptoms and an overestimate of efficacy two years post-vaccination. In our meta-analysis, we were not able to formally test a decline in efficacy or effectiveness over time due to limited data. However, studies [21] conducted in the early years of EV-A71 vaccination programme reported a higher level of vaccine protection compared to a study [20] that included more recent years, with effectiveness against HFMD reported as 90.0% and 63.4%, respectively. Additionally, Duan’s study revealed a decrease in effectiveness beyond 6 months post-vaccination [20]. The effectiveness against EV-A71 associated HFMD was 68.0% among children who completed full vaccination within 6 months before admission, compared with 55.6% in children who completed full vaccination more than 6 months prior. Further research is needed to evaluate the duration of vaccine protection as well as its long-term effectiveness and impact.

EV-A71 vaccines have demonstrated greater effect in protecting against severe outcomes than mild ones, as supported by evidence from both clinical trials and real-world studies. While the efficacy in RCTs against mild manifestations, such as herpangina, upper respiratory tract infections, and diarrhoea, was relatively low and not statistically significant, the vaccines showed high efficacy in preventing severe cases of HFMD and hospitalizations, reaching up to 100%. Consistently, the real-world effectiveness of EV-A71 vaccines in preventing non-severe HFMD was lower compared to the effectiveness against severe HFMD and hospitalizations. This highlights the notable protection offered by inactivated EV-A71 vaccines in preventing the progression of diseases caused by EV-A71 infection. Inactivated vaccines induce specific antibodies and memory cells, which effectively neutralize and eliminate the virus upon exposure [25]. By reducing viral load, this mechanism may lower the likelihood of EV-A71 crossing the blood-brain barrier and potentially reduce the risk of developing severe disease and death. However, further investigation is needed to confirm this effect.

In this review, we found that older children tend to exhibit higher protection from EV-A71 vaccines compared to younger children. Inactivated vaccines generally establish a stable and long-lasting memory immune response after vaccination in infants and children. However, the seropositivity after vaccination varied by age group, and seroconversion increased with age [26]. Moreover, the seroprevalence of EV-A71 antibody before vaccination also increased with age, from 26% at 1 year to 70% at 5 years [27]. Both the high seropositivity before and after vaccination among older children may be contributed to the high effectiveness of EV-A71 vaccines in this age group. In addition, compared to infants and young children, older children have a more mature immune system, enabling a stronger response to both infection and vaccine-induced immunity. Earlier exposure to similar pathogens in older children also facilitates the rapid development of memory B cells, leading to a more robust immune response upon re-exposure [28]

Compared to the efficacy observed in clinical trials, the effectiveness of EV-A71 vaccines in real-world settings was lower and at a moderately high level, around 80%. Real world scenarios present a more complicated context, characterized by heterogeneous populations, varying circulation intensity of EV-A71, and deviations from the recommended vaccination schedule, which are rigidly controlled in clinical trials [29]. As a result, clinical trials provide an ideal estimate of efficacy at the individual level. However, different study designs in real-world settings can yield varying estimates of effectiveness, influenced by factors such as study period, age of participants, definition of vaccination status, and criteria to define cases. Especially, vaccination coverage was positively associated with the effectiveness of vaccines. A real-world study conducted across China reported that the relative risk of EV-71 vaccination on EV-A71-HFMD incidence was rapidly decreased to below 0.71 when vaccination coverage exceeded 20% [30]. Imperfect recording of vaccination status or poor laboratory test specificity can also lead to underestimation of vaccine effectiveness [31]. Despite these limitations, observational studies with same time period included in this review reported close estimates of EV-A71 vaccine effectiveness.

EV-A71 inactivated vaccines exhibit cross-protection against infections caused by different sub-genotypes of EV-A71. One study demonstrated that C4-based EV-A71 vaccines can induce a broad spectrum of cross-neutralizing antibodies against EV-A71 sub-genotypes A, B, and C [32]. Recently, a B4-strain based EV-A71 vaccine in Taiwan has reached phase III stage and showed high level protection against EV-A71 associated HFMD [16]. The B4-based vaccines have also shown the capacity to cross-protect against B5 and C4 genotypes [16,33]. The cross-protection provided by EV-A71 vaccines against sub-genotypes within EV-A71 enhances their versatility in combating EV-A71 infections and they will likely offer protection against future new EV-A71 genotypes. The significant cross-protection observed among EV-A71 sub-genotypes provides evidence supporting the approval and global use of inactivated EV-A71 vaccines. This is particularly relevant in countries such as Vietnam, Thailand, Japan, and Malaysia, where a significant number of cases and outbreaks have been attributed to B5 and C4 strains of EV-A71 [34–36].

EV-A71 inactivated vaccines however do not confer cross-protection against infections caused by other enterovirus serotypes that cause HFMD, such as CVA16, 6, and 10 [17,20]. Since 2013, CVA6, CVA10 and other enterovirus serotypes have become the predominant circulating strains and have caused the majority of HFMD cases in mainland China [37,38]. Therefore, there is an urgent need for research and development of bivalent and multivalent vaccines to prevent HFMD caused by various enterovirus serotypes, including EV-A71. Recently, efforts have been made to create bivalent and multivalent vaccines, such as the EV-A71/CVA16 bivalent vaccine, the EV-A71/CVA6/CVA10 trivalent vaccine, and the EV-A71/CVA16/CVA6/CVA10 tetravalent vaccine [39]. However, these vaccines are currently in the preclinical stage of development.

Although EV-A71 inactivated vaccines have been approved for marketing, they come with a high cost, often exceeding 20 USD per dose. While this price is lower than that of some privately available vaccines, such as the rotavirus vaccine ($38 per dose), it contrasts with vaccines included in the national immunization programs, which are provided free of charge. This affects their uptake in the private sector and can limit access in lower-income populations in the absence of a public program. Ensuring equitable access to the EV-A71 vaccine is essential for controlling HFMD outbreaks and protecting vulnerable populations. Government subsidies and inclusion in the national immunization programs could help make the vaccine more affordable and accessible, thereby maximizing its public health impact.

Vaccine safety and the potential for adverse reactions are key factors influencing vaccination uptake intentions. Both clinical trials [11,13] and real-world study [40] have demonstrated the good safety profile of inactivated EV-A71 vaccines, with only mild adverse events reported, including fever, localized redness, induration, pain, swelling, and pruritus. These reactions were generally transient and typically resolved within three days without the need for medical intervention. The high effectiveness and strong safety profile of the inactivated EV-A71 vaccines could serve as important drivers for vaccine advocacy, promoting higher coverage rates among all susceptible populations.

There are several limitations to this study. Firstly, due to the small number of studies available and their primary focus on the initial 1 or 2 years of the vaccination programme, coupled with lack of detailed information of protection duration after vaccination, we were unable to statistically estimate the effectiveness of EV-A71 vaccines over time. Secondly, the limited number of studies didn’t allow us to conduct a meta-regression to identify factors associated with vaccine effectiveness and to quantify heterogeneity potentially influenced by moderators such as follow-up duration and study region. These two moderators may have contributed to the moderate heterogeneity observed in some subgroup analyses. The small number of include studies also reduced statistical power in our analysis, and further research is needed to support the subgroup analysis.

The existing evidence regarding the effectiveness of EV-A71 vaccines primarily focuses on the initial 1–2 years of vaccination programme, with a significant gap in research on effectiveness beyond the first two years. Since children remain susceptible before the age of 5, it is crucial to evaluate the long-term protection over 2 years provided by these vaccines. Further research is critically needed to assess long-term vaccine effectiveness and impact, as well as the potential immune waning and need for booster doses.

## Conclusions

Existing EV-A71 vaccine efficacy and effectiveness studies have shown high protection against HFMD and severe complications. However, they have primarily concentrated on evaluating protection within the initial two years post-implementation of the vaccination programme. The long-term effectiveness of these vaccines remains uncertain and it is unclear if booster doses might improve effectiveness against HFMD in older children. Further research is imperative to ascertain the duration of immune protection against EV-A71-related diseases over an extended period, and to ensure sustained protection in the future.

## Supporting information

S1 Table Search strategy. (DOCX)

S2 Table PRISMA 2020 checklist. (DOCX)

S3 Table All studies identified in the literature search. (DOCX)

S4 Table All data extracted from the primary research sources for the systematic review and meta-analysis. (DOCX)

S5 Table Quality assessment for observational studies according to ROBINS-I. (DOCX)

S6 Table Diagnostic checks for assumptions of random-effects model. (DOCX)

S1 Fig Quality assessment for RCTs studies according to Cochrane RoB tool. (TIF)

S2 Fig Meta-analysis of studies on efficacy of EV-A71 vaccines against HFMD by duration of the follow-up period. (TIF)

S3 Fig Funnel plot of studies on efficacy of EV-A71 vaccines against HFMD by duration of the follow-up period. (TIF)

S4 Fig Meta-analysis of studies on effectiveness of EV-A71 vaccines against any HFMD among all age groups. (TIF)

S5 Fig Meta-analysis of studies on effectiveness of EV-A71 vaccines against non-severe HFMD among all age groups. (TIF)

S6 Fig Meta-analysis of studies on effectiveness of EV-A71 vaccines against severe HFMD among all age groups. (TIF)

S7 Fig Meta-analysis of studies on effectiveness of EV-A71 vaccines against any HFMD among the young age. (TIF)

S8 Fig Meta-analysis of studies on effectiveness of EV-A71 vaccines against any HFMD among the old age group. (TIF)

S9 Fig Funnel plot of studies on effectiveness of EV-A71 vaccines against HFMD by severity and doses. (TIF)

S10 Fig Funnel plot of studies on effectiveness of EV-A71 vaccines against any HFMD by age group and doses. (TIF)
