# Supplementary material for: The efficacy and effectiveness of enterovirus A71 vaccines against hand, foot, and mouth disease: A systematic review and meta-analysis
Source: PLoS One. 2025 May 22;20(5):e0323782. doi: 10.1371/journal.pone.0323782 (PMC12097632; doi:10.1371/journal.pone.0323782)
Supplement: S1 Table — (DOCX) [file pone.0323782.s001.docx]

**Supporting information**

**The efficacy and effectiveness of enterovirus A71 vaccines against hand, foot, and mouth disease: a systematic review and meta-analysis**

# **S1 Table. Search strategy**

| **PubMed** | | |
| --- | --- | --- |
| **STEP** | **TERM (all field)** | |
| #1 | EV-A71 OR EV A71 OR EVA71 | |
| #2 | EV71 OR EV 71 | |
| #3 | enterovirus 71 | |
| #4 | Enterovirus A71 | |
| #5 | hand, foot, and mouth disease | |
| #6 | HFMD | |
| #7 | #1 OR #2 OR #3 OR #4 OR #5 OR #6 | |
| #8 | vaccine OR vaccination | |
| #9 | efficacy | |
| #10 | protection | |
| #11 | immunization | |
| #12 | impact | |
| #13 | effectiveness | |
| #14 | #9 OR #10 OR #11 OR #12 OR #13 | |
| #15 | Language: English | |
| #16 | #7 AND #8 AND #14 AND #15 | |
| **Web of Science Core Collection** | | |
| **STEP** | **TERM** | |
| #1 | ALL=(EV-A71) OR ALL=(EV A71) OR ALL=(EVA71) | |
| #2 | ALL=(EV71) OR ALL=(EV 71) | |
| #3 | ALL=(enterovirus 71) | |
| #4 | ALL=(Enterovirus A71) | |
| #5 | ALL=(hand, foot, and mouth disease) | |
| #6 | ALL=(HFMD) | |
| #7 | #1 OR #2 OR #3 OR #4 OR #5 OR #6 | |
| #8 | ALL=(vaccine) OR ALL=(vaccination) | |
| #9 | ALL=(efficacy) | |
| #10 | ALL=(protection) | |
| #11 | ALL=(immunization) | |
| #12 | ALL=(impact) | |
| #13 | ALL=(effectiveness) | |
| #14 | #9 OR #10 OR #11 OR #12 OR #13 | |
| #15 | LA=(English) | |
| #16 | #7 AND #8 AND #14 AND #15 | |
| **Elsevier ScienceDirect** | | |
| **STEP** | **TERM** | |
| #1 | Title, abstract, keywords: ((EV-A71) OR (EV A71) OR (EVA71)) AND ((vaccine) OR (vaccination)) AND ((efficacy) OR (protection) OR (impact) OR (effectiveness)) | |
| #2 | Title, abstract, keywords: ((EV71) OR (EV 71) OR (enterovirus 71)) AND ((vaccine) OR (vaccination)) AND ((efficacy) OR (protection) OR (impact) OR (effectiveness)) | |
| #3 | Title, abstract, keywords: ((hand, foot, and mouth disease) OR (HFMD)) AND ((vaccine) OR (vaccination)) AND ((efficacy) OR (protection) OR (impact) OR (effectiveness)) | |
| #4 | language: English | |
| **CNKI** (<https://chn.oversea.cnki.net/index/>) | | |
| **STEP** | **TERM** | **Match** |
| 1# | Title, Keyword and Abstract: EV71灭活疫苗 + EV71疫苗 + 手足口 + 手足口疫苗 | fuzzy |
| 2# | Title, Keyword and Abstract: 接种效果 + 保护效果 + 预防效果 + 接种作用 + 保护作用 + 预防作用 | fuzzy |
| 3# | 1# AND 2# |  |
| 4# | Source type：PKU core journals, CSSCI and CSCD |  |
| **Wanfang Data** (<https://www.wanfangdata.com.cn/index.html>) | |  |
| **STEP** | **TERM** | **Match** |
| 1# | All field: EV71灭活疫苗 OR EV71疫苗 OR 手足口 OR 手足口疫苗 | fuzzy |
| 2# | All field: 接种效果 OR 保护效果 OR 预防效果 OR 接种作用 OR 保护作用 OR 预防作用 | fuzzy |
| 3# | 1# AND 2# |  |
| 4# | Core journal：PKU core journals, CSSCI and CSCD |  |

Note: Fuzzy matching means that the search term is segmented and then matched in the title, abstract, and full text.

The search timeframe was from the beginning up to May 20, 2024.
