# Supplementary material for: The efficacy and effectiveness of enterovirus A71 vaccines against hand, foot, and mouth disease: A systematic review and meta-analysis
Source: PLoS One. 2025 May 22;20(5):e0323782. doi: 10.1371/journal.pone.0323782 (PMC12097632; doi:10.1371/journal.pone.0323782)
Supplement: S3 Table — (DOCX) [file pone.0323782.s003.docx]

**Supporting information**

**The efficacy and effectiveness of enterovirus A71 vaccines against hand, foot, and mouth disease: a systematic review and meta-analysis**

# **S3 Table. All studies identified in the literature search**

| No. | Study | DOI | Language | exclusion/inclusion |
| --- | --- | --- | --- | --- |
| 1 | Xu 2017 | 10.1016/j.antiviral.2016.11.008 | English | title-abstract exclusion |
| 2 | Lalani 2021 | 10.1016/j.lfs.2021.120097 | English | title-abstract exclusion |
| 3 | Mandary 2018 | 10.3390/v10060320 | English | title-abstract exclusion |
| 4 | Chen 2019 | 10.1186/s12929-019-0596-8 | English | title-abstract exclusion |
| 5 | Ding 2022 | 10.1080/21645515.2022.2121565 | English | title-abstract exclusion |
| 6 | Yang 2017 | 10.2807/1560-7917.ES.2017.22.50.16-00824 | English | title-abstract exclusion |
| 7 | Hu 2022 | 10.1186/s13099-022-00485-1 | English | title-abstract exclusion |
| 8 | Lin 2023 | 10.1186/s12929-023-00987-3 | English | title-abstract exclusion |
| 9 | Yee 2017 | 10.1016/j.virol.2017.03.017 | English | title-abstract exclusion |
| 10 | Lalani 2021 | 10.1016/j.peptides.2020.170443 | English | title-abstract exclusion |
| 11 | Lian 2023 | 10.1016/j.bsheal.2023.05.002 | English | title-abstract exclusion |
| 12 | Chen 2017 | 10.1089/vim.2016.0110 | English | title-abstract exclusion |
| 13 | Cheng 2023 | 10.1016/j.antiviral.2023.105569 | English | title-abstract exclusion |
| 14 | VanTan 2015 | 10.1016/j.jviromet.2015.02.011 | English | title-abstract exclusion |
| 15 | Yang 2020 | 10.1080/21645515.2019.1709353 | English | title-abstract exclusion |
| 16 | Liu 2020 | 10.1002/jmv.25657 | English | title-abstract exclusion |
| 17 | Tang 2021 | 10.1016/j.ijid.2021.03.039 | English | title-abstract exclusion |
| 18 | Chang 2015 | 10.1186/s12866-015-0474-9 | English | title-abstract exclusion |
| 19 | Yang 2019 | 10.1093/aje/kwy238 | English | title-abstract exclusion |
| 20 | Peng 2023 | 10.1186/s12879-023-08109-y | English | title-abstract exclusion |
| 21 | Cai 2019 | 10.1186/s12879-019-3878-6 | English | title-abstract exclusion |
| 22 | Fang 2022 | 10.1080/17460441.2021.1965987 | English | title-abstract exclusion |
| 23 | Yee 2018 | 10.7150/ijms.26450 | English | title-abstract exclusion |
| 24 | You 2023 | 10.1371/journal.ppat.1011420 | English | title-abstract exclusion |
| 25 | Tan 2017 | 10.1016/j.virol.2016.11.009 | English | title-abstract exclusion |
| 26 | Le 2022 | 10.3389/fmicb.2022.987801 | English | title-abstract exclusion |
| 27 | NikNadia 2016 | 10.1371/journal.pntd.0004562 | English | title-abstract exclusion |
| 28 | Geoghegan 2015 | 10.1128/JVI.00706-15 | English | title-abstract exclusion |
| 29 | Lim 2015 | 10.1016/j.vaccine.2015.05.108 | English | title-abstract exclusion |
| 30 | Hoang 2019 | 10.1016/j.ijid.2018.12.004 | English | title-abstract exclusion |
| 31 | Puenpa 2020 | 10.1016/j.vaccine.2020.10.002 | English | title-abstract exclusion |
| 32 | Chua 2021 | 10.3390/v13030438 | English | title-abstract exclusion |
| 33 | Cheong 2023 | 10.1016/j.antiviral.2023.105713 | English | title-abstract exclusion |
| 34 | Long 2016 | 10.1038/srep23444 | English | title-abstract exclusion |
| 35 | Lee 2021 | 10.1016/j.vaccine.2021.02.024 | English | title-abstract exclusion |
| 36 | Aw-Yong 2016 | 10.1371/journal.pone.0165659 | English | title-abstract exclusion |
| 37 | Chen 2023 | 10.1007/s11033-022-08116-3 | English | title-abstract exclusion |
| 38 | Boussettine 2021 | 10.22207/JPAM.15.4.06 | English | title-abstract exclusion |
| 39 | Upala 2018 | 10.1186/s12879-018-3560-4 | English | title-abstract exclusion |
| 40 | Tan 2023 | 10.3390/vaccines11081363 | English | title-abstract exclusion |
| 41 | Tang 2020 | 10.1016/j.ejmech.2020.112310 | English | title-abstract exclusion |
| 42 | Apostol 2019 | 10.1186/s12879-019-3955-x | English | title-abstract exclusion |
| 43 | Phyu 2017 | 10.1038/emi.2017.49 | English | title-abstract exclusion |
| 44 | Hsieh 2024 | 10.1128/jvi.01558-23 | English | title-abstract exclusion |
| 45 | Liu 2023 | 10.1016/j.virusres.2023.199235 | English | title-abstract exclusion |
| 46 | Li 2015 | 10.1016/j.vaccine.2014.10.081 | English | title-abstract exclusion |
| 47 | Lin 2018 | 10.1016/j.jtice.2017.10.020 | English | title-abstract exclusion |
| 48 | Zhao 2015 | 10.1038/srep07878 | English | title-abstract exclusion |
| 49 | Wang 2021 | 10.1038/s41598-021-84891-6 | English | title-abstract exclusion |
| 50 | Zhang 2016 | 10.1089/vim.2015.0110 | English | title-abstract exclusion |
| 51 | Anasir 2019 | 10.3390/ijms20061256 | English | title-abstract exclusion |
| 52 | Hoa-Tran 2020 | 10.1016/j.vaccine.2020.11.031 | English | title-abstract exclusion |
| 53 | Qiao 2023 | 10.1016/j.jiac.2022.12.006 | English | title-abstract exclusion |
| 54 | Liu 2020 | 10.1016/j.cmi.2019.06.029 | English | title-abstract exclusion |
| 55 | Phyu 2016 | 10.1371/journal.pone.0147463 | English | title-abstract exclusion |
| 56 | Wu 2021 | 10.1080/22221751.2021.1934558 | English | title-abstract exclusion |
| 57 | Cheng 2024 | 10.3389/fpubh.2024.1336687 | English | title-abstract exclusion |
| 58 | Liu 2023 | 10.1016/j.antiviral.2023.105583 | English | title-abstract exclusion |
| 59 | NikNadia 2016 | 10.1371/journal.pone.0148767 | English | title-abstract exclusion |
| 60 | Lien 2022 | 10.1016/j.chroma.2022.463427 | English | title-abstract exclusion |
| 61 | Liu 2016 | 10.1016/j.vaccine.2015.12.026 | English | title-abstract exclusion |
| 62 | Huang 2018 | 10.3201/eid2403.171303 | English | title-abstract exclusion |
| 63 | Yi 2023 | 10.4062/biomolther.2023.058 | English | title-abstract exclusion |
| 64 | Nhan 2019 | 10.1093/ofid/ofz284 | English | title-abstract exclusion |
| 65 | Yee 2019 | 10.1038/s41598-019-41285-z | English | title-abstract exclusion |
| 66 | Kingston 2022 | 10.1128/msphere.00088-22 | English | title-abstract exclusion |
| 67 | Zhu 2016 | 10.1016/j.jconrel.2016.10.019 | English | title-abstract exclusion |
| 68 | Mao 2015 | 10.1080/21645515.2015.1011975 | English | title-abstract exclusion |
| 69 | Nhan 2020 | 10.1371/journal.pntd.0008544 | English | title-abstract exclusion |
| 70 | Liu 2021 | 10.1093/femspd/ftab057 | English | title-abstract exclusion |
| 71 | Zhu 2021 | 10.1016/j.vaccine.2021.09.056 | English | title-abstract exclusion |
| 72 | Nguyet 2020 | 10.3201/eid2602.190721 | English | title-abstract exclusion |
| 73 | Tsai 2019 | 10.1128/JVI.02308-18 | English | title-abstract exclusion |
| 74 | Wang 2017 | 10.3390/ijerph14010033 | English | title-abstract exclusion |
| 75 | Wang 2016 | 10.1017/S0950268815001569 | English | title-abstract exclusion |
| 76 | Caine 2015 | 10.3390/v7112916 | English | title-abstract exclusion |
| 77 | Romanenkova 2023 | 10.3390/vaccines11050931 | English | title-abstract exclusion |
| 78 | Huang 2020 | 10.1371/journal.ppat.1008857 | English | title-abstract exclusion |
| 79 | Chen 2016 | 10.1017/S0950268815001107 | English | title-abstract exclusion |
| 80 | Takahashi 2018 | 10.1098/rsif.2018.0507 | English | title-abstract exclusion |
| 81 | Le 2022 | 10.1016/j.heliyon.2022.e12042 | English | title-abstract exclusion |
| 82 | Wang 2021 | 10.1016/j.vaccine.2021.06.031 | English | title-abstract exclusion |
| 83 | Mirand 2016 | 10.3201/eid2211.160590 | English | title-abstract exclusion |
| 84 | Lyu 2015 | 10.1007/s11434-015-0847-3 | English | title-abstract exclusion |
| 85 | Liang 2013 | 10.1007/s11684-012-0237-z | English | title-abstract exclusion |
| 86 | Tan 2014 | 10.1186/1423-0127-21-14 | English | title-abstract exclusion |
| 87 | Gao 2018 | 10.1093/cid/ciy341 | English | title-abstract exclusion |
| 88 | Liang 2011 | 10.1016/j.vaccine.2011.10.018 | English | title-abstract exclusion |
| 89 | Yang 2020 | 10.1080/21645515.2019.1649554 | English | title-abstract exclusion |
| 90 | Liu 2014 | 10.1016/j.vaccine.2014.08.064 | English | title-abstract exclusion |
| 91 | Ngo-My 2023 | 10.1007/s40995-023-01489-5 | English | title-abstract exclusion |
| 92 | Fan 2021 | 10.1016/j.vaccine.2020.11.070 | English | title-abstract exclusion |
| 93 | Zarif 2021 | 10.1016/j.virusres.2021.198456 | English | title-abstract exclusion |
| 94 | Issaro 2019 | 10.1016/j.molimm.2018.09.018 | English | title-abstract exclusion |
| 95 | Sun 2024 | 10.1002/jmv.29412 | English | title-abstract exclusion |
| 96 | Li 2022 | 10.3390/v14061142 | English | title-abstract exclusion |
| 97 | Wei 2021 | 10.1016/S1473-3099(20)30480-1 | English | title-abstract exclusion |
| 98 | Li 2018 | 10.1016/j.vaccine.2018.05.054 | English | title-abstract exclusion |
| 99 | Rattanapisit 2019 | 10.3390/plants8120560 | English | title-abstract exclusion |
| 100 | Wu 2022 | 10.1371/journal.pone.0270061 | English | title-abstract exclusion |
| 101 | Lee 2010 | 10.1016/j.vaccine.2010.09.065 | English | title-abstract exclusion |
| 102 | Deng 2020 | 10.1016/j.vaccine.2020.03.023 | English | title-abstract exclusion |
| 103 | Huang 2015 | 10.1128/JVI.02035-15 | English | title-abstract exclusion |
| 104 | Chang 2015 | 10.1007/s12026-015-8661-1 | English | title-abstract exclusion |
| 105 | Xu 2018 | 10.1186/s12879-018-3509-7 | English | title-abstract exclusion |
| 106 | Cao 2015 | 10.3892/mmr.2015.3680 | English | title-abstract exclusion |
| 107 | Xie 2016 | 10.1016/j.virusres.2015.11.007 | English | title-abstract exclusion |
| 108 | Chen 2019 | 10.3389/fimmu.2019.02564 | English | title-abstract exclusion |
| 109 | Tamura 2022 | 10.1016/j.vaccine.2022.04.064 | English | title-abstract exclusion |
| 110 | Nayak 2022 | 10.1186/s43088-022-00258-4 | English | title-abstract exclusion |
| 111 | Zhang 2018 | 10.1016/j.jcv.2018.06.001 | English | title-abstract exclusion |
| 112 | Yang 2017 | 10.1186/s12985-017-0872-8 | English | title-abstract exclusion |
| 113 | Zhang 2016 | 10.1080/21645515.2015.1115934 | English | title-abstract exclusion |
| 114 | Li 2018 | 10.1007/s12519-018-0189-8 | English | title-abstract exclusion |
| 115 | Xu 2012 | 10.1016/j.bbrc.2012.03.067 | English | title-abstract exclusion |
| 116 | Nicholson 2017 | 10.1093/infdis/jix493 | English | title-abstract exclusion |
| 117 | Sun 2014 | 10.4161/hv.29823 | English | title-abstract exclusion |
| 118 | Zhao 2015 | 10.1017/S095026881500028X | English | title-abstract exclusion |
| 119 | Liu 2020 | 10.1186/s12985-020-01328-8 | English | title-abstract exclusion |
| 120 | Yu 2018 | 10.1371/journal.pone.0203792 | English | title-abstract exclusion |
| 121 | Sun 2015 | 10.1080/21645515.2015.1053675 | English | title-abstract exclusion |
| 122 | Hu 2015 | 10.1097/INF.0000000000000794 | English | title-abstract exclusion |
| 123 | Zhou 2015 | 10.1002/jmv.24018 | English | title-abstract exclusion |
| 124 | Chong 2012 | 10.4161/hv.21739 | English | title-abstract exclusion |
| 125 | Hu 2021 | 10.1021/acs.jmedchem.1c00758 | English | title-abstract exclusion |
| 126 | Lai 2016 | 10.1017/S0950268815002630 | English | title-abstract exclusion |
| 127 | Gan 2015 | 10.1080/21645515.2015.1059980 | English | title-abstract exclusion |
| 128 | Zhang 2015 | 10.1016/j.vaccine.2015.08.016 | English | title-abstract exclusion |
| 129 | Mustafa 2020 | 10.3390/vaccines8040742 | English | title-abstract exclusion |
| 130 | Zhou 2016 | 10.1016/j.vaccine.2016.04.029 | English | title-abstract exclusion |
| 131 | Ye 2021 | 10.1186/s12889-021-11198-6 | English | title-abstract exclusion |
| 132 | Chung 2008 | 10.1016/j.vaccine.2008.01.058 | English | title-abstract exclusion |
| 133 | Zhang 2014 | 10.1186/1743-422X-11-80 | English | title-abstract exclusion |
| 134 | Ch'ng 2011 | 10.1002/jmv.22198 | English | title-abstract exclusion |
| 135 | Ku 2014 | 10.1016/j.vaccine.2014.06.025 | English | title-abstract exclusion |
| 136 | Fan 2020 | 10.1016/j.vaccine.2019.12.057 | English | title-abstract exclusion |
| 137 | Dong 2010 | 10.4161/hv.6.12.12982 | English | title-abstract exclusion |
| 138 | Wang 2017 | 10.1371/journal.pntd.0005899 | English | title-abstract exclusion |
| 139 | Wang 2016 | 10.1016/j.biologicals.2016.01.003 | English | title-abstract exclusion |
| 140 | Nishimura 2015 | 10.1371/journal.ppat.1005184 | English | title-abstract exclusion |
| 141 | Lee 2012 | 10.1371/journal.pntd.0001737 | English | title-abstract exclusion |
| 142 | In 2017 | 10.1371/journal.pone.0178259 | English | title-abstract exclusion |
| 143 | Zhang 2019 | 10.1016/S2352-4642(19)30181-6 | English | title-abstract exclusion |
| 144 | Han 2014 | 10.4161/hv.29123 | English | title-abstract exclusion |
| 145 | Zhao 2013 | 10.1186/1471-2180-13-287 | English | title-abstract exclusion |
| 146 | Luo 2021 | 10.1016/j.vaccine.2021.05.093 | English | title-abstract exclusion |
| 147 | Qi 2019 | 10.1371/journal.pone.0225569 | English | title-abstract exclusion |
| 148 | Li 2021 | 10.1080/14760584.2021.1933451 | English | title-abstract exclusion |
| 149 | Mao 2010 | 10.3760/cma.j.issn.0366-6999.2010.13.012 | English | title-abstract exclusion |
| 150 | Li 2012 | 10.1016/j.vaccine.2012.03.010 | English | title-abstract exclusion |
| 151 | Wu 2017 | 10.1016/j.vaccine.2017.03.065 | English | title-abstract exclusion |
| 152 | Wu 2016 | 10.1371/journal.pmed.1001975 | English | title-abstract exclusion |
| 153 | Lyu 2015 | 10.1128/JVI.00422-15 | English | title-abstract exclusion |
| 154 | Mao 2018 | 10.2217/fmb-2018-0063 | English | title-abstract exclusion |
| 155 | Zhang 2015 | 10.1016/j.vaccine.2015.03.034 | English | title-abstract exclusion |
| 156 | Chiu 2012 | 10.1016/j.antiviral.2012.05.009 | English | title-abstract exclusion |
| 157 | Huo 2017 | 10.1016/j.vaccine.2017.10.101 | English | title-abstract exclusion |
| 158 | Yang 2022 | 10.3389/fpubh.2022.970880 | English | title-abstract exclusion |
| 159 | Kim 2019 | 10.1371/journal.pone.0210477 | English | title-abstract exclusion |
| 160 | Cao 2013 | 10.2478/v10136-012-0032-9 | English | title-abstract exclusion |
| 161 | Chen 2006 | 10.1016/j.vaccine.2005.12.047 | English | title-abstract exclusion |
| 162 | Dong 2011 | 10.1016/j.vaccine.2011.06.044 | English | title-abstract exclusion |
| 163 | Yang 2021 | 10.1016/j.vaccine.2021.05.058 | English | title-abstract exclusion |
| 164 | Chan 2011 | <https://www.researchgate.net/publication/236838801_Enterovirus_71_in_Malaysia_A_decade_later> | English | title-abstract exclusion |
| 165 | Lim 2018 | 10.1016/j.vaccine.2018.05.005 | English | title-abstract exclusion |
| 166 | Bek 2011 | 10.1016/j.vaccine.2011.04.070 | English | title-abstract exclusion |
| 167 | Tan 2012 | 10.1371/journal.pone.0034589 | English | title-abstract exclusion |
| 168 | Zhou 2019 | 10.1016/j.antiviral.2018.11.001 | English | title-abstract exclusion |
| 169 | Luo 2019 | 10.1093/tropej/fmz004 | English | title-abstract exclusion |
| 170 | Chen 2011 | 10.1016/j.vaccine.2011.01.094 | English | title-abstract exclusion |
| 171 | Ang 2016 | 10.1186/s12985-016-0567-6 | English | title-abstract exclusion |
| 172 | Chen 2012 | 10.1016/j.virol.2012.06.026 | English | title-abstract exclusion |
| 173 | Lin 2015 | 10.1002/bit.25625 | English | title-abstract exclusion |
| 174 | Kingston 2023 | 10.1099/jgv.0.001867 | English | title-abstract exclusion |
| 175 | Zeng 2015 | 10.1038/srep10550 | English | title-abstract exclusion |
| 176 | Chiu 2006 | 10.1016/j.micinf.2006.01.021 | English | title-abstract exclusion |
| 177 | Li 2013 | 10.1016/j.vaccine.2013.05.019 | English | title-abstract exclusion |
| 178 | Zhang 2018 | 10.1038/S41426-018-0094-1 | English | title-abstract exclusion |
| 179 | Jin 2021 | 10.1128/JVI.01743-20 | English | title-abstract exclusion |
| 180 | Yang 2020 | 10.1080/22221751.2020.1823889 | English | title-abstract exclusion |
| 181 | Wang 2016 | 10.1371/journal.pone.0162373 | English | title-abstract exclusion |
| 182 | Kim 2015 | 10.1016/j.vaccine.2015.10.103 | English | title-abstract exclusion |
| 183 | Zhong 2022 | 10.18502/ijph.v51i9.10563 | English | title-abstract exclusion |
| 184 | Kung 2014 | 10.1517/14712598.2014.935330 | English | title-abstract exclusion |
| 185 | Imura 2020 | 10.1128/JVI.01921-19 | English | title-abstract exclusion |
| 186 | Li 2016 | 10.1016/j.amc.2015.11.090 | English | title-abstract exclusion |
| 187 | Li 2020 | 10.2147/TCRM.5268130 | English | title-abstract exclusion |
| 188 | CollaborativeStudyGrp 2018 | 10.1016/j.biologicals.2018.03.001 | English | title-abstract exclusion |
| 189 | Wang 2020 | 10.1080/21645515.2020.1711678 | English | title-abstract exclusion |
| 190 | Huang 2016 | 10.1371/journal.pone.0148907 | English | title-abstract exclusion |
| 191 | Dai 2019 | 10.3390/v11070625 | English | title-abstract exclusion |
| 192 | Gong 2020 | 10.3390/vaccines8010061 | English | title-abstract exclusion |
| 193 | Liu 2010 | 10.1016/j.vaccine.2010.08.080 | English | title-abstract exclusion |
| 194 | Wu 2001 | 10.1016/S0264-410X(01)00385-1 | English | title-abstract exclusion |
| 195 | Liang 2013 | 10.4161/hv.24949 | English | title-abstract exclusion |
| 196 | Wang 2018 | 10.1128/JVI.01330-17 | English | title-abstract exclusion |
| 197 | Peng 2022 | 10.1186/s12889-022-13365-9 | English | title-abstract exclusion |
| 198 | Lee 2022 | 10.3390/ijms232112967 | English | title-abstract exclusion |
| 199 | Sun 2020 | 10.1186/s13099-020-00377-2 | English | title-abstract exclusion |
| 200 | Guang 2007 | 10.1016/j.virusres.2006.12.005 | English | title-abstract exclusion |
| 201 | Li 2018 | 10.1016/j.intimp.2017.11.042 | English | title-abstract exclusion |
| 202 | Wang 2013 | 10.1016/j.bbrc.2012.11.035 | English | title-abstract exclusion |
| 203 | Jiang 2015 | 10.1016/j.vaccine.2015.10.104 | English | title-abstract exclusion |
| 204 | Xu 2014 | 10.7150/thno.7457 | English | title-abstract exclusion |
| 205 | Ong 2010 | 10.1128/JVI.00999-09 | English | title-abstract exclusion |
| 206 | Zheng 2017 | 10.1371/journal.pone.0184266 | English | title-abstract exclusion |
| 207 | Wang 2023 | 10.1016/j.vaccine.2023.08.083 | English | title-abstract exclusion |
| 208 | Wu 2023 | 10.1016/j.virs.2023.01.006 | English | title-abstract exclusion |
| 209 | Tan 2023 | 10.1016/j.heliyon.2023.e18212 | English | title-abstract exclusion |
| 210 | Zhang 2018 | 10.1016/j.antiviral.2018.02.002 | English | title-abstract exclusion |
| 211 | Vo-Nguyen 2021 | 10.1007/s40995-020-01025-9 | English | title-abstract exclusion |
| 212 | Yi 2023 | 10.1016/j.vaccine.2023.01.074 | English | title-abstract exclusion |
| 213 | Xue 2017 | 10.1016/j.bej.2016.10.009 | English | title-abstract exclusion |
| 214 | Huang 2015 | 10.1186/s12929-015-0181-8 | English | title-abstract exclusion |
| 215 | Xu 2015 | 10.1038/srep12973 | English | title-abstract exclusion |
| 216 | Gao 2019 | 10.3892/etm.2019.7529 | English | title-abstract exclusion |
| 217 | Yu 2000 | 10.1159/000025488 | English | title-abstract exclusion |
| 218 | Hwa 2013 | 10.1371/journal.pntd.0002538 | English | title-abstract exclusion |
| 219 | Kolpe 2012 | 10.1016/j.virusres.2012.06.014 | English | title-abstract exclusion |
| 220 | Xin 2010 | 10.1016/j.provac.2010.03.012 | English | title-abstract exclusion |
| 221 | Huang 2011 | 10.1258/ebm.2010.010233 | English | title-abstract exclusion |
| 222 | Ch'ng 2011 | 10.4149/av_2011_03_227 | English | title-abstract exclusion |
| 223 | Liao 2023 | 10.1080/21645515.2023.2209472 | English | title-abstract exclusion |
| 224 | Kirk 2012 | 10.1016/j.vaccine.2012.09.030 | English | title-abstract exclusion |
| 225 | Wang 2015 | 10.1016/j.vaccine.2015.09.043 | English | title-abstract exclusion |
| 226 | Zhang 2017 | 10.1128/JVI.02450-16 | English | title-abstract exclusion |
| 227 | Lin 2018 | 10.1016/j.vaccine.2018.05.090 | English | title-abstract exclusion |
| 228 | Sabovic 2019 | 10.15570/actaapa.2019.21 | English | title-abstract exclusion |
| 229 | Kang 2022 | 10.1038/s41401-021-00733-1 | English | title-abstract exclusion |
| 230 | Bek 2012 | 10.1007/s11908-012-0267-3 | English | title-abstract exclusion |
| 231 | Li 2018 | 10.3389/fmicb.2018.02337 | English | title-abstract exclusion |
| 232 | In 2018 | 10.1371/journal.pone.0202552 | English | title-abstract exclusion |
| 233 | Ku 2012 | 10.1016/j.jviromet.2012.06.025 | English | title-abstract exclusion |
| 234 | Ji 2023 | 10.1080/22221751.2023.2177084 | English | title-abstract exclusion |
| 235 | Lim 2012 | 10.1371/journal.pone.0029751 | English | title-abstract exclusion |
| 236 | Wang 2018 | 10.1007/s00705-018-3797-7 | English | title-abstract exclusion |
| 237 | Caine 2017 | 10.1128/JVI.01759-16 | English | title-abstract exclusion |
| 238 | Liou 2010 | 10.1016/j.vaccine.2010.09.089 | English | title-abstract exclusion |
| 239 | Yang 2015 | 10.1097/INF.0000000000000900 | English | title-abstract exclusion |
| 240 | Chou 2012 | 10.1155/2012/831282 | English | title-abstract exclusion |
| 241 | Huang 2013 | 10.1371/journal.pone.0080942 | English | title-abstract exclusion |
| 242 | Lin 2015 | 10.1016/j.vaccine.2015.04.077 | English | title-abstract exclusion |
| 243 | Zhang 2024 | 10.1128/jvi.01358-23 | English | title-abstract exclusion |
| 244 | Yan 2016 | 10.1016/j.vaccine.2016.06.058 | English | title-abstract exclusion |
| 245 | Gong 2014 | 10.1128/JVI.00200-14 | English | title-abstract exclusion |
| 246 | Lin 2014 | 10.1155/2014/670506 | English | title-abstract exclusion |
| 247 | Tian 2012 | 10.1371/journal.pone.0041381 | English | title-abstract exclusion |
| 248 | Li 2015 | 10.3390/v7072803 | English | title-abstract exclusion |
| 249 | Li 2014 | 10.1186/1743-422X-11-79 | English | title-abstract exclusion |
| 250 | Mao 2012 | 10.1007/s00705-011-1136-3 | English | title-abstract exclusion |
| 251 | Sun 2022 | 10.1080/14760584.2022.2011228 | English | title-abstract exclusion |
| 252 | Mao 2012 | 10.1371/journal.pone.0046043 | English | title-abstract exclusion |
| 253 | Zhang 2016 | 10.7883/yoken.JJID.2015.060 | English | title-abstract exclusion |
| 254 | Xia 2011 | 10.1016/j.diagmicrobio.2011.07.014 | English | title-abstract exclusion |
| 255 | Premanand 2012 | 10.1016/j.antiviral.2012.05.017 | English | title-abstract exclusion |
| 256 | Yang 2019 | 10.1128/JVI.00183-19 | English | title-abstract exclusion |
| 257 | Guo 2014 | 10.1016/j.antiviral.2013.11.002 | English | title-abstract exclusion |
| 258 | Fan 2019 | 10.1038/s41541-019-0108-6 | English | title-abstract exclusion |
| 259 | Qin 2014 | 10.1016/j.apsb.2014.06.006 | English | title-abstract exclusion |
| 260 | Shen 2016 | 10.1016/j.vaccine.2016.06.028 | English | title-abstract exclusion |
| 261 | Chou 2012 | 10.1371/journal.pone.0034834 | English | title-abstract exclusion |
| 262 | Taravilla 2019 | 10.3201/eid2501.181089 | English | title-abstract exclusion |
| 263 | Fu 2017 | 10.1016/j.vaccine.2017.06.002 | English | title-abstract exclusion |
| 264 | Foo 2007 | 10.1016/j.micinf.2007.06.002 | English | title-abstract exclusion |
| 265 | Ch'ng 2012 | 10.1186/1743-422X-9-155 | English | title-abstract exclusion |
| 266 | Chung 2010 | 10.1016/j.vaccine.2010.08.052 | English | title-abstract exclusion |
| 267 | Ji 2012 | 10.1186/1743-422X-9-248 | English | title-abstract exclusion |
| 268 | Liang 2012 | 10.1007/s10875-012-9690-3 | English | title-abstract exclusion |
| 269 | Caine 2013 | 10.1371/journal.pone.0059501 | English | title-abstract exclusion |
| 270 | Zhu 2023 | 10.1111/imm.13629 | English | title-abstract exclusion |
| 271 | Hu 2022 | 10.1016/j.vaccine.2022.06.021 | English | title-abstract exclusion |
| 272 | Chou 2013 | 10.1371/journal.pone.0079783 | English | title-abstract exclusion |
| 273 | Zhang 2019 | 10.1016/j.scitotenv.2018.10.349 | English | title-abstract exclusion |
| 274 | Liu 2014 | 10.1371/journal.pone.0106756 | English | title-abstract exclusion |
| 275 | Peng 2016 | 10.14715/cmb/2016.62.4.7 | English | title-abstract exclusion |
| 276 | Ji 2021 | 10.3389/fmicb.2021.658093 | English | title-abstract exclusion |
| 277 | Zhou 2016 | 10.1186/s12879-016-1463-9 | English | title-abstract exclusion |
| 278 | Bek 2010 | 10.2217/FVL.10.22 | English | title-abstract exclusion |
| 279 | Saeed 2015 | 10.7774/cevr.2015.4.1.88 | English | title-abstract exclusion |
| 280 | Yu 2013 | 10.1007/s00705-012-1589-z | English | title-abstract exclusion |
| 281 | Xu 2019 | 10.1007/s10529-019-02695-1 | English | title-abstract exclusion |
| 282 | Qian 2021 | 10.1080/22221751.2021.1906755 | English | title-abstract exclusion |
| 283 | Lin 2013 | 10.4161/hv.25639 | English | title-abstract exclusion |
| 284 | Zhang 2020 | 10.1016/j.ijid.2020.06.008 | English | title-abstract exclusion |
| 285 | Wang 2017 | 10.1038/s41598-017-09196-z | English | title-abstract exclusion |
| 286 | Zhang 2014 | 10.1097/INF.0000000000000194 | English | title-abstract exclusion |
| 287 | Zhang 2024 | 10.1002/jbt.23620 | English | title-abstract exclusion |
| 288 | Kiener 2013 | 10.1586/ERV.13.18 | English | title-abstract exclusion |
| 289 | Zhu 2017 | <https://www.europeanreview.org/article/13438> | English | title-abstract exclusion |
| 290 | Adewole 2024 | 10.1016/j.apm.2023.10.002 | English | title-abstract exclusion |
| 291 | Wu 2021 | 10.1016/j.lanwpc.2021.100092 | English | title-abstract exclusion |
| 292 | Sun 2018 | 10.1155/2018/2751457 | English | title-abstract exclusion |
| 293 | Pathinayake 2018 | 10.1007/s00705-018-3837-3 | English | title-abstract exclusion |
| 294 | Caine 2016 | 10.1128/JVI.01370-16 | English | title-abstract exclusion |
| 295 | Yan 2020 | 10.1097/MD.0000000000020473 | English | title-abstract exclusion |
| 296 | Chan 2017 | 10.12809/hkmj166098 | English | title-abstract exclusion |
| 297 | Solomon 2010 | 10.1016/S1473-3099(10)70194-8 | English | title-abstract exclusion |
| 298 | Hayes 2020 | 10.1002/jmv.25659 | English | title-abstract exclusion |
| 299 | Zhou 2018 | 10.1016/j.antiviral.2018.02.016 | English | title-abstract exclusion |
| 300 | Esposito 2018 | 10.1007/s10096-018-3206-x | English | title-abstract exclusion |
| 301 | Wang 2023 | 10.3389/fphar.2023.1164784 | English | title-abstract exclusion |
| 302 | Yao 2012 | 10.1371/journal.pone.0051996 | English | title-abstract exclusion |
| 303 | Chen 2014 | 10.1007/s40242-014-4133-8 | English | title-abstract exclusion |
| 304 | Ku 2013 | 10.1371/journal.pone.0057601 | English | title-abstract exclusion |
| 305 | Yang 2019 | <https://e-century.us/files/ijcem/12/11/ijcem0098896.pdf> | English | title-abstract exclusion |
| 306 | Xu 2016 | 10.7555/JBR.30.20140157 | English | title-abstract exclusion |
| 307 | Taniuchi 2016 | 10.1016/j.vaccine.2016.04.080 | English | title-abstract exclusion |
| 308 | Meng 2011 | 10.1371/journal.pone.0021757 | English | title-abstract exclusion |
| 309 | Gao 2012 | 10.1186/1743-422X-9-26 | English | title-abstract exclusion |
| 310 | Shen 2016 | 10.1016/j.vaccine.2016.08.033 | English | title-abstract exclusion |
| 311 | Chen 2018 | 10.1016/j.intimp.2018.04.050 | English | title-abstract exclusion |
| 312 | Fujii 2013 | 10.1073/pnas.1217563110 | English | title-abstract exclusion |
| 313 | Saeed 2015 | 10.1080/21645515.2015.1052918 | English | title-abstract exclusion |
| 314 | Zander 2014 | 10.5694/mja14.00014 | English | title-abstract exclusion |
| 315 | You 2023 | 10.1016/j.virusres.2023.199240 | English | title-abstract exclusion |
| 316 | Kim 2020 | 10.3390/nano10122342 | English | title-abstract exclusion |
| 317 | Liao 2020 | 10.3390/pathogens9020121 | English | title-abstract exclusion |
| 318 | Chadsuthi 2018 | 10.1155/2018/5168931 | English | title-abstract exclusion |
| 319 | Du 2019 | 10.1080/21645515.2019.1565266 | English | title-abstract exclusion |
| 320 | Cai 2013 | 10.1016/j.vaccine.2013.03.007 | English | title-abstract exclusion |
| 321 | Hooi 2020 | 10.1038/s41374-020-0456-x | English | title-abstract exclusion |
| 322 | Sun 2022 | 10.1080/22221751.2022.2093132 | English | title-abstract exclusion |
| 323 | Zhang 2017 | 10.1128/JVI.00333-17 | English | title-abstract exclusion |
| 324 | Wu 2007 | 10.1128/JVI.00372-07 | English | title-abstract exclusion |
| 325 | Premanand 2013 | 10.1371/journal.pone.0055536 | English | title-abstract exclusion |
| 326 | Shen 2023 | 10.46234/ccdcw2023.180 | English | title-abstract exclusion |
| 327 | Maimaiti 2022 | 10.3390/vaccines10122054 | English | title-abstract exclusion |
| 328 | Lugo 2016 | 10.1097/MOP.0000000000000303 | English | title-abstract exclusion |
| 329 | Lin 2018 | 10.1038/s41598-018-28281-5 | English | title-abstract exclusion |
| 330 | Deng 2015 | 10.1007/s00253-015-6652-8 | English | title-abstract exclusion |
| 331 | Liang 2018 | 10.3967/bes2018.045 | English | title-abstract exclusion |
| 332 | Khong 2011 | 10.1128/JVI.01779-10 | English | title-abstract exclusion |
| 333 | Liu 2016 | 10.3390/ijms17091419 | English | title-abstract exclusion |
| 334 | Lin 2015 | 10.1007/s00253-015-6588-z | English | title-abstract exclusion |
| 335 | Liu 2012 | 10.1016/j.vaccine.2012.08.071 | English | title-abstract exclusion |
| 336 | vanderSanden 2016 | 10.3201/eid2209.151579 | English | title-abstract exclusion |
| 337 | Ohka 2022 | 10.1242/bio.059469 | English | title-abstract exclusion |
| 338 | Li 2019 | 10.1186/s12865-019-0288-x | English | title-abstract exclusion |
| 339 | Zhao 2017 | 10.1371/journal.pone.0181182 | English | title-abstract exclusion |
| 340 | Lin 2019 | 10.1186/s12985-019-1203-z | English | title-abstract exclusion |
| 341 | Ku 2015 | 10.1128/JVI.02189-15 | English | title-abstract exclusion |
| 342 | Zhang 2022 | 10.1080/21645515.2022.2073751 | English | title-abstract exclusion |
| 343 | Jia 2017 | 10.1038/srep46402 | English | title-abstract exclusion |
| 344 | Ye 2014 | 10.1128/JVI.01848-13 | English | title-abstract exclusion |
| 345 | An 2023 | 10.1080/22221751.2022.2147022 | English | title-abstract exclusion |
| 346 | Chen 2019 | 10.1007/s00705-019-04418-3 | English | title-abstract exclusion |
| 347 | Kim 2020 | 10.1166/jnn.2020.17655 | English | title-abstract exclusion |
| 348 | Zhou 2023 | 10.1186/s40249-023-01145-5 | English | title-abstract exclusion |
| 349 | Gao 2021 | 10.1016/j.biopha.2021.112212 | English | title-abstract exclusion |
| 350 | Bible 2007 | 10.1002/rmv.538 | English | title-abstract exclusion |
| 351 | Tang 2019 | 10.1080/01621459.2019.1585250 | English | title-abstract exclusion |
| 352 | Xue 2015 | 10.1007/s12250-014-3555-2 | English | title-abstract exclusion |
| 353 | Zhang 2018 | 10.1016/j.vaccine.2018.09.069 | English | title-abstract exclusion |
| 354 | Zhu 2011 | 10.1016/j.ejps.2011.08.030 | English | title-abstract exclusion |
| 355 | Zhang 2019 | 10.1080/22221751.2019.1673135 | English | title-abstract exclusion |
| 356 | Ye 2016 | 10.1371/journal.ppat.1005454 | English | title-abstract exclusion |
| 357 | Ji 2018 | 10.1038/s41598-018-31616-x | English | title-abstract exclusion |
| 358 | Mizuta 2009 | 10.1016/j.vaccine.2009.03.060 | English | title-abstract exclusion |
| 359 | Deng 2010 | 10.1016/S1201-9712(10)60049-5 | English | title-abstract exclusion |
| 360 | Mwale 2021 | 10.3390/ijms22084146 | English | title-abstract exclusion |
| 361 | Deng 2014 | 10.1128/JVI.01207-14 | English | title-abstract exclusion |
| 362 | Lee 2023 | 10.3390/ani13122027 | English | title-abstract exclusion |
| 363 | Jiang 2020 | 10.4314/tjpr.v19i5.3 | English | title-abstract exclusion |
| 364 | Ye 2016 | 10.1007/s00253-016-7296-z | English | title-abstract exclusion |
| 365 | Huo 2021 | 10.1080/21645515.2021.1978792 | English | title-abstract exclusion |
| 366 | Gao 2024 | 10.1080/22221751.2024.2322671 | English | title-abstract exclusion |
| 367 | Kuo 2013 | 10.1186/1743-422X-10-28 | English | title-abstract exclusion |
| 368 | Wu 2019 | 10.1371/journal.pone.0210553 | English | title-abstract exclusion |
| 369 | Lin 2014 | 10.1371/journal.pone.0111496 | English | title-abstract exclusion |
| 370 | Wu 2015 | 10.1371/journal.pone.0136420 | English | title-abstract exclusion |
| 371 | Morales-HernÃ¡ndez 2022 | 10.3390/vaccines10091447 | English | title-abstract exclusion |
| 372 | Jiang 2021 | 10.3389/fimmu.2021.665197 | English | title-abstract exclusion |
| 373 | Xu 2015 | 10.1371/journal.pone.0119173 | English | title-abstract exclusion |
| 374 | Zhu 2022 | 10.1186/s12985-022-01939-3 | English | title-abstract exclusion |
| 375 | Qiao 2018 | 10.1021/acs.nanolett.8b00478 | English | title-abstract exclusion |
| 376 | Zhang 2016 | 10.1016/j.antiviral.2016.02.011 | English | title-abstract exclusion |
| 377 | Li 2014 | 10.1099/vir.0.063560-0 | English | title-abstract exclusion |
| 378 | Mao 2012 | 10.1128/JVI.00902-12 | English | title-abstract exclusion |
| 379 | Yang 2016 | 10.1016/j.antiviral.2016.08.025 | English | title-abstract exclusion |
| 380 | Gong 2021 | 10.3390/vaccines9050476 | English | title-abstract exclusion |
| 381 | Lin 2022 | 10.1080/21645515.2022.2143176 | English | title-abstract exclusion |
| 382 | Cao 2013 | 10.1371/journal.pone.0064024 | English | title-abstract exclusion |
| 383 | Zhang 2024 | 10.1080/21645515.2024.2319967 | English | title-abstract exclusion |
| 384 | Sabanathan 2014 | 10.1136/jech-2014-203836 | English | title-abstract exclusion |
| 385 | Ren 2015 | 10.1128/JVI.01102-15 | English | title-abstract exclusion |
| 386 | Cao 2019 | 10.5114/ceji.2019.84009 | English | title-abstract exclusion |
| 387 | WHOProdDevVaccinesAdvisory 2019 | 10.1016/j.vaccine.2016.10.090 | English | title-abstract exclusion |
| 388 | Shao 2015 | 10.1371/journal.pone.0140515 | English | title-abstract exclusion |
| 389 | Meng 2014 | 10.1128/JVI.00289-14 | English | title-abstract exclusion |
| 390 | Zhao 2013 | 10.1007/s00253-013-5257-3 | English | title-abstract exclusion |
| 391 | Jin 2013 | 10.1016/j.jviromet.2012.11.014 | English | title-abstract exclusion |
| 392 | Itani 2023 | 10.3390/vaccines11101588 | English | title-abstract exclusion |
| 393 | Zhang 2023 | 10.3389/fmicb.2023.1172349 | English | title-abstract exclusion |
| 394 | Chen 2020 | 10.1016/j.virusres.2020.198067 | English | title-abstract exclusion |
| 395 | Molina 2020 | 10.1016/j.antiviral.2020.104850 | English | title-abstract exclusion |
| 396 | Zhang 2021 | 10.1016/j.vaccine.2021.07.095 | English | title-abstract exclusion |
| 397 | Lai 2016 | 10.3390/v8020032 | English | title-abstract exclusion |
| 398 | Zhou 2016 | 10.1016/j.antiviral.2016.06.004 | English | title-abstract exclusion |
| 399 | Tijsma 2014 | 10.1128/AAC.03328-14 | English | title-abstract exclusion |
| 400 | Cubillos 2008 | 10.1128/JVI.00401-08 | English | title-abstract exclusion |
| 401 | Summerfield 2022 | 10.3389/fvets.2022.1033276 | English | title-abstract exclusion |
| 402 | Wang 2021 | 10.1016/j.vaccine.2021.11.020 | English | title-abstract exclusion |
| 403 | Thirion-Romero 2023 | 10.3390/vaccines11121779 | English | title-abstract exclusion |
| 404 | Ma 2022 | 10.3390/vaccines10071010 | English | title-abstract exclusion |
| 405 | An 2014 | 10.4161/hv.27295 | English | title-abstract exclusion |
| 406 | DCVMNExecutiveCommGrp 2015 | 10.1016/j.vaccine.2015.02.065 | English | title-abstract exclusion |
| 407 | Lin 2009 | 10.1128/JVI.00434-09 | English | title-abstract exclusion |
| 408 | Sun 2016 | 10.1038/srep34299 | English | title-abstract exclusion |
| 409 | Feng 2016 | 10.12659/MSM.900380 | English | title-abstract exclusion |
| 410 | Tan 2007 | 10.1007/s00705-007-0941-1 | English | title-abstract exclusion |
| 411 | Cui 2021 | 10.1080/21645515.2020.1776547 | English | title-abstract exclusion |
| 412 | Hou 2015 | 10.1016/j.jviromet.2015.02.010 | English | title-abstract exclusion |
| 413 | Wu 2013 | 10.1371/journal.pone.0064116 | English | title-abstract exclusion |
| 414 | Wang 2024 | 10.1080/22221751.2024.2337665 | English | title-abstract exclusion |
| 415 | Pastoret 2005 | <https://pubmed.ncbi.nlm.nih.gov/15962481/> | English | title-abstract exclusion |
| 416 | Linn 2020 | 10.1080/21645515.2019.1695460 | English | title-abstract exclusion |
| 417 | Francis 2016 | 10.1186/s12887-016-0740-5 | English | title-abstract exclusion |
| 418 | Kadambari 2014 | 10.1111/1469-0691.12753 | English | title-abstract exclusion |
| 419 | Yao 2019 | 10.1016/j.vaccine.2019.07.046 | English | title-abstract exclusion |
| 420 | Qiu 2012 | 10.1186/1743-422X-9-285 | English | title-abstract exclusion |
| 421 | Zhou 2022 | 10.1186/s12916-022-02604-w | English | title-abstract exclusion |
| 422 | Knippenberg 2017 | 10.1071/MA17069 | English | title-abstract exclusion |
| 423 | Zhou 2016 | 10.1039/c6cc02595a | English | title-abstract exclusion |
| 424 | Dai 2019 | 10.1016/j.antiviral.2019.02.016 | English | title-abstract exclusion |
| 425 | Sun 2021 | 10.1080/21645515.2021.1956227 | English | title-abstract exclusion |
| 426 | Riddle 2016 | 10.1016/j.vaccine.2016.03.077 | English | title-abstract exclusion |
| 427 | Wang 2013 | 10.1073/pnas.1300233110 | English | title-abstract exclusion |
| 428 | Zhao 2015 | 10.1111/jam.12922 | English | title-abstract exclusion |
| 429 | Tang 2016 | 10.1038/srep25741 | English | title-abstract exclusion |
| 430 | Pan 2015 | 10.1016/j.vaccine.2015.09.081 | English | title-abstract exclusion |
| 431 | WILEY 1992 | 10.1128/JVI.66.10.5744-5751.1992 | English | title-abstract exclusion |
| 432 | Omori 2012 | 10.1371/journal.pone.0050751 | English | title-abstract exclusion |
| 433 | Li 2017 | 10.1016/j.antiviral.2017.06.008 | English | title-abstract exclusion |
| 434 | Huang 2021 | 10.1016/j.coviro.2021.10.006 | English | title-abstract exclusion |
| 435 | Wang 2019 | 10.1039/c9bm00098d | English | title-abstract exclusion |
| 436 | Mahmud 2014 | 10.1200/JCO.2013.52.4645 | English | title-abstract exclusion |
| 437 | [Anonymous] 2021 | 10.1080/21645515.2021.1940535 | English | title-abstract exclusion |
| 438 | Pons-Salort 2018 | 10.1126/science.aat6777 | English | title-abstract exclusion |
| 439 | Elmgren 2013 | 10.1016/j.vaccine.2012.10.117 | English | title-abstract exclusion |
| 440 | Liu 2022 | 10.3389/fimmu.2022.814365 | English | title-abstract exclusion |
| 441 | Danilova 2023 | 10.25789/YMJ.2023.84.18 | English | title-abstract exclusion |
| 442 | Shimizu 2016 | 10.1016/j.vaccine.2014.11.015 | English | title-abstract exclusion |
| 443 | Li 2022 | 10.1080/22221751.2022.2119166 | English | title-abstract exclusion |
| 444 | Ho 2008 | 10.1128/IAI.00795-07 | English | title-abstract exclusion |
| 445 | Win 2021 | 10.1371/journal.pone.0258765 | English | title-abstract exclusion |
| 446 | vanderSanden 2016 | 10.1128/JVI.01464-15 | English | title-abstract exclusion |
| 447 | Gao 2021 | 10.1016/j.virol.2020.07.003 | English | title-abstract exclusion |
| 448 | Langford 2003 | 10.1076/ocii.11.3.197.17352 | English | title-abstract exclusion |
| 449 | Xu 2015 | 10.1586/14760584.2015.1012503 | English | title-abstract exclusion |
| 450 | Deng 2020 | 10.1016/j.seizure.2020.03.005 | English | title-abstract exclusion |
| 451 | Chen 2005 | 10.1016/j.antiviral.2005.02.004 | English | title-abstract exclusion |
| 452 | Zheng 2023 | 10.3390/vaccines11101606 | English | title-abstract exclusion |
| 453 | Drolet 2015 | 10.1016/S1473-3099(14)71073-4 | English | title-abstract exclusion |
| 454 | Shi 2013 | 10.1016/j.vaccine.2013.02.051 | English | title-abstract exclusion |
| 455 | Du 2023 | 10.1080/22221751.2022.2149352 | English | title-abstract exclusion |
| 456 | Lerner 2021 | 10.1093/cid/ciaa1432 | English | title-abstract exclusion |
| 457 | Zurynski 2016 | <https://pubmed.ncbi.nlm.nih.gov/28278415/> | English | title-abstract exclusion |
| 458 | Wozniak-Kosek 2015 | 10.1007/5584_2014_3110.1007/978-3-319-10018-0 | English | title-abstract exclusion |
| 459 | Schmidt 2012 | 10.1002/eji.201242393 | English | title-abstract exclusion |
| 460 | Jin 2021 | 10.1038/s41392-021-00481-y | English | title-abstract exclusion |
| 461 | Zhang 2012 | 10.1016/j.vaccine.2012.01.061 | English | title-abstract exclusion |
| 462 | Hooi 2015 | <http://www.scopus.com/inward/record.url?scp=84953207623&partnerID=8YFLogxK> | English | title-abstract exclusion |
| 463 | Ruggiero 2022 | 10.3389/fimmu.2022.956825 | English | title-abstract exclusion |
| 464 | Lu 2019 | 10.3390/ijerph16020168 | English | title-abstract exclusion |
| 465 | Kiulia 2021 | 10.1007/s12560-020-09455-9 | English | title-abstract exclusion |
| 466 | Raha 2005 | 10.1007/s00253-004-1851-8 | English | title-abstract exclusion |
| 467 | Bao 2020 | 10.1111/jpc.15140 | English | title-abstract exclusion |
| 468 | Huang 2022 | 10.1016/j.eng.2022.01.001 | English | title-abstract exclusion |
| 469 | Liang 2014 | 10.1038/emi.2014.66 | English | title-abstract exclusion |
| 470 | Chen 2022 | 10.3390/pathogens11111371 | English | title-abstract exclusion |
| 471 | Xue 2014 | 10.1016/j.virusres.2014.01.027 | English | title-abstract exclusion |
| 472 | Quach 2018 | 10.1016/j.actbio.2018.08.011 | English | title-abstract exclusion |
| 473 | Zhang 2022 | 10.1038/s41467-022-35575-w | English | title-abstract exclusion |
| 474 | Zhu 2018 | 10.1126/sciadv.aat7459 | English | title-abstract exclusion |
| 475 | Dong 2014 | 10.1007/s00253-014-5639-1 | English | title-abstract exclusion |
| 476 | Chang 2008 | 10.1016/S0929-6646(08)60175-8 | English | title-abstract exclusion |
| 477 | Anasir 2021 | 10.1186/s12929-021-00708-8 | English | title-abstract exclusion |
| 478 | He 2020 | 10.1016/j.chom.2020.01.003 | English | title-abstract exclusion |
| 479 | Park 2017 | 10.1038/s41541-017-0033-5 | English | title-abstract exclusion |
| 480 | Shi 2020 | 10.1093/nar/gkz1170 | English | title-abstract exclusion |
| 481 | HPVEduStudyGrp 2023 | 10.1016/j.ypmed.2023.107542 | English | title-abstract exclusion |
| 482 | Lei 2019 | 10.1002/jmv.25554 | English | title-abstract exclusion |
| 483 | Anders 2013 | 10.1186/1471-2458-13-937 | English | title-abstract exclusion |
| 484 | Chen 2023 | 10.1016/j.lanwpc.2023.100811 | English | title-abstract exclusion |
| 485 | Real-Hohn 2021 | 10.3390/v13091784 | English | title-abstract exclusion |
| 486 | Riedmann 2013 | 10.4161/hv.25792 | English | title-abstract exclusion |
| 487 | Chen 2022 | 10.1128/jvi.00143-22 | English | title-abstract exclusion |
| 488 | Mao 2021 | 10.1038/s41392-021-00621-4 | English | title-abstract exclusion |
| 489 | Shaik 2023 | 10.5455/jcmr.2023.14.02.06 | English | title-abstract exclusion |
| 490 | Deshpande 2014 | 10.1093/infdis/jiu204 | English | title-abstract exclusion |
| 491 | Qiao 2022 | 10.1016/j.antiviral.2021.105231 | English | title-abstract exclusion |
| 492 | Zhang 2016 | 10.1002/rmv.1867 | English | title-abstract exclusion |
| 493 | Sattler 2024 | 10.1172/jci.insight.166833 | English | title-abstract exclusion |
| 494 | Song 2017 | 10.1038/s41598-017-05618-0 | English | title-abstract exclusion |
| 495 | Du 2019 | 10.1021/acsnano.9b04071 | English | title-abstract exclusion |
| 496 | Wang 2018 | 10.1016/j.vetmic.2017.12.020 | English | title-abstract exclusion |
| 497 | Eshima 2012 | 10.1371/journal.pone.0042261 | English | title-abstract exclusion |
| 498 | Feng 2014 | 10.1371/journal.pone.0095927 | English | title-abstract exclusion |
| 499 | Li 2023 | 10.1007/s11356-022-22711-8 | English | title-abstract exclusion |
| 500 | Yu 2018 | 10.1016/j.envres.2018.06.047 | English | title-abstract exclusion |
| 501 | Mandary 2019 | 10.3390/ijms20184657 | English | title-abstract exclusion |
| 502 | Lacasta 2021 | 10.4049/jimmunol.2000442 | English | title-abstract exclusion |
| 503 | Chan 2011 | 10.1371/journal.pone.0028885 | English | title-abstract exclusion |
| 504 | Adeyemi 2017 | 10.1128/JVI.01586-16 | English | title-abstract exclusion |
| 505 | Barnard 2006 | 10.2174/138161206776361129 | English | title-abstract exclusion |
| 506 | Sandoni 2022 | 10.3390/pathogens11010060 | English | title-abstract exclusion |
| 507 | Song 2017 | 10.1016/j.antiviral.2017.07.003 | English | title-abstract exclusion |
| 508 | Reichler 1997 | 10.1093/infdis/175.Supplement_1.S62 | English | title-abstract exclusion |
| 509 | Netto 2022 | 10.1016/S2352-3018(22)00033-9 | English | title-abstract exclusion |
| 510 | Mizuta 2013 | 10.1111/1348-0421.12041 | English | title-abstract exclusion |
| 511 | Wu 2023 | 10.1016/j.apsb.2023.03.015 | English | title-abstract exclusion |
| 512 | Kim 2014 | 10.1002/jmv.23763 | English | title-abstract exclusion |
| 513 | Mrochen 2021 | 10.3389/fimmu.2021.642802 | English | title-abstract exclusion |
| 514 | Ambrose 2022 | 10.3390/molecules27061773 | English | title-abstract exclusion |
| 515 | Xie 2022 | 10.3389/fmed.2022.822796 | English | title-abstract exclusion |
| 516 | Wen 2019 | 10.3390/vaccines7030111 | English | title-abstract exclusion |
| 517 | Zhou 2024 | 10.1128/spectrum.04007-23 | English | title-abstract exclusion |
| 518 | Chen 2020 | 10.1371/journal.pntd.0008910 | English | title-abstract exclusion |
| 519 | Ali 2021 | 10.1002/ptr.7039 | English | title-abstract exclusion |
| 520 | RodrÃ­guez-HernÃ¡ndez 2023 | 10.3390/v15020411 | English | title-abstract exclusion |
| 521 | IntMaternalPediatAdolescentAID 2021 | 10.1093/infdis/jiab229 | English | title-abstract exclusion |
| 522 | Goh 2020 | 10.3390/molecules25122796 | English | title-abstract exclusion |
| 523 | GBD2019Meningitis&Antimicro 2023 | 10.1016/S1474-4422(23)00195-3 | English | title-abstract exclusion |
| 524 | GBD2019Under-5MortalityCollabo 2021 | 10.1016/S0140-6736(21)01207-1 | English | title-abstract exclusion |
| 525 | GBD2019RiskFactors 2020 | 10.1016/S0140-6736(20)30752-2 | English | title-abstract exclusion |
| 526 | Chu 2023 | https://doi.org/10.1016/j.ijid.2023.04.099 | English | title-abstract exclusion |
| 527 | Wang 2018 | https://doi.org/10.1016/j.jviromet.2018.05.005 | English | title-abstract exclusion |
| 528 | Li 2024 | https://doi.org/10.1016/j.bsheal.2023.11.001 | English | title-abstract exclusion |
| 529 | Tang 2022 | https://doi.org/10.1016/j.cellin.2022.100016 | English | title-abstract exclusion |
| 530 | Wang 2022 | https://doi.org/10.1016/j.apsb.2021.08.017 | English | title-abstract exclusion |
| 531 | Tan 2016 | https://doi.org/10.1016/j.antiviral.2016.04.015 | English | title-abstract exclusion |
| 532 | Moraga-Llop 2017 | https://doi.org/10.1016/j.vacune.2016.11.001 | English | title-abstract exclusion |
| 533 | Xin 2010 | https://doi.org/10.1016/j.provac.2010.03.012 | English | title-abstract exclusion |
| 534 | Guo 2014 | https://doi.org/10.1016/j.antiviral.2013.11.002 | English | title-abstract exclusion |
| 535 | Huang 2014 | https://doi.org/10.1016/j.coviro.2014.03.007 | English | title-abstract exclusion |
| 536 | Wang 2013 | https://doi.org/10.1016/j.bbrc.2012.11.035 | English | title-abstract exclusion |
| 537 | Whitlock 2010 | https://doi.org/10.1016/j.provac.2010.03.013 | English | title-abstract exclusion |
| 538 | Cao 2013 | https://doi.org/10.2478/v10136-012-0032-9 | English | title-abstract exclusion |
| 539 | Jiang 2015 | https://doi.org/10.1016/j.vaccine.2015.10.104 | English | title-abstract exclusion |
| 540 | Li 2017 | https://doi.org/10.1016/j.vaccine.2017.05.060 | English | title-abstract exclusion |
| 541 | Qin 2014 | https://doi.org/10.1016/j.apsb.2014.06.006 | English | title-abstract exclusion |
| 542 | Chiu 2006 | https://doi.org/10.1016/j.micinf.2006.01.021 | English | title-abstract exclusion |
| 543 | Zhang 2015 | https://doi.org/10.1016/j.vaccine.2015.03.034 | English | title-abstract exclusion |
| 544 | Deng 2010 | https://doi.org/10.1016/S1201-9712(10)60049-5 | English | title-abstract exclusion |
| 545 | Li 2013 | https://doi.org/10.1016/j.vaccine.2013.05.019 | English | title-abstract exclusion |
| 546 | Muir 2005 | https://doi.org/10.1383/medc.33.5.140.64955 | English | title-abstract exclusion |
| 547 | Costa 2019 | https://doi.org/10.1016/j.wasman.2019.04.047 | English | title-abstract exclusion |
| 548 | Zhang 2016 | https://doi.org/10.1016/j.antiviral.2016.02.011 | English | title-abstract exclusion |
| 549 | Han 2022 | 10.1080/21645515.2022.2049168 | English | title-abstract exclusion |
| 550 | Lalani 2021 | 10.1016/j.peptides.2020.170443 | English | title-abstract exclusion |
| 551 | Lalani 2020 | 10.3390/v12020184 | English | title-abstract exclusion |
| 552 | Lai 2023 | 10.1007/s00705-023-05882-8 | English | title-abstract exclusion |
| 553 | Swain 2022 | 10.1002/rmv.2322 | English | title-abstract exclusion |
| 554 | Gonzalez 2019 | 10.3390/ijms20205201 | English | title-abstract exclusion |
| 555 | Ang 2021 | 10.1080/22221751.2021.1906754 | English | title-abstract exclusion |
| 556 | Shih 2018 | 10.3390/v10120674 | English | title-abstract exclusion |
| 557 | Shi 2023 | 10.1007/s00705-022-05642-0 | English | title-abstract exclusion |
| 558 | Wang 2024 | 10.1016/j.jare.2023.03.007 | English | title-abstract exclusion |
| 559 | Lin 2014 | 10.1186/1423-0127-21-18 | English | title-abstract exclusion |
| 560 | Sarma 2013 | 10.4103/0378-6323.107631 | English | title-abstract exclusion |
| 561 | Pathinayake 2015 | 10.3390/v7122961 | English | title-abstract exclusion |
| 562 | Swain 2022 | 10.3390/v14102190 | English | title-abstract exclusion |
| 563 | Lim 2019 | 10.1177/2515135519888998 | English | title-abstract exclusion |
| 564 | Wang 2014 | 10.1186/1423-0127-21-31 | English | title-abstract exclusion |
| 565 | Lei 2021 | 10.12659/AOT.924461 | English | title-abstract exclusion |
| 566 | Yu 2023 | 10.3390/v15040932 | English | title-abstract exclusion |
| 567 | Tee 2021 | 10.1080/14787210.2021.1851194 | English | title-abstract exclusion |
| 568 | Tan 2021 | 10.1038/s41598-021-89271-8 | English | title-abstract exclusion |
| 569 | Wang 2019 | 10.1007/s12250-018-0071-9 | English | title-abstract exclusion |
| 570 | Zeng 2021 | 10.1016/j.jinf.2020.12.020 | English | title-abstract exclusion |
| 571 | Lim 2020 | 10.1080/17460441.2019.1659241 | English | title-abstract exclusion |
| 572 | Yang 2015 | 10.1097/INF.0000000000000900 | English | title-abstract exclusion |
| 573 | Lin 2023 | 10.1186/s12929-023-00987-3 | English | title-abstract exclusion |
| 574 | Liu 2018 | 10.1016/j.vaccine.2017.02.042 | English | title-abstract exclusion |
| 575 | Chen 2022 | 10.1007/s11427-021-2095-0 | English | title-abstract exclusion |
| 576 | Wang 2018 | 10.1038/s41426-017-0011-z | English | title-abstract exclusion |
| 577 | Du 2021 | 10.3390/ijerph18010292 | English | title-abstract exclusion |
| 578 | Yang 2024 | 10.3390/v16040573 | English | title-abstract exclusion |
| 579 | Liu 2021 | 10.1186/s12879-021-06157-w | English | title-abstract exclusion |
| 580 | Wang 2014 | 10.1016/j.antiviral.2013.12.008 | English | title-abstract exclusion |
| 581 | Cao 2020 | 10.1007/s12250-020-00226-1 | English | title-abstract exclusion |
| 582 | Lalani 2021 | 10.3390/ijms22168757 | English | title-abstract exclusion |
| 583 | Masomian 2021 | 10.3390/molecules26216576 | English | title-abstract exclusion |
| 584 | Zhao 2020 | 10.1002/rmv.2087 | English | title-abstract exclusion |
| 585 | Zhang 2022 | 10.1002/jmv.27268 | English | title-abstract exclusion |
| 586 | Ke 2019 | 10.1128/JVI.01066-18 | English | title-abstract exclusion |
| 587 | Somasundaram 2016 | 10.1016/j.ymeth.2015.09.023 | English | title-abstract exclusion |
| 588 | Abd-Aziz 2024 | 10.1016/j.virol.2023.109941 | English | title-abstract exclusion |
| 589 | Lee 2009 | 10.1097/INF.0b013e3181a41d63 | English | title-abstract exclusion |
| 590 | Xu 2010 | 10.1016/j.vaccine.2010.03.003 | English | title-abstract exclusion |
| 591 | Wang 2023 | 10.1080/21645515.2022.2164140 | English | title-abstract exclusion |
| 592 | Freeman 2022 | 10.1128/mbio.00457-22 | English | title-abstract exclusion |
| 593 | Liao 2018 | 10.1017/S0950268818000018 | English | title-abstract exclusion |
| 594 | Poh 2021 | 10.1016/bs.vh.2021.06.008 | English | title-abstract exclusion |
| 595 | Shih 2011 | 10.1128/JVI.05063-11 | English | title-abstract exclusion |
| 596 | Xing 2014 | 10.1016/S1473-3099(13)70342-6 | English | title-abstract exclusion |
| 597 | Shang 2013 | 10.1016/j.antiviral.2012.12.005 | English | title-abstract exclusion |
| 598 | Lalani 2021 | 10.1016/j.lfs.2021.120097 | English | title-abstract exclusion |
| 599 | Koh 2016 | 10.1097/INF.0000000000001242 | English | title-abstract exclusion |
| 600 | Hu 2023 | 10.1016/j.virusres.2023.199195 | English | title-abstract exclusion |
| 601 | Xu 2018 | 10.4149/av_2018_106 | English | title-abstract exclusion |
| 602 | Zhao 2017 | 10.3389/fcimb.2017.00171 | English | title-abstract exclusion |
| 603 | Chang 2012 | 10.1016/j.vaccine.2011.11.087 | English | title-abstract exclusion |
| 604 | Zhu 2016 | 10.1016/j.jconrel.2016.10.019 | English | title-abstract exclusion |
| 605 | Li 2021 | 10.1080/21645515.2020.1778409 | English | title-abstract exclusion |
| 606 | Kuo 2020 | 10.1371/journal.pntd.0008124 | English | title-abstract exclusion |
| 607 | McMinn 2012 | 10.1016/j.coviro.2012.02.009 | English | title-abstract exclusion |
| 608 | Dai 2017 | 10.1016/j.antiviral.2017.07.001 | English | title-abstract exclusion |
| 609 | Wang 2019 | 10.1016/j.jiac.2019.07.010 | English | title-abstract exclusion |
| 610 | Chen 2024 | 10.1016/j.epidem.2024.100754 | English | title-abstract exclusion |
| 611 | Zhang 2020 | 10.1016/j.jcv.2020.104582 | English | title-abstract exclusion |
| 612 | Lyu 2015 | 10.1074/jbc.M114.624536 | English | title-abstract exclusion |
| 613 | Yang 2016 | 10.1016/j.vaccine.2016.10.018 | English | title-abstract exclusion |
| 614 | Rajamoorthy 2023 | 10.1371/journal.pone.0286924 | English | title-abstract exclusion |
| 615 | Wongsa 2019 | 10.1016/j.micpath.2019.05.008 | English | title-abstract exclusion |
| 616 | Zhao 2016 | 10.1186/s12879-016-2008-y | English | title-abstract exclusion |
| 617 | Zhang 2016 | 10.1089/vim.2015.0110 | English | title-abstract exclusion |
| 618 | Qi 2019 | 10.1371/journal.pone.0225569 | English | title-abstract exclusion |
| 619 | Supasorn 2020 | 10.1007/s13365-019-00824-0 | English | title-abstract exclusion |
| 620 | Wang 2016 | 10.3390/ijerph14010033 | English | title-abstract exclusion |
| 621 | Shingler 2015 | 10.1128/JVI.03098-14 | English | title-abstract exclusion |
| 622 | Chia 2014 | 10.1371/journal.pntd.0003044 | English | title-abstract exclusion |
| 623 | Lalani 2020 | 10.1186/s12906-020-2880-2 | English | title-abstract exclusion |
| 624 | Shang 2013 | 10.1016/j.antiviral.2012.12.010 | English | title-abstract exclusion |
| 625 | KatturVenkatachalam 2014 | 10.1186/1743-422X-11-99 | English | title-abstract exclusion |
| 626 | Hung 2011 | 10.1093/infdis/jir174 | English | title-abstract exclusion |
| 627 | Tian 2017 | 10.1371/journal.pone.0176604 | English | title-abstract exclusion |
| 628 | Hu 2021 | 10.1016/j.virusres.2021.198502 | English | title-abstract exclusion |
| 629 | Cao 2019 | 10.1016/j.micpath.2019.103568 | English | title-abstract exclusion |
| 630 | Salmons 2018 | 10.1016/j.vaccine.2018.09.062 | English | title-abstract exclusion |
| 631 | Yuan 2015 | 10.1007/s12026-015-8637-1 | English | title-abstract exclusion |
| 632 | Feng 2020 | 10.1038/s41374-019-0351-5 | English | title-abstract exclusion |
| 633 | Wu 2021 | 10.1080/22221751.2021.1934558 | English | title-abstract exclusion |
| 634 | Lv 2014 | 10.1016/j.antiviral.2014.06.004 | English | title-abstract exclusion |
| 635 | Meng 2021 | 10.1128/JVI.01055-21 | English | title-abstract exclusion |
| 636 | Lin 2020 | 10.3389/fimmu.2020.561758 | English | title-abstract exclusion |
| 637 | Xu 2017 | 10.1038/s41467-017-00477-9 | English | title-abstract exclusion |
| 638 | Yu 2013 | 10.1007/s00705-012-1589-z | English | title-abstract exclusion |
| 639 | Wen 2013 | 10.1159/000348504 | English | title-abstract exclusion |
| 640 | Kim 2021 | 10.1089/vim.2020.0073 | English | title-abstract exclusion |
| 641 | Zhang 2013 | 10.1007/s11262-013-0955-6 | English | title-abstract exclusion |
| 642 | Zhang 2017 | 10.3390/ijerph14030319 | English | title-abstract exclusion |
| 643 | Ang 2021 | 10.47665/tb.38.3.063 | English | title-abstract exclusion |
| 644 | Li 2013 | 10.1371/journal.pone.0080515 | English | title-abstract exclusion |
| 645 | Liu 2016 | 10.1016/j.antiviral.2016.02.008 | English | title-abstract exclusion |
| 646 | Cheng 2022 | 10.1186/s12929-022-00794-2 | English | title-abstract exclusion |
| 647 | Hankaniemi 2017 | 10.1016/j.vaccine.2017.05.057 | English | title-abstract exclusion |
| 648 | Yang 2020 | 10.1007/s00284-020-02026-y | English | title-abstract exclusion |
| 649 | Lim 2019 | 10.1016/j.jviromet.2019.05.005 | English | title-abstract exclusion |
| 650 | Zhu 2012 | 10.1371/journal.pone.0037206 | English | title-abstract exclusion |
| 651 | Huang 2019 | 10.1016/j.vaccine.2019.02.023 | English | title-abstract exclusion |
| 652 | Li 2020 | 10.1080/21645515.2019.1691404 | English | title-abstract exclusion |
| 653 | Su 2017 | 10.1016/j.meegid.2017.06.004 | English | title-abstract exclusion |
| 654 | Cheng 2022 | 10.3390/v14102306 | English | title-abstract exclusion |
| 655 | Zhao 2022 | 10.1002/jmv.27796 | English | title-abstract exclusion |
| 656 | Yao 2012 | 10.1371/journal.pone.0051996 | English | title-abstract exclusion |
| 657 | Zhang 2018 | 10.1038/s41426-017-0005-x | English | title-abstract exclusion |
| 658 | Lin 2012 | 10.1016/j.vaccine.2011.12.081 | English | title-abstract exclusion |
| 659 | Tu 2015 | 10.1080/21645515.2015.1016667 | English | title-abstract exclusion |
| 660 | Hou 2015 | 10.1016/j.virusres.2015.05.011 | English | title-abstract exclusion |
| 661 | Sun 2014 | 10.4161/hv.29823 | English | title-abstract exclusion |
| 662 | Liang 2019 | 10.1016/j.bbrc.2019.02.014 | English | title-abstract exclusion |
| 663 | Meng 2012 | 10.1186/1743-422X-9-238 | English | title-abstract exclusion |
| 664 | Xu 2014 | 10.3390/v6072778 | English | title-abstract exclusion |
| 665 | Li 2019 | 10.1186/s12865-019-0288-x | English | title-abstract exclusion |
| 666 | Han 2014 | 10.4161/hv.29123 | English | title-abstract exclusion |
| 667 | Wu 2010 | 10.1016/j.ijid.2010.07.006 | English | title-abstract exclusion |
| 668 | Amrun 2020 | 10.1038/s41598-020-60761-5 | English | title-abstract exclusion |
| 669 | Yao 2018 | 10.1080/21645515.2018.1426420 | English | title-abstract exclusion |
| 670 | Yang 2012 | 10.3390/molecules170910370 | English | title-abstract exclusion |
| 671 | Falah 2012 | 10.1093/jac/dks304 | English | title-abstract exclusion |
| 672 | Zhong 2019 | 10.1080/21691401.2019.1640716 | English | title-abstract exclusion |
| 673 | Meng 2012 | 10.4161/hv.19521 | English | title-abstract exclusion |
| 674 | Cui 2011 | 10.1016/j.jmb.2011.03.007 | English | title-abstract exclusion |
| 675 | Li 2013 | 10.1186/1471-2334-13-322 | English | title-abstract exclusion |
| 676 | Pallansch 2013 | 10.1016/S0140-6736(13)60286-X | English | title-abstract exclusion |
| 677 | DeCoster 2021 | 10.1016/S0140-6736(20)32541-1 | English | title-abstract exclusion |
| 678 | Zhang 2016 | 10.1177/0300060515604981 | English | title-abstract exclusion |
| 679 | Yang 2011 | 10.1186/1743-422X-8-306 | English | title-abstract exclusion |
| 680 | LaddEffio 2016 | 10.1016/j.vaccine.2016.01.035 | English | title-abstract exclusion |
| 681 | Chen 2017 | 10.1080/19420862.2016.1267086 | English | title-abstract exclusion |
| 682 | Yee 2016 | 10.1186/s12985-016-0645-9 | English | title-abstract exclusion |
| 683 | Zeng 2012 | 10.1016/j.jcv.2011.12.025 | English | title-abstract exclusion |
| 684 | Ang 2016 | 10.1186/s12985-016-0567-6 | English | title-abstract exclusion |
| 685 | Huang 2016 | 10.1371/journal.pone.0148907 | English | title-abstract exclusion |
| 686 | Yeo 2015 | 10.1007/s12272-014-0390-9 | English | title-abstract exclusion |
| 687 | Chen 2018 | 10.1128/JVI.01257-17 | English | title-abstract exclusion |
| 688 | Song 2018 | 10.1007/s00705-017-3592-x | English | title-abstract exclusion |
| 689 | Kirk 2012 | 10.1016/j.vaccine.2012.09.030 | English | title-abstract exclusion |
| 690 | Cheng 2013 | 10.1016/j.vaccine.2013.03.015 | English | title-abstract exclusion |
| 691 | Lin 2015 | 10.1007/s12250-015-3573-8 | English | title-abstract exclusion |
| 692 | Hwang 2013 | 10.1002/jmv.23588 | English | title-abstract exclusion |
| 693 | Zhang 2014 | 10.1371/journal.pone.0100545 | English | title-abstract exclusion |
| 694 | Wei 2012 | 10.1371/journal.pone.0051957 | English | title-abstract exclusion |
| 695 | Chong 2012 | 10.1371/journal.pone.0049973 | English | title-abstract exclusion |
| 696 | Saeed 2015 | 10.1080/21645515.2015.1052918 | English | title-abstract exclusion |
| 697 | Wu 2016 | 10.1038/ncomms13150 | English | title-abstract exclusion |
| 698 | Lin 2013 | 10.1371/journal.pone.0057591 | English | title-abstract exclusion |
| 699 | Khong 2012 | 10.1128/JVI.06103-11 | English | title-abstract exclusion |
| 700 | Yang 2015 | 10.1016/j.bmcl.2015.06.097 | English | title-abstract exclusion |
| 701 | Dang 2014 | 10.1111/imm.12235 | English | title-abstract exclusion |
| 702 | Roberts 2019 | 10.1038/s41598-019-41662-8 | English | title-abstract exclusion |
| 703 | Liao 2014 | 10.1128/JVI.00692-14 | English | title-abstract exclusion |
| 704 | Tan 2013 | 10.4049/jimmunol.1301439 | English | title-abstract exclusion |
| 705 | Wang 2017 | 10.1016/j.virol.2016.10.031 | English | title-abstract exclusion |
| 706 | Wang 2014 | 10.1016/j.vaccine.2014.06.062 | English | title-abstract exclusion |
| 707 | Li 2009 | 10.1016/j.bbrc.2009.09.103 | English | title-abstract exclusion |
| 708 | Wang 2020 | 10.1080/21645515.2020.1737465 | English | title-abstract exclusion |
| 709 | Voorman 2024 | 10.1093/infdis/jiad222 | English | title-abstract exclusion |
| 710 | Sun 2016 | 10.1016/j.antiviral.2016.09.003 | English | title-abstract exclusion |
| 711 | Chen 2013 | 10.1016/j.vaccine.2012.10.032 | English | title-abstract exclusion |
| 712 | Xu 2013 | 10.1089/mab.2013.0033 | English | title-abstract exclusion |
| 713 | Yang 2014 | 10.4161/hv.28083 | English | title-abstract exclusion |
| 714 | Tan 2011 | 10.1371/journal.pone.0025662 | English | title-abstract exclusion |
| 715 | Deng 2011 | 10.1128/CVI.05132-11 | English | title-abstract exclusion |
| 716 | Tan 2016 | 10.1038/srep33663 | English | title-abstract exclusion |
| 717 | Yang 2013 | 10.3390/ijms14059618 | English | title-abstract exclusion |
| 718 | Wei 2014 | 10.3390/molecules19078949 | English | title-abstract exclusion |
| 719 | Huang 2015 | 10.1590/1414-431X20144298 | English | title-abstract exclusion |
| 720 | Huang 2014 | 10.1186/1423-0127-21-33 | English | title-abstract exclusion |
| 721 | Deng 2020 | 10.1016/j.seizure.2020.03.005 | English | title-abstract exclusion |
| 722 | Wang 2016 | 10.1186/s13104-015-1780-x | English | title-abstract exclusion |
| 723 | Pourianfar 2012 | 10.1016/j.virusres.2012.06.025 | English | title-abstract exclusion |
| 724 | Chen 2008 | 10.1016/j.vaccine.2008.03.041 | English | title-abstract exclusion |
| 725 | Du 2019 | 10.1021/acsnano.9b04071 | English | title-abstract exclusion |
| 726 | Liu 2013 | 10.1007/s00705-012-1495-4 | English | title-abstract exclusion |
| 727 | Zhang 2015 | 10.1080/21645515.2015.1008884 | English | title-abstract exclusion |
| 728 | Huang 2010 | 10.1016/j.jviromet.2009.12.015 | English | title-abstract exclusion |
| 729 | Yen 2016 | 10.1128/JVI.02057-15 | English | title-abstract exclusion |
| 730 | Chang 2010 | 10.1186/1743-422X-7-106 | English | title-abstract exclusion |
| 731 | Zhang 2014 | 10.1371/journal.pone.0083766 | English | title-abstract exclusion |
| 732 | Tan 2018 | 10.1155/2018/9254794 | English | title-abstract exclusion |
| 733 | Chen 2017 | 10.1038/s41598-017-11997-1 | English | title-abstract exclusion |
| 734 | Liu 2022 | 10.1093/femspd/ftab057 | English | title-abstract exclusion |
| 735 | Chen 2019 | 10.1017/dmp.2018.155 | English | title-abstract exclusion |
| 736 | Hu 2022 | 10.1186/s12985-022-01852-9 | English | title-abstract exclusion |
| 737 | Deng 2012 | 10.1186/1423-0127-19-73 | English | title-abstract exclusion |
| 738 | Xu 2020 | 10.1128/JVI.00204-20 | English | title-abstract exclusion |
| 739 | Wang 2015 | 10.1021/nn5063276 | English | title-abstract exclusion |
| 740 | Zhao 2013 | 10.1007/s00253-013-5257-3 | English | title-abstract exclusion |
| 741 | Duan 2022 | 10.1016/j.virs.2022.06.007 | English | title-abstract exclusion |
| 742 | Ho 2008 | 10.1128/IAI.00795-07 | English | title-abstract exclusion |
| 743 | Altamirano 2018 | 10.1093/cid/ciy650 | English | title-abstract exclusion |
| 744 | Pei 2023 | 10.3390/v15102114 | English | title-abstract exclusion |
| 745 | Yang 2012 | 10.1016/j.bmcl.2012.01.102 | English | title-abstract exclusion |
| 746 | Yang 2009 | 10.1186/1743-422X-6-141 | English | title-abstract exclusion |
| 747 | Kiener 2012 | 10.1186/1743-422X-9-55 | English | title-abstract exclusion |
| 748 | Caine 2017 | 10.1128/JVI.01759-16 | English | title-abstract exclusion |
| 749 | Kitamura 2021 | 10.3390/v13071407 | English | title-abstract exclusion |
| 750 | Pourianfar 2015 | 10.1016/j.jmii.2013.11.011 | English | title-abstract exclusion |
| 751 | Kung 2007 | 10.1111/j.1469-0691.2007.01745.x | English | title-abstract exclusion |
| 752 | Lu 2012 | 10.1128/JVI.06687-11 | English | title-abstract exclusion |
| 753 | Zhu 2023 | 10.1016/j.antiviral.2023.105553 | English | title-abstract exclusion |
| 754 | Liu 2011 | 10.1016/j.jviromet.2011.01.016 | English | title-abstract exclusion |
| 755 | BolaÃ±os-MartÃ­nez 2020 | 10.1080/14760584.2020.1791090 | English | title-abstract exclusion |
| 756 | Chan 2014 | 10.1186/1471-2458-14-11 | English | title-abstract exclusion |
| 757 | EldingLarsson 2018 | 10.1007/s00125-017-4448-3 | English | title-abstract exclusion |
| 758 | Yang 2011 | 10.1186/1743-422X-8-508 | English | title-abstract exclusion |
| 759 | Altamirano 2018 | 10.1093/cid/ciy636 | English | title-abstract exclusion |
| 760 | Huang 2018 | 10.1371/journal.pone.0191617 | English | title-abstract exclusion |
| 761 | Chen 2010 | 10.1016/j.virol.2009.12.007 | English | title-abstract exclusion |
| 762 | Liu 2011 | 10.1016/j.jviromet.2011.06.001 | English | title-abstract exclusion |
| 763 | Fisher 2017 | 10.1111/biom.12560 | English | title-abstract exclusion |
| 764 | Satoh 2019 | 10.1016/j.vaccine.2019.02.034 | English | title-abstract exclusion |
| 765 | Man-Li 2012 | 10.1002/jmv.23372 | English | title-abstract exclusion |
| 766 | Khetsuriani 2017 | 10.1016/j.vaccine.2017.04.036 | English | title-abstract exclusion |
| 767 | HuyenDTT 2019 | 10.1093/infdis/jiz124 | English | title-abstract exclusion |
| 768 | Liu 2011 | 10.1186/1743-422X-8-483 | English | title-abstract exclusion |
| 769 | Konyushko 2019 | 10.1007/s10517-019-04590-1 | English | title-abstract exclusion |
| 770 | LÃ³pez-Mena 2022 | 10.1212/WNL.0000000000200388 | English | title-abstract exclusion |
| 771 | Grifoni 2019 | 10.1016/j.humimm.2019.08.004 | English | title-abstract exclusion |
| 772 | LeTTV 2022 | 10.3389/fmicb.2022.987801 | English | title-abstract exclusion |
| 773 | Oostvogel 1994 | 10.1016/s0140-6736(94)92091-5 | English | title-abstract exclusion |
| 774 | Verbeek 2015 | 10.1212/WNL.0000000000001855 | English | title-abstract exclusion |
| 775 | Poh 2015 | 10.1007/978-1-4939-2999-3_29 | English | title-abstract exclusion |
| 776 | Lin 2018 | 10.1038/s41426-018-0165-3 | English | title-abstract exclusion |
| 777 | Kingston 2023 | 10.1099/jgv.0.001867 | English | title-abstract exclusion |
| 778 | Yoshida 2002 | 10.1099/0022-1317-83-5-1107 | English | title-abstract exclusion |
| 779 | Arita 2008 | 10.1128/JVI.01798-07 | English | title-abstract exclusion |
| 780 | Liu 2011 | 10.1016/j.vaccine.2011.04.010 | English | title-abstract exclusion |
| 781 | Wang 2013 | 10.1080/10286020.2013.770737 | English | title-abstract exclusion |
| 782 | Choudhury 1973 | <https://pmc.ncbi.nlm.nih.gov/articles/PMC2481014/> | English | title-abstract exclusion |
| 783 | Li 2022 | 10.12998/wjcc.v10.i31.11358 | English | title-abstract exclusion |
| 784 | Moss 2024 | 10.1371/journal.ppat.1012159 | English | title-abstract exclusion |
| 785 | Wang 2010 | 10.1007/s12250-010-3102-8 | English | title-abstract exclusion |
| 786 | Guo 2020 | 10.4269/ajtmh.19-0916 | English | title-abstract exclusion |
| 787 | Hamblin 1987 | 10.1017/s0950268800066590 | English | title-abstract exclusion |
| 788 | Han 2012 | 10.1007/s12250-012-3256-7 | English | title-abstract exclusion |
| 789 | Shao 2016 | 10.1371/journal.pone.0153867 | English | title-abstract exclusion |
| 790 | Huang 2024 | 10.1016/j.bj.2024.100715 | English | title-abstract exclusion |
| 791 | HossainKhan 2018 | 10.12688/f1000research.15170.3 | English | title-abstract exclusion |
| 792 | Li 2023 | 10.1371/journal.ppat.1011662 | English | title-abstract exclusion |
| 793 | Verma 2018 | 10.1073/pnas.1711923115 | English | title-abstract exclusion |
| 794 | Foo 2008 | 10.1089/vim.2007.0089 | English | title-abstract exclusion |
| 795 | Xu 2015 | 10.1371/journal.pone.0119173 | English | title-abstract exclusion |
| 796 | Hu 2023 | 10.3389/fcimb.2023.1217984 | English | title-abstract exclusion |
| 797 | LeungAKC 2022 | 10.2174/1570180820666221024095837 | English | title-abstract exclusion |
| 798 | Mugisha 2010 | 10.1111/j.1600-0684.2010.00400.x | English | title-abstract exclusion |
| 799 | Yang 2014 | 10.3390/md12074086 | English | title-abstract exclusion |
| 800 | Machado 2022 | 10.1136/annrheumdis-2021-221490 | English | title-abstract exclusion |
| 801 | Orr-Burks 2017 | 10.1038/sdata.2017.23 | English | title-abstract exclusion |
| 802 | Lin 2014 | 10.1016/j.jbiosc.2013.08.015 | English | title-abstract exclusion |
| 803 | Hu 2021 | 10.1007/s00705-021-05190-z | English | title-abstract exclusion |
| 804 | Li 2014 | 10.1371/journal.pone.0109391 | English | title-abstract exclusion |
| 805 | Liu 2007 | 10.1016/j.vaccine.2006.06.083 | English | title-abstract exclusion |
| 806 | Zhang 2014 | 10.1097/INF.0000000000000194 | English | title-abstract exclusion |
| 807 | Lin 2002 | 10.1016/s0264-410x(02)00182-2 | English | title-abstract exclusion |
| 808 | Drebot 1997 | 10.1128/aem.63.2.519-523.1997 | English | title-abstract exclusion |
| 809 | Huang 2015 | 10.1038/srep08904 | English | title-abstract exclusion |
| 810 | Hussain 2018 | 10.1016/j.vaccine.2018.02.055 | English | title-abstract exclusion |
| 811 | Orsel 2010 | 10.1016/j.tvjl.2009.06.031 | English | title-abstract exclusion |
| 812 | Gao 2018 | 10.3892/mmr.2017.7992 | English | title-abstract exclusion |
| 813 | Church 2017 | 10.1080/21645515.2017.1359454 | English | title-abstract exclusion |
| 814 | Arita 2007 | 10.1128/JVI.02856-06 | English | title-abstract exclusion |
| 815 | Jin 2018 | 10.3389/fmicb.2018.02422 | English | title-abstract exclusion |
| 816 | Xu 2014 | 10.1097/INF.0000000000000136 | English | title-abstract exclusion |
| 817 | Habib 2015 | 10.1016/j.vaccine.2014.12.001 | English | title-abstract exclusion |
| 818 | Wang 2012 | 10.1155/2012/580696 | English | title-abstract exclusion |
| 819 | Moise 2013 | 10.4161/hv.24615 | English | title-abstract exclusion |
| 820 | vanderLinden 2015 | 10.3390/v7082832 | English | title-abstract exclusion |
| 821 | Riedmann 2014 | 10.4161/hv.29344 | English | title-abstract exclusion |
| 822 | Samoilovich 2003 | 10.1002/jmv.10480 | English | title-abstract exclusion |
| 823 | Kingston 2022 | 10.1099/jgv.0.001771 | English | title-abstract exclusion |
| 824 | Tian 2019 | 10.1097/MD.0000000000017471 | English | title-abstract exclusion |
| 825 | Ochoge 2024 | 10.1016/S0140-6736(23)02844-1 | English | title-abstract exclusion |
| 826 | Wu 2004 | 10.1016/j.vaccine.2004.05.037 | English | title-abstract exclusion |
| 827 | Qi 2018 | 10.3390/ijerph15020270 | English | title-abstract exclusion |
| 828 | Yang 2012 | 10.1016/j.phymed.2012.08.012 | English | title-abstract exclusion |
| 829 | Ho 2000 | <https://www.cabidigitallibrary.org/doi/full/10.5555/20013008003> | English | title-abstract exclusion |
| 830 | Wang 2009 | 10.1586/eri.09.45 | English | title-abstract exclusion |
| 831 | Mwale 2021 | 10.3390/ijms22084146 | English | title-abstract exclusion |
| 832 | Brown 2020 | 10.3390/microorganisms8121856 | English | title-abstract exclusion |
| 833 | Han 2020 | 10.1093/ve/veaa084 | English | title-abstract exclusion |
| 834 | Bitnun 2018 | 10.1007/s11908-018-0641-x | English | title-abstract exclusion |
| 835 | VanDamme 2019 | 10.1016/S0140-6736(19)31279-6 | English | title-abstract exclusion |
| 836 | Pauly 2018 | 10.1371/journal.pone.0197370 | English | title-abstract exclusion |
| 837 | Pauksen 1994 | 10.1093/clinids/18.4.547 | English | title-abstract exclusion |
| 838 | Kovac 2015 | 10.1016/j.vaccine.2015.03.104 | English | title-abstract exclusion |
| 839 | Lee 2015 | 10.1155/2015/805039 | English | title-abstract exclusion |
| 840 | Chen 2008 | 10.1016/j.vaccine.2007.12.013 | English | title-abstract exclusion |
| 841 | Bakhache 2024 | 10.21203/rs.3.rs-3835307/v1 | English | title-abstract exclusion |
| 842 | Cowley 2019 | 10.1016/j.vaccine.2019.09.071 | English | title-abstract exclusion |
| 843 | McMinn 2002 | 10.1111/j.1574-6976.2002.tb00601.x | English | title-abstract exclusion |
| 844 | Shindarov 1979 | <https://pubmed.ncbi.nlm.nih.gov/231067/> | English | title-abstract exclusion |
| 845 | Zhang 2022 | 10.1038/s41598-022-07982-y | English | title-abstract exclusion |
| 846 | Jin 2012 | 10.1007/s12250-012-3245-x | English | title-abstract exclusion |
| 847 | Guo 2018 | 10.1097/MD.0000000000013144 | English | title-abstract exclusion |
| 848 | Lin 2003 | 10.3201/eid0903.020285 | English | title-abstract exclusion |
| 849 | Song 2016 | 10.1016/j.virusres.2016.01.002 | English | title-abstract exclusion |
| 850 | Chen 2016 | 10.1016/j.jviromet.2016.02.010 | English | title-abstract exclusion |
| 851 | Liang 2014 | 10.1038/cti.2014.24 | English | title-abstract exclusion |
| 852 | Zaman 2023 | 10.1016/S0140-6736(22)02397-2 | English | title-abstract exclusion |
| 853 | SchÃ¶nborn 2023 | 10.1016/j.jtha.2023.06.027 | English | title-abstract exclusion |
| 854 | Young 2020 | 10.3928/19382359-20200220-01 | English | title-abstract exclusion |
| 855 | POTASH 1960 | <https://www.cabidigitallibrary.org/doi/full/10.5555/19602703607> | English | title-abstract exclusion |
| 856 | Mizuta 2019 | 10.1016/j.vaccine.2018.12.065 | English | title-abstract exclusion |
| 857 | Yao 2022 | 10.15585/mmwr.mm7136a2 | English | title-abstract exclusion |
| 858 | Liu 2019 | 10.1007/s00253-018-09598-7 | English | title-abstract exclusion |
| 859 | Antanasijevic 2022 | 10.1093/pnasnexus/pgac253 | English | title-abstract exclusion |
| 860 | Ioannou 2022 | 10.7326/M22-1856 | English | title-abstract exclusion |
| 861 | Chuang 2024 | 10.1016/j.antiviral.2023.105755 | English | title-abstract exclusion |
| 862 | AdÄ±ÅŸen 2017 | 10.1016/j.clindermatol.2016.09.006 | English | title-abstract exclusion |
| 863 | Nathanson 2008 | 10.1016/S0065-3527(08)00001-8 | English | title-abstract exclusion |
| 864 | MasLago 1994 | <https://pmc.ncbi.nlm.nih.gov/articles/PMC2486525/> | English | title-abstract exclusion |
| 865 | Vancelik 2007 | <https://pubmed.ncbi.nlm.nih.gov/18219079/> | English | title-abstract exclusion |
| 866 | Pu 2015 | 10.1159/000440722 | English | title-abstract exclusion |
| 867 | Bolu 2023 | 10.11604/pamj.supp.2023.45.2.38098 | English | title-abstract exclusion |
| 868 | Basu 2004 | 10.1007/BF02724101 | English | title-abstract exclusion |
| 869 | Martin 2022 | 10.15585/mmwr.mm7124a2 | English | title-abstract exclusion |
| 870 | Song 2023 | 10.1007/s00705-023-05821-7 | English | title-abstract exclusion |
| 871 | Liang 2014 | 10.4161/hv.28456 | English | title-abstract exclusion |
| 872 | Wang 2014 | 10.1038/emi.2014.30 | English | title-abstract exclusion |
| 873 | Mbaeyi 2022 | 10.15585/mmwr.mm7142a1 | English | title-abstract exclusion |
| 874 | Pons-Salort 2015 | 10.1097/QCO.0000000000000187 | English | title-abstract exclusion |
| 875 | Liu 2011 | 10.1186/1743-422X-8-534 | English | title-abstract exclusion |
| 876 | Thoale 2013 | 10.1371/journal.pone.0069895 | English | title-abstract exclusion |
| 877 | Mir 2015 | 10.1016/S1473-3099(15)00093-6 | English | title-abstract exclusion |
| 878 | vanderSanden 2009 | 10.1128/JVI.02388-08 | English | title-abstract exclusion |
| 879 | Zhao 2009 | 10.1089/vim.2008.0073 | English | title-abstract exclusion |
| 880 | Ma 2020 | 10.1021/acsinfecdis.0c00383 | English | title-abstract exclusion |
| 881 | Tung 2007 | 10.1186/1479-0556-5-6 | English | title-abstract exclusion |
| 882 | Tang 1997 | 10.1128/JVI.71.10.7841-7850.1997 | English | title-abstract exclusion |
| 883 | Davlantes 2022 | 10.15585/mmwr.mm7123a3 | English | title-abstract exclusion |
| 884 | Shirato 2014 | 10.1016/j.vaccine.2014.07.065 | English | title-abstract exclusion |
| 885 | Wang 2024 | 10.1016/j.virusres.2023.199268 | English | title-abstract exclusion |
| 886 | Qin 2017 | 10.3892/etm.2017.4499 | English | title-abstract exclusion |
| 887 | Hu 2023 | 10.1186/s12985-023-02150-8 | English | title-abstract exclusion |
| 888 | Nagata 2002 | 10.1002/jmv.2209 | English | title-abstract exclusion |
| 889 | Arita 2022 | 10.1038/s41598-022-20544-6 | English | title-abstract exclusion |
| 890 | Wilkinson 2022 | 10.15585/mmwr.mm7115a2 | English | title-abstract exclusion |
| 891 | Blake 2018 | 10.1056/NEJMoa1716677 | English | title-abstract exclusion |
| 892 | Lin 2002 | 10.1086/343809 | English | title-abstract exclusion |
| 893 | Gantt 2013 | 10.1093/jpids/pit017 | English | title-abstract exclusion |
| 894 | Su 2008 | 10.1016/S1607-551X(09)70011-7 | English | title-abstract exclusion |
| 895 | Sadigh 2022 | 10.15585/mmwr.mm7103a3 | English | title-abstract exclusion |
| 896 | vonderHardt 2000 | 10.1016/s0264-410x(00)00092-x | English | title-abstract exclusion |
| 897 | Roberts 2015 | <https://pubmed.ncbi.nlm.nih.gov/26234257/> | English | title-abstract exclusion |
| 898 | Xu 2020 | 10.1002/smll.202002435 | English | title-abstract exclusion |
| 899 | Roberts 2020 | 10.33321/cdi.2020.44.24 | English | title-abstract exclusion |
| 900 | Michelle 2016 | <https://iris.who.int/bitstream/handle/10665/254290/WER9106_71-72.pdf> | English | title-abstract exclusion |
| 901 | Rachlin 2022 | 10.15585/mmwr.mm7119a2 | English | title-abstract exclusion |
| 902 | Roberts 2020 | 10.33321/cdi.2020.44.25 | English | title-abstract exclusion |
| 903 | Saraswathy 2004 | <https://pubmed.ncbi.nlm.nih.gov/15691149/> | English | title-abstract exclusion |
| 904 | Nair 1992 | <https://pubmed.ncbi.nlm.nih.gov/1364024/> | English | title-abstract exclusion |
| 905 | GonÃ§alves-Carneiro 2022 | 10.1038/s41564-022-01223-8 | English | title-abstract exclusion |
| 906 | Techasaensiri 2021 | 10.3390/pathogens10050625 | English | title-abstract exclusion |
| 907 | Ullmer 2018 | 10.1128/mBio.01669-18 | English | title-abstract exclusion |
| 908 | Roberts 2020 | 10.33321/cdi.2020.44.32 | English | title-abstract exclusion |
| 909 | Kaye 2022 | 10.33321/cdi.2022.46.55 | English | title-abstract exclusion |
| 910 | Georgescu 1997 | 10.1128/JVI.71.10.7758-7768.1997 | English | title-abstract exclusion |
| 911 | Hsiung 2000 | <https://pubmed.ncbi.nlm.nih.gov/10806956/> | English | title-abstract exclusion |
| 912 | Song 2014 | 10.4062/biomolther.2013.108 | English | title-abstract exclusion |
| 913 | Heath 2021 | 10.1056/NEJMoa2107659 | English | title-abstract exclusion |
| 914 | Feng 2016 | 10.12659/msm.900380 | English | title-abstract exclusion |
| 915 | Simonart 2022 | 10.2196/35034 | English | title-abstract exclusion |
| 916 | Deshpande 2014 | 10.1093/infdis/jiu204 | English | title-abstract exclusion |
| 917 | Huang 2022 | 10.3389/fphar.2022.999798 | English | title-abstract exclusion |
| 918 | Ma 2014 | 10.1007/s10616-013-9588-9 | English | title-abstract exclusion |
| 919 | Kanojia 2018 | 10.1016/j.ejpb.2018.05.021 | English | title-abstract exclusion |
| 920 | Zeng 2013 | 10.1038/emi.2013.72 | English | title-abstract exclusion |
| 921 | Burrage 2000 | 10.1016/s0264-410x(99)00542-3 | English | title-abstract exclusion |
| 922 | Ryerson 2022 | 10.15585/mmwr.mm7144e2 | English | title-abstract exclusion |
| 923 | Akhtar 2020 | 10.1093/cid/ciz1037 | English | title-abstract exclusion |
| 924 | Perez 2022 | 10.15585/mmwr.mm7140a1 | English | title-abstract exclusion |
| 925 | Li 2023 | 10.21037/tp-23-41 | English | title-abstract exclusion |
| 926 | Schoub 1986 | 10.1093/oxfordjournals.aje.a114240 | English | title-abstract exclusion |
| 927 | Link-Gelles 2022 | 10.15585/mmwr.mm7133e2 | English | title-abstract exclusion |
| 928 | Wu 2019 | 10.1007/s10616-019-00340-3 | English | title-abstract exclusion |
| 929 | Lin 2002 | 10.1086/338819 | English | title-abstract exclusion |
| 930 | Al-Qassimi 2022 | 10.1186/s12879-022-07397-0 | English | title-abstract exclusion |
| 931 | Minor 1990 | 10.1099/0022-1317-71-11-2543 | English | title-abstract exclusion |
| 932 | Rhoden 2024 | 10.1186/s12931-024-02708-2 | English | title-abstract exclusion |
| 933 | Chen 2018 | 10.1208/s12249-017-0853-7 | English | title-abstract exclusion |
| 934 | Roberts 2020 | 10.33321/cdi.2020.44.26 | English | title-abstract exclusion |
| 935 | Reichler 1997 | 10.1093/infdis/175.supplement_1.s62 | English | title-abstract exclusion |
| 936 | Zheng 2023 | 10.3390/vaccines11101606 | English | title-abstract exclusion |
| 937 | Ma 2011 | 10.1186/1743-422X-8-419 | English | title-abstract exclusion |
| 938 | Seo 2018 | 10.24171/j.phrp.2018.9.3.05 | English | title-abstract exclusion |
| 939 | Rigonan 1998 | 10.1128/JCM.36.7.1877-1881.1998 | English | title-abstract exclusion |
| 940 | Solomon 2003 | 10.1097/01.wco.0000073944.19076.56 | English | title-abstract exclusion |
| 941 | Roberts 2017 | <https://pubmed.ncbi.nlm.nih.gov/28899311/> | English | title-abstract exclusion |
| 942 | Song 2023 | 10.7774/cevr.2023.12.4.291 | English | title-abstract exclusion |
| 943 | Zhao 2017 | 10.1002/jmv.24821 | English | title-abstract exclusion |
| 944 | Snider 2023 | 10.1016/j.vaccine.2022.02.022 | English | title-abstract exclusion |
| 945 | Fiore 1997 | 10.1128/JVI.71.9.6905-6912.1997 | English | title-abstract exclusion |
| 946 | Shen 2013 | 10.1099/vir.0.046383-0 | English | title-abstract exclusion |
| 947 | Yang 2020 | 10.1128/JVI.02086-19 | English | title-abstract exclusion |
| 948 | Koike 2016 | 10.1007/978-1-4939-3292-4_7 | English | title-abstract exclusion |
| 949 | Kaye 2023 | 10.33321/cdi.2023.47.35 | English | title-abstract exclusion |
| 950 | Wang 2023 | 10.3389/fphar.2023.1164784 | English | title-abstract exclusion |
| 951 | Tait 2019 | 10.1056/NEJMoa1909953 | English | title-abstract exclusion |
| 952 | Feikin 2013 | 10.1371/journal.pmed.1001517 | English | title-abstract exclusion |
| 953 | Abzug 2014 | 10.1016/j.jinf.2013.09.020 | English | title-abstract exclusion |
| 954 | Ho 2014 | 10.1038/ncomms4344 | English | title-abstract exclusion |
| 955 | Kang 2022 | 10.1017/S0950268822000784 | English | title-abstract exclusion |
| 956 | Iliyasu 2016 | 10.1016/j.vaccine.2016.08.058 | English | title-abstract exclusion |
| 957 | O'Connor 2022 | 10.15585/mmwr.mm7109a4 | English | title-abstract exclusion |
| 958 | Wang 2014 | 10.5114/aoms.2014.43745 | English | title-abstract exclusion |
| 959 | Yusuf 2014 | 10.1093/infdis/jiu069 | English | title-abstract exclusion |
| 960 | Hegde 2016 | 10.1007/s13337-016-0340-x | English | title-abstract exclusion |
| 961 | Joseph 1987 | 10.1136/bmj.295.6591.171 | English | title-abstract exclusion |
| 962 | VosWAJW 2022 | 10.3389/fimmu.2022.982746 | English | title-abstract exclusion |
| 963 | Su 2018 | 10.1080/21645515.2018.1504540 | English | title-abstract exclusion |
| 964 | Falsey 2014 | 10.1093/infdis/jit839 | English | title-abstract exclusion |
| 965 | Kumar 2018 | 10.1007/s12098-017-2481-3 | English | title-abstract exclusion |
| 966 | Ramdass 2015 | 10.1016/j.pop.2015.08.006 | English | title-abstract exclusion |
| 967 | Papaventsis 2005 | 10.1128/AEM.71.1.72-79.2005 | English | title-abstract exclusion |
| 968 | Ahidjo 2020 | 10.1021/acsinfecdis.0c00236 | English | title-abstract exclusion |
| 969 | McCarthy 1999 | 10.1016/s0378-1135(99)00062-0 | English | title-abstract exclusion |
| 970 | Yang 2014 | 10.1038/emi.2014.35 | English | title-abstract exclusion |
| 971 | Gao 2018 | 10.1080/21645515.2017.1384107 | English | title-abstract exclusion |
| 972 | Chang 2011 | 10.1016/j.jviromet.2011.02.003 | English | title-abstract exclusion |
| 973 | Zhou 2016 | 10.4269/ajtmh.16-0062 | English | title-abstract exclusion |
| 974 | Wei 2020 | 10.1155/2020/7081219 | English | title-abstract exclusion |
| 975 | A 2021 | 10.3390/vaccines9101088 | English | title-abstract exclusion |
| 976 | Arita 2023 | 10.3390/v15040903 | English | title-abstract exclusion |
| 977 | Kumar 2015 | 10.4103/0019-557X.157536 | English | title-abstract exclusion |
| 978 | Rombaut 1990 | 10.1099/0022-1317-71-5-1081 | English | title-abstract exclusion |
| 979 | Ivanova 2022 | 10.3390/microorganisms10010112 | English | title-abstract exclusion |
| 980 | Kindberg 2009 | 10.1002/jmv.21444 | English | title-abstract exclusion |
| 981 | Kok 2015 | 10.5501/wjv.v4.i2.78 | English | title-abstract exclusion |
| 982 | Yu 2022 | 10.3389/fmicb.2022.1101850 | English | title-abstract exclusion |
| 983 | Hou 2015 | 10.1016/j.jviromet.2015.02.010 | English | title-abstract exclusion |
| 984 | Chen 2018 | 10.1017/S0950268818002509 | English | title-abstract exclusion |
| 985 | Ma 2018 | 10.7883/yoken.JJID.2017.338 | English | title-abstract exclusion |
| 986 | Sherry 2023 | 10.1128/spectrum.04300-22 | English | title-abstract exclusion |
| 987 | Semkum 2021 | 10.3390/v13061047 | English | title-abstract exclusion |
| 988 | Liu 2014 | 10.1016/j.antiviral.2014.02.015 | English | title-abstract exclusion |
| 989 | Soumajit 2019 | 10.1007/s13337-019-00559-w | English | title-abstract exclusion |
| 990 | Kohmer 2023 | 10.1016/j.jcv.2023.105471 | English | title-abstract exclusion |
| 991 | Xie 2022 | 10.3390/vaccines10111829 | English | title-abstract exclusion |
| 992 | Bakre 2017 | 10.1038/s41598-017-12860-z | English | title-abstract exclusion |
| 993 | Cao 2019 | 10.5114/ceji.2019.84009 | English | title-abstract exclusion |
| 994 | vanWijhe 2018 | 10.1097/EDE.0000000000000778 | English | title-abstract exclusion |
| 995 | Varma 2013 | 10.1155/2013/431315 | English | title-abstract exclusion |
| 996 | Song 2015 | 10.1016/j.phrp.2014.12.004 | English | title-abstract exclusion |
| 997 | Doshi 2011 | 10.1093/cid/cir332 | English | title-abstract exclusion |
| 998 | Zhou 2023 | 10.1080/07391102.2023.2263890 | English | title-abstract exclusion |
| 999 | Chen 2021 | 10.1186/s12985-021-01557-5 | English | title-abstract exclusion |
| 1000 | Anasir 2019 | 10.3389/fmicb.2019.00738 | English | title-abstract exclusion |
| 1001 | Xie 2021 | 10.3389/fmicb.2021.629533 | English | title-abstract exclusion |
| 1002 | Sui 2016 | 10.7754/clin.lab.2015.150917 | English | title-abstract exclusion |
| 1003 | Jubelt 2014 | 10.1016/B978-0-444-53488-0.00018-3 | English | title-abstract exclusion |
| 1004 | Thiele 2022 | 10.1016/j.lanepe.2021.100270 | English | title-abstract exclusion |
| 1005 | GutiÃ©rrez 1997 | 10.1128/JVI.71.5.3826-3833.1997 | English | title-abstract exclusion |
| 1006 | Rankin 2023 | 10.1001/jamanetworkopen.2022.54909 | English | title-abstract exclusion |
| 1007 | Maree 2014 | 10.2147/VMRR.S62607 | English | title-abstract exclusion |
| 1008 | He 2021 | 10.1002/prot.26157 | English | title-abstract exclusion |
| 1009 | Brindle 2023 | 10.1186/s12879-023-08300-1 | English | title-abstract exclusion |
| 1010 | Song 2014 | 10.1016/j.jgr.2014.04.003 | English | title-abstract exclusion |
| 1011 | Okemoto-Nakamura 2021 | 10.1038/s41598-021-86050-3 | English | title-abstract exclusion |
| 1012 | Rweyemamu 2008 | 10.1111/j.1865-1682.2007.01016.x | English | title-abstract exclusion |
| 1013 | Xia 2018 | 10.1002/adma.201801067 | English | title-abstract exclusion |
| 1014 | Cho 2012 | 10.1212/01.CON.0000423851.63017.2a | English | title-abstract exclusion |
| 1015 | Guo 2023 | 10.1016/j.virs.2023.05.010 | English | title-abstract exclusion |
| 1016 | Arita 2004 | 10.1099/vir.0.19768-0 | English | title-abstract exclusion |
| 1017 | Martin 2024 | 10.1016/j.jinf.2024.106145 | English | title-abstract exclusion |
| 1018 | Zhu 2018 | 10.1038/s41467-018-07531-0 | English | title-abstract exclusion |
| 1019 | Griffin 2010 | 10.1016/j.pneurobio.2009.12.003 | English | title-abstract exclusion |
| 1020 | Wang 2013 | 10.1128/CVI.00092-13 | English | title-abstract exclusion |
| 1021 | Chen 2022 | 10.3390/pathogens11111371 | English | title-abstract exclusion |
| 1022 | Chen 2019 | 10.1038/s41421-018-0073-7 | English | title-abstract exclusion |
| 1023 | Wu 2016 | 10.1371/journal.pmed.1002013 | English | title-abstract exclusion |
| 1024 | Zhang 2014 | 10.1016/j.vaccine.2014.07.071 | English | title-abstract exclusion |
| 1025 | Semmler 2023 | 10.3390/vaccines11111642 | English | title-abstract exclusion |
| 1026 | Timmermans 2016 | 10.1371/journal.pone.0152529 | English | title-abstract exclusion |
| 1027 | SaarinenNVV 2018 | 10.1038/s41598-017-18495-4 | English | title-abstract exclusion |
| 1028 | Dahri 2023 | 10.1016/j.biopha.2023.115048 | English | title-abstract exclusion |
| 1029 | Anastasina 2017 | 10.1099/jgv.0.000780 | English | title-abstract exclusion |
| 1030 | Liu 2023 | 10.1016/j.phymed.2023.155047 | English | title-abstract exclusion |
| 1031 | Mammas 2017 | 10.3892/etm.2016.3997 | English | title-abstract exclusion |
| 1032 | Blunden 2011 | 10.1136/vr.d1119 | English | title-abstract exclusion |
| 1033 | JouppilaNVV 2023 | 10.1128/spectrum.05352-22 | English | title-abstract exclusion |
| 1034 | Gundamraj 2023 | 10.1097/QCO.0000000000000922 | English | title-abstract exclusion |
| 1035 | Potshangbam 2021 | 10.2174/1389201022666210127113027 | English | title-abstract exclusion |
| 1036 | Verhey 2013 | 10.1016/B978-0-444-52910-7.00020-9 | English | title-abstract exclusion |
| 1037 | Song 2022 | 10.3390/v14112451 | English | title-abstract exclusion |
| 1038 | Cheung 2014 | 10.1016/j.antiviral.2014.09.007 | English | title-abstract exclusion |
| 1039 | Zhou 2022 | 10.1016/j.virs.2022.07.012 | English | title-abstract exclusion |
| 1040 | Wang 2013 | 10.1016/j.ab.2012.10.025 | English | title-abstract exclusion |
| 1041 | Xiao 2022 | 10.1016/j.ejmech.2022.114436 | English | title-abstract exclusion |
| 1042 | Li 2022 | 10.1038/s41467-022-35642-2 | English | title-abstract exclusion |
| 1043 | Ben-Shimol 2023 | 10.1097/INF.0000000000003865 | English | title-abstract exclusion |
| 1044 | Haldar 2022 | 10.1016/j.meegid.2022.105260 | English | title-abstract exclusion |
| 1045 | Cui 2016 | 10.1016/j.antiviral.2016.05.010 | English | title-abstract exclusion |
| 1046 | Skaletz-Rorowski 2020 | 10.1016/j.jiph.2020.04.005 | English | title-abstract exclusion |
| 1047 | Salmanton-GarcÃ­a 2024 | 10.1016/j.eclinm.2024.102553 | English | title-abstract exclusion |
| 1048 | Shah 2023 | 10.1016/j.lanepe.2023.100682 | English | title-abstract exclusion |
| 1049 | Pommier 2022 | 10.1016/S2214-109X(22)00174-7 | English | title-abstract exclusion |
| 1050 | Zhi 2020 | 10.1080/15476286.2019.1700058 | English | title-abstract exclusion |
| 1051 | Plainvert 2023 | 10.1016/j.idnow.2022.11.001 | English | title-abstract exclusion |
| 1052 | Mao 2024 | 10.1080/15548627.2024.2350270 | English | title-abstract exclusion |
| 1053 | Swain 2024 | 10.7774/cevr.2024.13.2.132 | English | title-abstract exclusion |
| 1054 | Li 2024 | 10.1038/s41467-024-48201-8 | English | title-abstract exclusion |
| 1055 | MajeBello 2024 | 10.1016/j.vaccine.2024.04.087 | English | title-abstract exclusion |
| 1056 | Huang 2024 | 10.1016/j.vaccine.2024.07.032 | English | title-abstract exclusion |
| 1057 | Kadambari 2024 | 10.1016/j.jinf.2024.106223 | English | title-abstract exclusion |
| 1058 | Yi 2023 | 10.1016/j.vaccine.2023.08.029 | English | Exclusion reason: pre-clinical study |
| 1059 | Zhou 2013 | 10.4161/hv.24395 | English | Exclusion reason: pre-clinical study |
| 1060 | Zhang 2015 | 10.1016/j.vaccine.2015.09.047 | English | Exclusion reason: pre-clinical study |
| 1061 | Cai 2014 | 10.1016/j.vaccine.2014.03.012 | English | Exclusion reason: pre-clinical study |
| 1062 | Tsou 2015 | 10.1371/journal.pntd.0003692 | English | Exclusion reason: pre-clinical study |
| 1063 | Chia 2018 | 10.1016/j.vaccine.2018.01.041 | English | Exclusion reason: pre-clinical study |
| 1064 | Yang 2020 | 10.1080/21645515.2019.1649554 | English | Exclusion reason: pre-clinical study |
| 1065 | Zheng 2023 | 10.1016/j.idm.2023.09.001 | English | Exclusion reason: modelling study |
| 1066 | Xiao 2022 | 10.1016/j.jinf.2022.06.020 | English | Exclusion reason: modelling study |
| 1067 | Du 2021 | 10.1080/21645515.2020.1763076 | English | Exclusion reason: modelling study |
| 1068 | Head 2020 | 10.1093/cid/ciz1188 | English | Exclusion reason: modelling study |
| 1069 | VanBoeckel 2016 | 10.1038/srep25248 | English | Exclusion reason: modelling study |
| 1070 | Takahashi 2016 | 10.1371/journal.pmed.1001958 | English | Exclusion reason: modelling study |
| 1071 | Wu 2022 | 10.1371/journal.pone.0274421 | English | Exclusion reason: modelling study |
| 1072 | Shi 2019 | 10.3934/mbe.2019022 | English | Exclusion reason: modelling study |
| 1073 | Shi 2018 | 10.3934/mbe.2019022 | English | Exclusion reason: modelling study |
| 1074 | Zhang 2021 | 10.1080/21645515.2020.1859900 | English | Exclusion reason: methodology |
| 1075 | Yang 2022 | 10.1038/s41467-022-34992-1 | English | Exclusion reason: immunogenicity or safety |
| 1076 | Liu 2021 | 10.3390/v13050720 | English | Exclusion reason: immunogenicity or safety |
| 1077 | Jin 2016 | 10.1080/21645515.2015.1118595 | English | Exclusion reason: immunogenicity or safety |
| 1078 | Chang 2023 | https://doi.org/10.1016/j.ijid.2023.04.162 | English | Exclusion reason: immunogenicity or safety |
| 1079 | Meng 2020 | 10.1038/s41598-020-77768-7 | English | Exclusion reason: immunogenicity or safety |
| 1080 | Guo 2022 | 10.1186/s12879-022-07661-3 | English | Exclusion reason: immunogenicity or safety |
| 1081 | Aw-Yong 2019 | 10.1002/rmv.2073 | English | Exclusion reason: immunogenicity or safety |
| 1082 | Shen 2023 | 10.1016/j.jmii.2023.10.006 | English | Exclusion reason: immunogenicity or safety |
| 1083 | Hung 2019 | 10.1016/j.vaccine.2019.07.096 | English | Exclusion reason: immunogenicity or safety |
| 1084 | Li 2021 | 10.1016/j.lanwpc.2021.100284 | English | Exclusion reason: immunogenicity or safety |
| 1085 | Tong 2022 | 10.1016/j.eclinm.2022.101596 | English | Exclusion reason: immunogenicity or safety |
| 1086 | Liu 2015 | 10.1186/s12916-015-0448-7 | English | Exclusion reason: immunogenicity or safety |
| 1087 | Gu 2018 | 10.1080/14760584.2018.1430572 | English | Exclusion reason: immunogenicity or safety |
| 1088 | Hu 2018 | 10.1080/21645515.2018.1442997 | English | Exclusion reason: immunogenicity or safety |
| 1089 | Liu 2022 | 10.3390/vaccines10060895 | English | Exclusion reason: immunogenicity or safety |
| 1090 | Zhu 2017 | 10.1080/14760584.2017.1335603 | English | Exclusion reason: immunogenicity or safety |
| 1091 | Zhang 2021 | 10.1093/jpids/piaa129 | English | Exclusion reason: immunogenicity or safety |
| 1092 | Chen 2022 | 10.3389/fimmu.2022.1080408 | English | Exclusion reason: immunogenicity or safety |
| 1093 | Gao 2021 | 10.1016/j.vaccine.2021.01.048 | English | Exclusion reason: immunogenicity or safety |
| 1094 | Tambyah 2019 | 10.1016/j.vaccine.2019.06.023 | English | Exclusion reason: immunogenicity or safety |
| 1095 | Chen 2014 | 10.4161/hv.28397 | English | Exclusion reason: immunogenicity or safety |
| 1096 | Zhu 2012 | 10.1097/INF.0b013e31826eba74 | English | Exclusion reason: immunogenicity or safety |
| 1097 | Wang 2014 | 10.1093/infdis/jiu113 | English | Exclusion reason: immunogenicity or safety |
| 1098 | Xu 2020 | 10.1016/j.vaccine.2020.02.013 | English | Exclusion reason: immunogenicity or safety |
| 1099 | Chen 2022 | 10.1080/21645515.2022.2063630 | English | Exclusion reason: immunogenicity or safety |
| 1100 | Liu 2021 | 10.1080/21645515.2021.2010428 | English | Exclusion reason: immunogenicity or safety |
| 1101 | Zhao 2022 | 10.1016/j.vaccine.2022.06.027 | English | Exclusion reason: immunogenicity or safety |
| 1102 | Mao 2013 | 10.1371/journal.pone.0079599 | English | Exclusion reason: immunogenicity or safety |
| 1103 | Zhang 2019 | 10.1093/infdis/jiz129 | English | Exclusion reason: immunogenicity or safety |
| 1104 | Zhu 2013 | 10.1016/S0140-6736(12)61764-4 | English | Exclusion reason: immunogenicity or safety |
| 1105 | Hu 2013 | 10.1128/CVI.00491-13 | English | Exclusion reason: immunogenicity or safety |
| 1106 | Liu 2013 | 10.1371/journal.pone.0054451 | English | Exclusion reason: immunogenicity or safety |
| 1107 | Li 2014 | 10.1093/infdis/jit429 | English | Exclusion reason: immunogenicity or safety |
| 1108 | Xiao 2023 | 10.3390/vaccines11040862 | English | Exclusion reason: immunogenicity or safety |
| 1109 | He 2022 | 10.3389/fmicb.2021.811553 | English | Exclusion reason: descriptive study |
| 1110 | Wang 2021 | 10.1016/j.vaccine.2021.05.009 | English | Exclusion reason: descriptive study |
| 1111 | Duan 2022 | 10.1080/22221751.2022.2125346 | English | Exclusion reason: descriptive study |
| 1112 | Jiang 2021 | 10.1080/22221751.2021.1899772 | English | Exclusion reason: descriptive study |
| 1113 | Sun 2022 | 10.1097/MD.0000000000030356 | English | Exclusion reason: descriptive study |
| 1114 | Yang 2023 | 10.1186/s12985-023-02169-x | English | Exclusion reason: descriptive study |
| 1115 | Jiang 2021 | 10.1186/s12879-021-06462-4 | English | Exclusion reason: descriptive study |
| 1116 | Han 2020 | 10.1097/INF.0000000000002745 | English | Exclusion reason: descriptive study |
| 1117 | Li 2021 | 10.1080/21645515.2020.1851129 | English | Exclusion reason: descriptive study |
| 1118 | Li 2016 | 10.3389/fmicb.2016.00391 | English | Exclusion reason: descriptive study |
| 1119 | Kang 2022 | 10.1017/S0950268822000784 | English | Exclusion reason: descriptive study |
| 1120 | Li 2023 | 10.3390/vaccines11050944 | English | Exclusion reason: descriptive study |
| 1121 | Li 2023 | 10.1016/j.vaccine.2023.09.006 | English | Exclusion reason: descriptive study |
| 1122 | Chen 2022 | 10.1155/2022/9156186 | English | Exclusion reason: descriptive study |
| 1123 | Hong 2022 | 10.1016/j.lanwpc.2021.100370 | English | Exclusion reason: descriptive study |
| 1124 | Wang 2022 | 10.1038/s41598-022-21312-2 | English | Exclusion reason: descriptive study |
| 1125 | Huang 2022 | 10.1186/s12889-022-13860-z | English | Exclusion reason: descriptive study |
| 1126 | Peng 2020 | 10.1038/s41598-020-63274-3 | English | Exclusion reason: descriptive study |
| 1127 | He 2021 | 10.3389/fmicb.2021.811553 | English | Exclusion reason: descriptive study |
| 1128 | Li 2019 | 10.1371/journal.pone.0217474 | English | Exclusion reason: descriptive study |
| 1129 | Huang 2022 | 10.1186/s12889-022-13941-z | English | Exclusion reason: descriptive study |
| 1130 | Mao 2016 | 10.1038/emi.2016.73 | English | Exclusion reason: progress and review |
| 1131 | Chang 2018 | 10.1590/S1678-9946201860070 | English | Exclusion reason: progress and review |
| 1132 | Klein 2015 | 10.1080/21645515.2015.1049780 | English | Exclusion reason: progress and review |
| 1133 | Yee 2016 | 10.3390/v8010001 | English | Exclusion reason: progress and review |
| 1134 | Bello 2022 | 10.1016/j.biologicals.2022.08.007 | English | Exclusion reason: progress and review |
| 1135 | Yee 2016 | 10.2174/1381612822666160720165613 | English | Exclusion reason: progress and review |
| 1136 | Zhou 2016 | 10.1080/14760584.2016.1191357 | English | Exclusion reason: progress and review |
| 1137 | He 2021 | 10.1007/s12250-020-00294-3 | English | Exclusion reason: progress and review |
| 1138 | Mao 2016 | 10.1586/14760584.2016.1138862 | English | Exclusion reason: progress and review |
| 1139 | Fang 2018 | 10.1080/14760584.2018.1510326 | English | Exclusion reason: progress and review |
| 1140 | Zhu 2023 | 10.1186/s12929-023-00908-4 | English | Exclusion reason: progress and review |
| 1141 | Klein 2015 | 10.1586/14760584.2015.993385 | English | Exclusion reason: progress and review |
| 1142 | Zhang 2010 | 10.1016/j.ijid.2009.12.002 | English | Exclusion reason: progress and review |
| 1143 | Chong 2015 | 10.1093/cid/ciu852 | English | Exclusion reason: progress and review |
| 1144 | Reed 2016 | 10.1016/j.vaccine.2016.02.077 | English | Exclusion reason: progress and review |
| 1145 | Liu 2015 | 10.1016/j.micinf.2014.11.004 | English | Exclusion reason: progress and review |
| 1146 | Lei 2020 | 10.1016/j.vaccine.2020.05.001 | English | Exclusion reason: progress and review |
| 1147 | Li 2014 | 10.4161/hv.27253 | English | Exclusion reason: progress and review |
| 1148 | Li 2014 | 10.1586/14760584.2014.897617 | English | Exclusion reason: progress and review |
| 1149 | Chang 2016 | 10.3390/ijerph13090890 | English | Exclusion reason: progress and review |
| 1150 | Lu 2014 | 10.1038/emi.2014.29 | English | Exclusion reason: progress and review |
| 1151 | Nguyen-Tran 2022 | 10.1016/S0140-6736(22)00380-4 | English | Exclusion reason: progress and review |
| 1152 | Mao 2014 | 10.1016/S1473-3099(14)70972-7 | English | Exclusion reason: progress and review |
| 1153 | Crawford 2013 | 10.1016/S0140-6736(13)61124-1 | English | Exclusion reason: progress and review |
| 1154 | Riedmann 2013 | https://www.webofscience.com/wos/woscc/full-record/WOS:000328572700005 | English | Exclusion reason: progress and review |
| 1155 | Zhang 2022 | 10.3390/ijms24010169 | English | Exclusion reason: progress and review |
| 1156 | Aswathyraj 2016 | 10.1007/s00430-016-0465-y | English | Exclusion reason: progress and review |
| 1157 | Lin 2019 | 10.1186/s12929-019-0560-7 | English | Exclusion reason: progress and review |
| 1158 | Kinobe 2022 | 10.1002/rmv.2361 | English | Exclusion reason: progress and review |
| 1159 | Yee 2015 | 10.3390/v8010001 | English | Exclusion reason: progress and review |
| 1160 | Ng 2015 | 10.3390/v7122949 | English | Exclusion reason: progress and review |
| 1161 | Lee 2010 | 10.1586/erv.09.152 | English | Exclusion reason: progress and review |
| 1162 | Tan 2021 | 10.1016/S1473-3099(20)30452-7 | English | Exclusion reason: progress and review |
| 1163 | Li 2021 | 10.3390/vaccines9030199 | English | Exclusion reason: progress and review |
| 1164 | Yi 2017 | 10.7774/cevr.2017.6.1.4 | English | Exclusion reason: progress and review |
| 1165 | Zhang 2024 | 10.1080/21645515.2024.2330163 | English | Inclusion |
| 1166 | Jiang 2020 | 10.1016/j.vaccine.2019.12.025 | English | Inclusion |
| 1167 | Li 2019 | 10.1016/S2352-4642(19)30185-3 | English | Inclusion |
| 1168 | Wang 2019 | 10.1080/21645515.2019.1581539 | English | Inclusion |
| 1169 | Wang 2023 | 10.3389/fpubh.2023.1162209 | English | Inclusion |
| 1170 | Li 2016 | 10.1586/14760584.2016.1096782 | English | Inclusion |
| 1171 | Guan 2020 | 10.1093/cid/ciz1114 | English | Inclusion |
| 1172 | Wei 2017 | 10.1093/infdis/jiw502 | English | Inclusion |
| 1173 | Zhu 2014 | 10.1056/NEJMoa1304923 | English | Inclusion |
| 1174 | Li 2014 | 10.1056/NEJMoa1303224 | English | Inclusion |
| 1175 | Zhu 2013 | 10.1016/S0140-6736(13)61049-1 | English | Inclusion |
| 1176 | Nguyen 2022 | 10.1016/S0140-6736(22)00313-0 | English | Inclusion |
| 1177 | Duan 2024 | https://doi.org/10.1016/j.vaccine.2024.02.026 | English | Inclusion |
| 1178 | Wan Li 2023 | https://doi.org/10.20043/j.cnki.MPM.202307514 | Chinese | title-abstract exclusion |
| 1179 | Breast Cancer Group, Oncology Physicians Branch, Chinese Medical Doctor Association 2020 | 10.3760/cma.j.cn112152-20200410-00330 | Chinese | title-abstract exclusion |
| 1180 | Chinese Center for Disease Control and Prevention 2016 | https://d.wanfangdata.com.cn/periodical/ChlQZXJpb2RpY2FsQ0hJTmV3UzIwMjMxMjI2Eg96Z2pobXkyMDE2MDQwMjAaCGN5anZzNHpv | Chinese | title-abstract exclusion |
| 1181 | Qiao Rui 2020 | https://doi.org/10.19829/j.zgfybj.issn.1001-4411.2020.05.003 | Chinese | title-abstract exclusion |
| 1182 | Yu Yong 2015 | https://doi.org/10.13213/j.cnki.jeom.2015.15382 | Chinese | title-abstract exclusion |
| 1183 | Tong Kai 2019 | https://doi.org/10.13431/j.cnki.immunol.j.20190073 | Chinese | title-abstract exclusion |
| 1184 | Wu Hansheng 2023 | https://doi.org/10.3760/cma.j.cn441206-20230203-00013 | Chinese | title-abstract exclusion |
| 1185 | He Fan 2010 | https://doi.org/10.3760/cma.j.issn.0254-6450.2010.05.020 | Chinese | title-abstract exclusion |
| 1186 | Yu Wenmin 2019 | https://doi.org/10.3969/j.issn.1001-6325.2019.03.010 | Chinese | title-abstract exclusion |
| 1187 | Yu Qiufan 2019 | https://doi.org/10.3760/cma.j.issn.1003-9279.2019.06.017 | Chinese | title-abstract exclusion |
| 1188 | Hou Yunde 2018 | https://doi.org/10.3760/cma.j.issn.2095-428X.2018.18.001 | Chinese | title-abstract exclusion |
| 1189 | Guan Qihua 2011 | https://d.wanfangdata.com.cn/periodical/ChlQZXJpb2RpY2FsQ0hJTmV3UzIwMjMxMjI2Eg96Z3h4d3MyMDExMDQwNTgaCGN5anZzNHpv | Chinese | title-abstract exclusion |
| 1190 | Feng Yiping 2022 | https://doi.org/10.6040/j.issn.1671-7554.0.2021.0707 | Chinese | title-abstract exclusion |
| 1191 | Feng Jing 2018 | https://doi.org/10.16168/j.cnki.issn.1002-9982.2018.04.015 | Chinese | title-abstract exclusion |
| 1192 | Liu Shike 2021 | https://d.wanfangdata.com.cn/periodical/ChlQZXJpb2RpY2FsQ0hJTmV3UzIwMjMxMjI2Eg94ZHlmeXgyMDIxMDEwMjQaCGN5anZzNHpv | Chinese | title-abstract exclusion |
| 1193 | Liu Jingtao 2010 | https://doi.org/10.3760/cma.j.issn.0578-1310.2010.01.006 | Chinese | title-abstract exclusion |
| 1194 | Liu Fengqin 2010 | https://doi.org/10.3870/yydb.2010.05.023 | Chinese | title-abstract exclusion |
| 1195 | Liu Tian 2017 | https://d.wanfangdata.com.cn/periodical/ChlQZXJpb2RpY2FsQ0hJTmV3UzIwMjMxMjI2Eg94ZHlmeXgyMDE3MjQwMDEaCGN5anZzNHpv | Chinese | title-abstract exclusion |
| 1196 | Liu Xinfeng 2017 | https://doi.org/10.11847/zgggws2017-33-04-30 | Chinese | title-abstract exclusion |
| 1197 | Liu Haixia 2021 | https://doi.org/10.3969/j.issn.1002-3674.2021.06.020 | Chinese | title-abstract exclusion |
| 1198 | Liu Runyou 2014 | https://d.wanfangdata.com.cn/periodical/ChlQZXJpb2RpY2FsQ0hJTmV3UzIwMjMxMjI2Eg94ZHlmeXgyMDE0MjEwMjUaCGN5anZzNHpv | Chinese | title-abstract exclusion |
| 1199 | Liu Xiangwen 2017 | https://doi.org/10.16155/j.0254-1793.2017.12.12 | Chinese | title-abstract exclusion |
| 1200 | Liu Li 2019 | https://doi.org/10.12114/j.issn.1007-9572.2018.00.088 | Chinese | title-abstract exclusion |
| 1201 | Liu Chao 2022 | https://doi.org/10.11847/zgggws1133517 | Chinese | title-abstract exclusion |
| 1202 | Liu Gequn 2020 | https://doi.org/10.11847/zgggws1127276 | Chinese | title-abstract exclusion |
| 1203 | Shan Zhuzhou 2017 | https://doi.org/10.3969/j.issn.1002-1949.2017.z1.031 | Chinese | title-abstract exclusion |
| 1204 | Lu Yan 2016 | https://doi.org/10.3969/j.issn.1671-8348.2016.29.015 | Chinese | title-abstract exclusion |
| 1205 | Xiang Yunfeng 2017 | https://doi.org/10.3969/j.issn.1673-5501.2017.05.013 | Chinese | title-abstract exclusion |
| 1206 | Xiang Lunhui 2015 | https://doi.org/10.16462/j.cnki.zhjbkz.2015.02.009 | Chinese | title-abstract exclusion |
| 1207 | Lü Ziping 2012 | https://d.wanfangdata.com.cn/periodical/ChlQZXJpb2RpY2FsQ0hJTmV3UzIwMjMxMjI2Eg56eXl4azIwMTIwMzAyMBoIY3lqdnM0em8%3D | Chinese | title-abstract exclusion |
| 1208 | Lü Likun 2017 | https://doi.org/10.13350/j.cjpb.170104 | Chinese | title-abstract exclusion |
| 1209 | Wu Sihan 2017 | https://doi.org/10.16462/j.cnki.zhjbkz.2017.11.009 | Chinese | title-abstract exclusion |
| 1210 | Wu Xinwei 2009 | https://doi.org/10.3969/j.issn.1001-9448.2009.05.050 | Chinese | title-abstract exclusion |
| 1211 | Wu Xiaona 2012 | https://d.wanfangdata.com.cn/periodical/ChlQZXJpb2RpY2FsQ0hJTmV3UzIwMjMxMjI2Eg9qYmt6enoyMDEyMDMwMTYaCGN5anZzNHpv | Chinese | title-abstract exclusion |
| 1212 | Wu Yanjia 2021 | https://d.wanfangdata.com.cn/periodical/ChlQZXJpb2RpY2FsQ0hJTmV3UzIwMjMxMjI2Eg94ZHlmeXgyMDIxMDYwMTgaCGN5anZzNHpv | Chinese | title-abstract exclusion |
| 1213 | Zhou Min 2015 | https://doi.org/10.3969/j.issn.1002-266X.2015.28.007 | Chinese | title-abstract exclusion |
| 1214 | Zhou Hao 2023 | https://doi.org/10.16506/j.1009-6639.2023.02.005 | Chinese | title-abstract exclusion |
| 1215 | Yao Huilin 2016 | https://d.wanfangdata.com.cn/periodical/ChlQZXJpb2RpY2FsQ0hJTmV3UzIwMjMxMjI2Eg94ZHlmeXgyMDE2MDIwMjMaCGN5anZzNHpv | Chinese | title-abstract exclusion |
| 1216 | Yao Rongmei 2019 | https://doi.org/10.13242/j.cnki.bingduxuebao.003559 | Chinese | title-abstract exclusion |
| 1217 | Jiang Wenguo 2011 | https://d.wanfangdata.com.cn/periodical/ChlQZXJpb2RpY2FsQ0hJTmV3UzIwMjMxMjI2EhN6Z2pzY2Jmenp6MjAxMTExMDE5GghjeWp2czR6bw%3D%3D | Chinese | title-abstract exclusion |
| 1218 | Sun Jianwei 2010 | https://d.wanfangdata.com.cn/periodical/ChlQZXJpb2RpY2FsQ0hJTmV3UzIwMjMxMjI2Eg94ZHlmeXgyMDEwMDIwMDUaCGN5anZzNHpv | Chinese | title-abstract exclusion |
| 1219 | Sun Jingyi 2015 | https://d.wanfangdata.com.cn/periodical/ChlQZXJpb2RpY2FsQ0hJTmV3UzIwMjMxMjI2Eg96Z3h4d3MyMDE1MDYwNDYaCGN5anZzNHpv | Chinese | title-abstract exclusion |
| 1220 | Sun Qiuyun 2017 | https://d.wanfangdata.com.cn/periodical/ChlQZXJpb2RpY2FsQ0hJTmV3UzIwMjMxMjI2Eg96Z3dzdGoyMDE3MDMwMjcaCGN5anZzNHpv | Chinese | title-abstract exclusion |
| 1221 | Sun Ping 2022 | https://doi.org/10.6039/j.issn.1001-0408.2022.04.21 | Chinese | title-abstract exclusion |
| 1222 | Sun Xiaxia 2018 | https://d.wanfangdata.com.cn/periodical/ChlQZXJpb2RpY2FsQ0hJTmV3UzIwMjMxMjI2Eg94ZHlmeXgyMDE4MDQwMDIaCGN5anZzNHpv | Chinese | title-abstract exclusion |
| 1223 | Meng Haorong 2021 | https://doi.org/10.3760/cma.j.cn112338-20200423-00634 | Chinese | title-abstract exclusion |
| 1224 | An Qingyu 2011 | https://doi.org/10.3969/j.issn.1002-3674.2011.03.034 | Chinese | title-abstract exclusion |
| 1225 | Song Juan 2019 | https://doi.org/10.13242/j.cnki.bingduxuebao.003537 | Chinese | title-abstract exclusion |
| 1226 | Song Xiaomei 2015 | https://doi.org/10.16016/j.1000-5404.201408005 | Chinese | title-abstract exclusion |
| 1227 | Song Aili 2010 | https://d.wanfangdata.com.cn/periodical/ChlQZXJpb2RpY2FsQ0hJTmV3UzIwMjMxMjI2Eg94ZHlmeXgyMDEwMDcwMjcaCGN5anZzNHpv | Chinese | title-abstract exclusion |
| 1228 | Song Yang 2022 | https://doi.org/10.11821/dlxb202203006 | Chinese | title-abstract exclusion |
| 1229 | Xuan Zhaopeng 2007 | https://doi.org/10.3760/cma.j.issn.1001-2036.2007.04.017 | Chinese | title-abstract exclusion |
| 1230 | Yin Hanlin 2021 | https://doi.org/10.13193/j.issn.1673-7717.2021.10.026 | Chinese | title-abstract exclusion |
| 1231 | Cui Yajie 2019 | https://doi.org/10.3760/cma.j.issn.1000-6680.2019.06.003 | Chinese | title-abstract exclusion |
| 1232 | Cui Yajie 2017 | https://doi.org/10.7499/j.issn.1008-8830.2017.08.012 | Chinese | title-abstract exclusion |
| 1233 | Tie Jinfeng 2011 | https://d.wanfangdata.com.cn/periodical/ChlQZXJpb2RpY2FsQ0hJTmV3UzIwMjMxMjI2EhB6Z3hkeHp6MjAxMTA2MDAxGghjeWp2czR6bw%3D%3D | Chinese | title-abstract exclusion |
| 1234 | Chang Wenling 2022 | https://doi.org/10.20043/j.cnki.MPM.202204650 | Chinese | title-abstract exclusion |
| 1235 | Zhang Deyong 2015 | https://d.wanfangdata.com.cn/periodical/ChlQZXJpb2RpY2FsQ0hJTmV3UzIwMjMxMjI2Eg94ZHlmeXgyMDE1MDEwMDYaCGN5anZzNHpv | Chinese | title-abstract exclusion |
| 1236 | Zhang Huijuan 2010 | https://doi.org/10.3760/cma.j.issn.0254-5101.2010.05.002 | Chinese | title-abstract exclusion |
| 1237 | Zhang Xin 2009 | https://doi.org/10.3760/cma.j.issn.0254-5101.2009.04.007 | Chinese | title-abstract exclusion |
| 1238 | Zhang Xiangxue 2019 | https://d.wanfangdata.com.cn/periodical/ChlQZXJpb2RpY2FsQ0hJTmV3UzIwMjMxMjI2Eg9kcXh4a3gyMDE5MDMwMDkaCGN5anZzNHpv | Chinese | title-abstract exclusion |
| 1239 | Zhang Qi 2009 | https://d.wanfangdata.com.cn/periodical/ChlQZXJpb2RpY2FsQ0hJTmV3UzIwMjMxMjI2Eg96Z3h4d3MyMDA5MDcwNDIaCGN5anZzNHpv | Chinese | title-abstract exclusion |
| 1240 | Zhang Shuo 2014 | https://d.wanfangdata.com.cn/periodical/ChlQZXJpb2RpY2FsQ0hJTmV3UzIwMjMxMjI2Eg1iZHhiMjAxNDAzMDAxGghjeWp2czR6bw%3D%3D | Chinese | title-abstract exclusion |
| 1241 | Zhang Yan 2020 | https://doi.org/10.19829/j.zgfybj.issn.1001-4411.2020.15.029 | Chinese | title-abstract exclusion |
| 1242 | Zhang Daha 2023 | https://doi.org/10.12114/j.issn.1007-9572.2022.0771 | Chinese | title-abstract exclusion |
| 1243 | Zhang Tiehui 2018 | https://doi.org/10.3969/j.issn.2095-4344.0062 | Chinese | title-abstract exclusion |
| 1244 | Peng Li 2018 | https://d.wanfangdata.com.cn/periodical/ChlQZXJpb2RpY2FsQ0hJTmV3UzIwMjMxMjI2Eg1sZHl4MjAxODA4MDAyGghjeWp2czR6bw%3D%3D | Chinese | title-abstract exclusion |
| 1245 | Xu Yingli 2023 | https://d.wanfangdata.com.cn/periodical/ChlQZXJpb2RpY2FsQ0hJTmV3UzIwMjMxMjI2EhB6eXlseWxjMjAyMzAyMDA2GghjeWp2czR6bw%3D%3D | Chinese | title-abstract exclusion |
| 1246 | Xu Hongxu 2010 | https://doi.org/10.3760/cma.j.issn.1000-6680.2010.07.007 | Chinese | title-abstract exclusion |
| 1247 | Qi Li 2010 | https://doi.org/10.3969/j.issn.1002-266X.2010.23.030 | Chinese | title-abstract exclusion |
| 1248 | Wen Han 2016 | https://d.wanfangdata.com.cn/periodical/ChlQZXJpb2RpY2FsQ0hJTmV3UzIwMjMxMjI2Eg94ZHlmeXgyMDE2MTYwMDMaCGN5anZzNHpv | Chinese | title-abstract exclusion |
| 1249 | Fang Xuehui 2010 | https://doi.org/10.3781/j.issn.1000-7431.2010.12.018 | Chinese | title-abstract exclusion |
| 1250 | Fang Jie 2024 | https://doi.org/10.3760/cma.j.cn114453-20230505-00096 | Chinese | title-abstract exclusion |
| 1251 | Fang Jie 2023 | https://doi.org/10.3760/cma.j.cn114453-20230217-00035 | Chinese | title-abstract exclusion |
| 1252 | Zhu Qiang 2009 | https://doi.org/10.3760/cma.j.issn.1672-7088.2009.09.120 | Chinese | title-abstract exclusion |
| 1253 | Zhu Zhihong 2015 | https://doi.org/10.16168/j.cnki.issn.1002-9982.2015.01.22 | Chinese | title-abstract exclusion |
| 1254 | Zhu Huijuan 2020 | https://doi.org/10.13457/j.cnki.jncm.2020.08.014 | Chinese | title-abstract exclusion |
| 1255 | Zhu Wenbing 2020 | https://doi.org/10.13376/j.cbls/20120125 | Chinese | title-abstract exclusion |
| 1256 | Zhu Lei 2019 | https://doi.org/10.3760/cma.j.issn.1000-6680.2019.02.009 | Chinese | title-abstract exclusion |
| 1257 | Li Li 2021 | https://doi.org/10.19829/j.zgfybj.issn.1001-4411.2021.24.072 | Chinese | title-abstract exclusion |
| 1258 | Li Yanan 2016 | https://doi.org/10.13220/j.cnki.jipr.2016.05.005 | Chinese | title-abstract exclusion |
| 1259 | Li Jianqing 2009 | https://doi.org/10.3969/j.issn.1006-5725.2009.18.045 | Chinese | title-abstract exclusion |
| 1260 | Li Dongmei 2018 | https://doi.org/10.15938/j.jhust.2018.01.027 | Chinese | title-abstract exclusion |
| 1261 | Li Xuejun 2017 | https://doi.org/10.16241/j.cnki.1001-5914.2017.06.008 | Chinese | title-abstract exclusion |
| 1262 | Li Xuechuan 2023 | https://doi.org/10.7507/1002-1892.202310038 | Chinese | title-abstract exclusion |
| 1263 | Li Xiaozhou 2014 | https://d.wanfangdata.com.cn/periodical/ChlQZXJpb2RpY2FsQ0hJTmV3UzIwMjMxMjI2Eg96Z3dzdGoyMDE0MDIwMTQaCGN5anZzNHpv | Chinese | title-abstract exclusion |
| 1264 | Li Wenqing 2016 | https://doi.org/10.3760/cma.j.issn.1001-2036.2016.06.028 | Chinese | title-abstract exclusion |
| 1265 | Li Wenbin 2009 | https://d.wanfangdata.com.cn/periodical/ChlQZXJpb2RpY2FsQ0hJTmV3UzIwMjMxMjI2EhB6Z3hkeHp6MjAwOTA0MDY2GghjeWp2czR6bw%3D%3D | Chinese | title-abstract exclusion |
| 1266 | Li Youbin 2023 | https://doi.org/10.3760/cma.j.cn441206-20230419-00069 | Chinese | title-abstract exclusion |
| 1267 | Li Jie 2012 | https://doi.org/10.3760/cma.j.issn.0254-6450.2012.09.011 | Chinese | title-abstract exclusion |
| 1268 | Li Yang 2016 | https://d.wanfangdata.com.cn/periodical/ChlQZXJpb2RpY2FsQ0hJTmV3UzIwMjMxMjI2Eg94ZHlmeXgyMDE2MTMwMzAaCGN5anZzNHpv | Chinese | title-abstract exclusion |
| 1269 | Li Hongjiao 2021 | https://doi.org/10.13288/j.11-2166/r.2021.11.018 | Chinese | title-abstract exclusion |
| 1270 | Li Qinli 2014 | https://d.wanfangdata.com.cn/periodical/ChlQZXJpb2RpY2FsQ0hJTmV3UzIwMjMxMjI2Eg94ZHlmeXgyMDE0MTMwMzIaCGN5anZzNHpv | Chinese | title-abstract exclusion |
| 1271 | Li Hongmei 2016 | 10.3969/j.issn.1671-8348.2016.13.011 | Chinese | title-abstract exclusion |
| 1272 | Li Yixuan 2020 | https://doi.org/10.3969/j.issn.1672-8467.2020.04.008 | Chinese | title-abstract exclusion |
| 1273 | Li Yuan 2019 | https://doi.org/10.16168/j.cnki.issn.1002-9982.2019.12.013 | Chinese | title-abstract exclusion |
| 1274 | Li Rong 2011 | https://d.wanfangdata.com.cn/periodical/ChlQZXJpb2RpY2FsQ0hJTmV3UzIwMjMxMjI2Eg94ZHlmeXgyMDExMjEwMTgaCGN5anZzNHpv | Chinese | title-abstract exclusion |
| 1275 | Li Weilin 2001 | https://d.wanfangdata.com.cn/periodical/ChlQZXJpb2RpY2FsQ0hJTmV3UzIwMjMxMjI2Eg96aGhsenoyMDAxMDIwMTYaCGN5anZzNHpv | Chinese | title-abstract exclusion |
| 1276 | Li Lianchu 2012 | https://doi.org/10.3760/cma.j.issn.1009-4598.2012.02.022 | Chinese | title-abstract exclusion |
| 1277 | Du Ruixiao 2020 | <https://d.wanfangdata.com.cn/periodical/zgswzpxzz202001015> | Chinese | title-abstract exclusion |
| 1278 | Yang Rendong 2016 | https://doi.org/10.11847/zgggws2016-32-01-15 | Chinese | title-abstract exclusion |
| 1279 | Yang Zhuo 2014 | <https://qikan.cqvip.com/Qikan/Article/Detail?id=49238167> | Chinese | title-abstract exclusion |
| 1280 | Yang Guojing 2021 | https://d.wanfangdata.com.cn/periodical/ChlQZXJpb2RpY2FsQ0hJTmV3UzIwMjMxMjI2Eg94ZHlmeXgyMDIxMTAwMDMaCGN5anZzNHpv | Chinese | title-abstract exclusion |
| 1281 | Yang Yuejie 2014 | https://doi.org/10.3760/cma.j.issn.2095-4352.2014.12.018 | Chinese | title-abstract exclusion |
| 1282 | Yang Xu 2018 | https://doi.org/10.16781/j.0258-879x.2018.02.0226 | Chinese | title-abstract exclusion |
| 1283 | Yang Bo 2017 | https://doi.org/10.11847/zgggws2017-33-06-01 | Chinese | title-abstract exclusion |
| 1284 | Yang Bo 2015 | https://d.wanfangdata.com.cn/periodical/ChlQZXJpb2RpY2FsQ0hJTmV3UzIwMjMxMjI2Eg96Z3h4d3MyMDE1MDIwMjgaCGN5anZzNHpv | Chinese | title-abstract exclusion |
| 1285 | Yang Xiang 2021 | <https://d.wanfangdata.com.cn/periodical/zgswzpxzz202107015> | Chinese | title-abstract exclusion |
| 1286 | Yang Surong 2012 | https://d.wanfangdata.com.cn/periodical/ChlQZXJpb2RpY2FsQ0hJTmV3UzIwMjMxMjI2Eg96Z2Z5YmoyMDEyMjEwMDUaCGN5anZzNHpv | Chinese | title-abstract exclusion |
| 1287 | Yang Liangjun 2020 | https://doi.org/10.3760/cma.j.cn441206-20200523-00240 | Chinese | title-abstract exclusion |
| 1288 | Yang Jin 2014 | https://doi.org/10.3760/cma.j.issn.1001-8050.2014.06.029 | Chinese | title-abstract exclusion |
| 1289 | Yang Peng 2018 | https://doi.org/10.16835/j.cnki.1000-9817.2018.03.008 | Chinese | title-abstract exclusion |
| 1290 | Lin Ling 2020 | https://doi.org/10.3760/cma.j.cn441206-20200223-00084 | Chinese | title-abstract exclusion |
| 1291 | Cha Jie 2015 | https://d.wanfangdata.com.cn/periodical/ChlQZXJpb2RpY2FsQ0hJTmV3UzIwMjMxMjI2EhF6Z3dzanl6ejIwMTUwMjAwMxoIY3lqdnM0em8%3D | Chinese | title-abstract exclusion |
| 1292 | Liu Nan 2013 | https://doi.org/10.7644/j.issn.1674-9960.2013.12.019 | Chinese | title-abstract exclusion |
| 1293 | Liang Pu 2018 | https://doi.org/10.3760/cma.j.issn.1003-9279.2018.02.020 | Chinese | title-abstract exclusion |
| 1294 | Fan Huan 2016 | https://doi.org/10.3760/cma.j.issn.0254-6450.2016.12.011 | Chinese | title-abstract exclusion |
| 1295 | Duan Lina 2016 | https://doi.org/10.16835/j.cnki.1000-9817.2016.08.026 | Chinese | title-abstract exclusion |
| 1296 | Hong Zhimin 2018 | https://doi.org/10.13860/j.cnki.sltj.20171013-002 | Chinese | title-abstract exclusion |
| 1297 | Hong Jie 2022 | https://doi.org/10.3760/cma.j.cn112338-20211221-01006 | Chinese | title-abstract exclusion |
| 1298 | Pan Weiyi 2020 | 10.13200/j.cnki.cjb.003009 | Chinese | title-abstract exclusion |
| 1299 | Pan Menghua 2020 | https://doi.org/10.16462/j.cnki.zhjbkz.2020.11.006 | Chinese | title-abstract exclusion |
| 1300 | Pan Jie 2016 | https://doi.org/10.16241/j.cnki.1001-5914.2016.05.001 | Chinese | title-abstract exclusion |
| 1301 | Pan Hao 2011 | <https://d.wanfangdata.com.cn/periodical/ChlQZXJpb2RpY2FsQ0hJTmV3UzIwMjMxMjI2Eg9qYmt6enoyMDExMDUwMjQaCGN5anZzNHpv> | Chinese | title-abstract exclusion |
| 1302 | Pan Hongbo 2014 | https://doi.org/10.3969/j.issn.1006-5725.2014.01.012 | Chinese | title-abstract exclusion |
| 1303 | Pan Qingsong 2012 | https://d.wanfangdata.com.cn/periodical/ChlQZXJpb2RpY2FsQ0hJTmV3UzIwMjMxMjI2EhBzaG15eHp6MjAxMjAzMDAzGghjeWp2czR6bw%3D%3D | Chinese | title-abstract exclusion |
| 1304 | Niu Dong 2021 | https://doi.org/10.3969/j.issn.1005-4847.2021.02.011 | Chinese | title-abstract exclusion |
| 1305 | Wang Ling 2016 | https://d.wanfangdata.com.cn/periodical/ChlQZXJpb2RpY2FsQ0hJTmV3UzIwMjMxMjI2Eg96Z3dzdGoyMDE2MDUwMzQaCGN5anZzNHpv | Chinese | title-abstract exclusion |
| 1306 | Wang Jia 2021 | https://doi.org/10.11816/cn.ni.2021-203382 | Chinese | title-abstract exclusion |
| 1307 | Wang Peiji 2009 | https://doi.org/10.3760/cma.j.issn.1005-054X.2009.06.016 | Chinese | title-abstract exclusion |
| 1308 | Wang Shu 2020 | https://doi.org/10.11726/j.issn.1001-7658.2020.08.025 | Chinese | title-abstract exclusion |
| 1309 | Wang Yuqian 2020 | https://doi.org/10.13242/j.cnki.bingduxuebao.003827 | Chinese | title-abstract exclusion |
| 1310 | Wang Yue 2023 | https://doi.org/10.16506/j.1009-6639.2023.03.015 | Chinese | title-abstract exclusion |
| 1311 | Wang Chunyang 2019 | https://doi.org/10.3969/j.issn.1000-484X.2019.16.007 | Chinese | title-abstract exclusion |
| 1312 | Wang Xiaorui 2023 | https://doi.org/10.3784/jbjc.202304170169 | Chinese | title-abstract exclusion |
| 1313 | Wang Meng 2023 | https://doi.org/10.20043/j.cnki.MPM.202301339 | Chinese | title-abstract exclusion |
| 1314 | Wang Bo 2010 | https://d.wanfangdata.com.cn/periodical/ChlQZXJpb2RpY2FsQ0hJTmV3UzIwMjMxMjI2EhZ6Z3N3aHh5Znpzd3hiMjAxMDA2MDA4GghjeWp2czR6bw%3D%3D | Chinese | title-abstract exclusion |
| 1315 | Wang Ruiping 2019 | https://d.wanfangdata.com.cn/periodical/ChlQZXJpb2RpY2FsQ0hJTmV3UzIwMjMxMjI2Eg94ZHlmeXgyMDE5MTEwMDEaCGN5anZzNHpv | Chinese | title-abstract exclusion |
| 1316 | Wang Lei 2017 | https://doi.org/10.11816/cn.ni.2017-170709 | Chinese | title-abstract exclusion |
| 1317 | Wang Xiuping 2016 | https://doi.org/10.3760/cma.j.issn.1672-7088.2016.z1.054 | Chinese | title-abstract exclusion |
| 1318 | Wang Wei 2019 | https://d.wanfangdata.com.cn/periodical/ChlQZXJpb2RpY2FsQ0hJTmV3UzIwMjMxMjI2Eg94ZHlmeXgyMDE5MDUwMDEaCGN5anZzNHpv | Chinese | title-abstract exclusion |
| 1319 | Wang Meinü 2020 | https://doi.org/10.3760/cma.j.cn311653-20190927-00272 | Chinese | title-abstract exclusion |
| 1320 | Wang Zifang 2021 | https://doi.org/10.3760/cma.j.cn441206-20190420-00161 | Chinese | title-abstract exclusion |
| 1321 | Wang Ying 2011 | https://d.wanfangdata.com.cn/periodical/ChlQZXJpb2RpY2FsQ0hJTmV3UzIwMjMxMjI2EhF6Z2V0Ymp6ejIwMTEwNTAzMRoIY3lqdnM0em8%3D | Chinese | title-abstract exclusion |
| 1322 | Wang Rui 2023 | https://doi.org/10.13242/j.cnki.bingduxuebao.004282 | Chinese | title-abstract exclusion |
| 1323 | Wang Chao 2015 | https://doi.org/10.3760/cma.j.issn.0254-6450.2015.05.014 | Chinese | title-abstract exclusion |
| 1324 | Wang Jing 2015 | https://doi.org/10.3969/j.issn.1006-5725.2015.13.055 | Chinese | title-abstract exclusion |
| 1325 | Wang Xianping 2012 | https://d.wanfangdata.com.cn/periodical/ChlQZXJpb2RpY2FsQ0hJTmV3UzIwMjMxMjI2EhJ6aHl5Z3J4enoyMDEyMTQwMjIaCGN5anZzNHpv | Chinese | title-abstract exclusion |
| 1326 | Wang Dingsheng 2020 | https://d.wanfangdata.com.cn/periodical/ChlQZXJpb2RpY2FsQ0hJTmV3UzIwMjMxMjI2EhJ6Z2RmYmZ6enoyMDIwMDUwMzUaCGN5anZzNHpv | Chinese | title-abstract exclusion |
| 1327 | Bai Lina 2016 | https://d.wanfangdata.com.cn/periodical/ChlQZXJpb2RpY2FsQ0hJTmV3UzIwMjMxMjI2EhF6Z3dzanl6ejIwMTYyMDAzMxoIY3lqdnM0em8%3D | Chinese | title-abstract exclusion |
| 1328 | Zhu Miao 2017 | https://doi.org/10.11844/cjcb.2017.09.0009 | Chinese | title-abstract exclusion |
| 1329 | Cheng Liyang 2006 | https://doi.org/10.3969/j.issn.1004-5872.2006.05.016 | Chinese | title-abstract exclusion |
| 1330 | Fu Xia 2016 | https://doi.org/10.16462/j.cnki.zhjbkz.2016.05.022 | Chinese | title-abstract exclusion |
| 1331 | Lian Wei 2021 | https://doi.org/10.7655/nydxbns20210111 | Chinese | title-abstract exclusion |
| 1332 | Luo Tongyong 2012 | https://d.wanfangdata.com.cn/periodical/ChlQZXJpb2RpY2FsQ0hJTmV3UzIwMjMxMjI2Eg96Z3h4d3MyMDEyMDQwMzcaCGN5anZzNHpv | Chinese | title-abstract exclusion |
| 1333 | Luo Ming 2011 | https://doi.org/10.3760/cma.j.issn.1009-9158.2011.08.018 | Chinese | title-abstract exclusion |
| 1334 | Xiao Xinglong 2009 | https://doi.org/10.3321/j.issn:0001-6209.2009.01.017 | Chinese | title-abstract exclusion |
| 1335 | Xiao Xincai 2012 | https://d.wanfangdata.com.cn/periodical/ChlQZXJpb2RpY2FsQ0hJTmV3UzIwMjMxMjI2Eg96Z3h4d3MyMDEyMDUwMDcaCGN5anZzNHpv | Chinese | title-abstract exclusion |
| 1336 | Hu Shouyong 2021 | https://doi.org/10.3760/cma.j.cn311653-20200820-00314 | Chinese | title-abstract exclusion |
| 1337 | Lu Jing 2011 | https://d.wanfangdata.com.cn/periodical/ChlQZXJpb2RpY2FsQ0hJTmV3UzIwMjMxMjI2EhF6Z3dzanl6ejIwMTEwOTA5MBoIY3lqdnM0em8%3D | Chinese | title-abstract exclusion |
| 1338 | Miao Ping 2023 | https://doi.org/10.3760/cma.j.cn311653-20230726-00010 | Chinese | title-abstract exclusion |
| 1339 | Fan Rongjun 2017 | https://d.wanfangdata.com.cn/periodical/ChlQZXJpb2RpY2FsQ0hJTmV3UzIwMjMxMjI2EhF6Z3dzanl6ejIwMTcxNDAzOBoIY3lqdnM0em8%3D | Chinese | title-abstract exclusion |
| 1340 | Chi Jing 2016 | https://doi.org/10.3760/cma.j.issn.0253-9624.2016.03.015 | Chinese | title-abstract exclusion |
| 1341 | Dong Xiaochun 2018 | https://d.wanfangdata.com.cn/periodical/ChlQZXJpb2RpY2FsQ0hJTmV3UzIwMjMxMjI2Eg94ZHlmeXgyMDE4MTQwMDEaCGN5anZzNHpv | Chinese | title-abstract exclusion |
| 1342 | Jiang Lina 2017 | https://doi.org/10.16462/j.cnki.zhjbkz.2017.04.005 | Chinese | title-abstract exclusion |
| 1343 | Jiang Xiaojuan 2015 | https://doi.org/10.16462/j.cnki.zhjbkz.2015.11.028 | Chinese | title-abstract exclusion |
| 1344 | Cai Shaojian 2014 | https://doi.org/10.11847/zgggws2014-30-11-33 | Chinese | title-abstract exclusion |
| 1345 | Xue Lin 2015 | https://doi.org/10.16462/j.cnki.zhjbkz.2015.07.003 | Chinese | title-abstract exclusion |
| 1346 | Yuan Guoping 2012 | https://d.wanfangdata.com.cn/periodical/ChlQZXJpb2RpY2FsQ0hJTmV3UzIwMjMxMjI2Eg1sZHl4MjAxMjA2MDAxGghjeWp2czR6bw%3D%3D | Chinese | title-abstract exclusion |
| 1347 | Yuan Yuerong 2021 | https://doi.org/10.16168/j.cnki.issn.1002-9982.2021.09.010 | Chinese | title-abstract exclusion |
| 1348 | Yuan Qi 2017 | https://doi.org/10.13193/j.issn.1673-7717.2017.09.039 | Chinese | title-abstract exclusion |
| 1349 | Chu Wei 2022 | https://doi.org/10.3760/cma.j.cn112866-20220624-00143 | Chinese | title-abstract exclusion |
| 1350 | Qin Jiangchun 2021 | https://d.wanfangdata.com.cn/periodical/ChlQZXJpb2RpY2FsQ0hJTmV3UzIwMjMxMjI2Eg94ZHlmeXgyMDIxMTgwMzQaCGN5anZzNHpv | Chinese | title-abstract exclusion |
| 1351 | Xu Yanqing 2009 | https://doi.org/10.3321/j.issn:1005-4529.2009.13.036 | Chinese | title-abstract exclusion |
| 1352 | Tan Ting 2014 | https://doi.org/10.11817/j.issn.1672-7347.2014.11.011 | Chinese | title-abstract exclusion |
| 1353 | Xie Jing 2015 | https://d.wanfangdata.com.cn/periodical/ChlQZXJpb2RpY2FsQ0hJTmV3UzIwMjMxMjI2Eg1iZHhiMjAxNTA1MDA0GghjeWp2czR6bw%3D%3D | Chinese | title-abstract exclusion |
| 1354 | Xie Guangcheng 2019 | https://doi.org/10.13242/j.cnki.bingduxuebao.003484 | Chinese | title-abstract exclusion |
| 1355 | He Xiaonan 2019 | https://d.wanfangdata.com.cn/periodical/ChlQZXJpb2RpY2FsQ0hJTmV3UzIwMjMxMjI2Eg94ZHlmeXgyMDE5MDMwMDUaCGN5anZzNHpv | Chinese | title-abstract exclusion |
| 1356 | Jia Shuangshuang 2016 | https://doi.org/10.16155/j.0254-1793.2016.06.07 | Chinese | title-abstract exclusion |
| 1357 | Jia Siyue 2019 | https://doi.org/10.16462/j.cnki.zhjbkz.2019.03.002 | Chinese | title-abstract exclusion |
| 1358 | Jia Jing 2019 | https://d.wanfangdata.com.cn/periodical/ChlQZXJpb2RpY2FsQ0hJTmV3UzIwMjMxMjI2Eg94ZHlmeXgyMDE5MTgwMDIaCGN5anZzNHpv | Chinese | title-abstract exclusion |
| 1359 | Lai Fawei 2017 | https://doi.org/10.11726/j.issn.1001-7658.2017.12.006 | Chinese | title-abstract exclusion |
| 1360 | Lai Shengjie 2011 | https://doi.org/10.3760/cma.j.issn.0254-6450.2011.06.011 | Chinese | title-abstract exclusion |
| 1361 | Lai Huibing 2021 | https://d.wanfangdata.com.cn/periodical/ChlQZXJpb2RpY2FsQ0hJTmV3UzIwMjMxMjI2Eg94ZHlmeXgyMDIxMTIwMzMaCGN5anZzNHpv | Chinese | title-abstract exclusion |
| 1362 | Zhao Jianxiong 2013 | https://doi.org/10.11653/syfj2013170316 | Chinese | title-abstract exclusion |
| 1363 | Zhao Guohong 2013 | https://doi.org/10.3760/cma.j.issn.1001-2036.2013.06.018 | Chinese | title-abstract exclusion |
| 1364 | Zhao Bin 2019 | https://d.wanfangdata.com.cn/periodical/ChlQZXJpb2RpY2FsQ0hJTmV3UzIwMjMxMjI2EhF6Z3lrZHh4YjIwMTkwMzAyMBoIY3lqdnM0em8%3D | Chinese | title-abstract exclusion |
| 1365 | Zhao Xinhua 2010 | https://d.wanfangdata.com.cn/periodical/ChlQZXJpb2RpY2FsQ0hJTmV3UzIwMjMxMjI2EhB6Z3hkeHp6MjAxMDAxMDY2GghjeWp2czR6bw%3D%3D | Chinese | title-abstract exclusion |
| 1366 | Zhao Yuna 2022 | https://doi.org/10.13286/j.1001-5213.2022.22.21 | Chinese | title-abstract exclusion |
| 1367 | Zhao Yanting 2014 | https://d.wanfangdata.com.cn/periodical/ChlQZXJpb2RpY2FsQ0hJTmV3UzIwMjMxMjI2Eg96Z2prankyMDE0MDkwMDkaCGN5anZzNHpv | Chinese | title-abstract exclusion |
| 1368 | Zhao Yingjie 2009 | https://d.wanfangdata.com.cn/periodical/ChlQZXJpb2RpY2FsQ0hJTmV3UzIwMjMxMjI2EhF6Z3dzanl6ejIwMDkwNTA4ORoIY3lqdnM0em8%3D | Chinese | title-abstract exclusion |
| 1369 | Zhao Jing 2010 | https://d.wanfangdata.com.cn/periodical/ChlQZXJpb2RpY2FsQ0hJTmV3UzIwMjMxMjI2Eg96Z2prankyMDEwMDIwMjUaCGN5anZzNHpv | Chinese | title-abstract exclusion |
| 1370 | Lu Laijin 2008 | https://d.wanfangdata.com.cn/periodical/ChlQZXJpb2RpY2FsQ0hJTmV3UzIwMjMxMjI2EhN6Z3hmY2p3a3p6MjAwODEyMDE1GghjeWp2czR6bw%3D%3D | Chinese | title-abstract exclusion |
| 1371 | Deng Te 2013 | https://d.wanfangdata.com.cn/periodical/ChlQZXJpb2RpY2FsQ0hJTmV3UzIwMjMxMjI2Eg96Z3dzdGoyMDEzMDMwMDgaCGN5anZzNHpv | Chinese | title-abstract exclusion |
| 1372 | Zheng Guanzeng 2011 | https://d.wanfangdata.com.cn/periodical/ChlQZXJpb2RpY2FsQ0hJTmV3UzIwMjMxMjI2EhF6Z3dzanl6ejIwMTExMTAwNhoIY3lqdnM0em8%3D | Chinese | title-abstract exclusion |
| 1373 | Guo Shuying 2013 | https://doi.org/10.3760/cma.j.issn.0254-5101.2013.05.015 | Chinese | title-abstract exclusion |
| 1374 | Guo Shanshan 2012 | <https://doi.org/10.3969/j.issn.1005-9903.2012.02.060> | Chinese | title-abstract exclusion |
| 1375 | Guo Nana 2013 | https://d.wanfangdata.com.cn/periodical/ChlQZXJpb2RpY2FsQ0hJTmV3UzIwMjMxMjI2Eg96Z2prankyMDEzMDkwMTQaCGN5anZzNHpv | Chinese | title-abstract exclusion |
| 1376 | Guo Yue 2017 | https://d.wanfangdata.com.cn/periodical/ChlQZXJpb2RpY2FsQ0hJTmV3UzIwMjMxMjI2Eg1iZHhiMjAxNzA2MDEwGghjeWp2czR6bw%3D%3D | Chinese | title-abstract exclusion |
| 1377 | Chen Bing 2009 | https://d.wanfangdata.com.cn/periodical/ChlQZXJpb2RpY2FsQ0hJTmV3UzIwMjMxMjI2Eg94ZHlmeXgyMDA5MjEwNjgaCGN5anZzNHpv | Chinese | title-abstract exclusion |
| 1378 | Chen Jiawen 2014 | https://d.wanfangdata.com.cn/periodical/ChlQZXJpb2RpY2FsQ0hJTmV3UzIwMjMxMjI2Eg96Z3h5enoyMDE0MTYwMjQaCGN5anZzNHpv | Chinese | title-abstract exclusion |
| 1379 | Chen Guoping 2018 | https://doi.org/10.16462/j.cnki.zhjbkz.2018.02.013 | Chinese | title-abstract exclusion |
| 1380 | Chen Hongbiao 2015 | https://d.wanfangdata.com.cn/periodical/ChlQZXJpb2RpY2FsQ0hJTmV3UzIwMjMxMjI2Eg94ZHlmeXgyMDE1MTMwMDYaCGN5anZzNHpv | Chinese | title-abstract exclusion |
| 1381 | Chen Mimi 2017 | https://doi.org/10.11816/cn.ni.2017-172019 | Chinese | title-abstract exclusion |
| 1382 | Chen Heng 2022 | https://doi.org/10.12182/20220560104 | Chinese | title-abstract exclusion |
| 1383 | Chen Min 2014 | https://doi.org/10.11816/cn.ni.2014-133842 | Chinese | title-abstract exclusion |
| 1384 | Chen Wenjuan 2010 | https://d.wanfangdata.com.cn/periodical/ChlQZXJpb2RpY2FsQ0hJTmV3UzIwMjMxMjI2Eg96Z2Z5YmoyMDEwMjEwMDYaCGN5anZzNHpv | Chinese | title-abstract exclusion |
| 1385 | Chen Jie 2015 | https://doi.org/10.11816/cn.ni.2015-141299 | Chinese | title-abstract exclusion |
| 1386 | Chen Yuwen 2011 | https://doi.org/10.3969/j.issn.1006-5725.2011.18.060 | Chinese | title-abstract exclusion |
| 1387 | Chen Fang 2019 | https://doi.org/10.3760/cma.j.issn.2095-428X.2019.10.008 | Chinese | title-abstract exclusion |
| 1388 | Tao Junwen 2020 | https://d.wanfangdata.com.cn/periodical/ChlQZXJpb2RpY2FsQ0hJTmV3UzIwMjMxMjI2Eg94ZHlmeXgyMDIwMjEwMDQaCGN5anZzNHpv | Chinese | title-abstract exclusion |
| 1389 | Tao Zhongsheng 2010 | https://d.wanfangdata.com.cn/periodical/ChlQZXJpb2RpY2FsQ0hJTmV3UzIwMjMxMjI2EhF6Z2p4d2t6ejIwMTAxMjAyNRoIY3lqdnM0em8%3D | Chinese | title-abstract exclusion |
| 1390 | Sui Meili 2015 | https://doi.org/10.13705/j.issn.1671-6825.2015.01.006 | Chinese | title-abstract exclusion |
| 1391 | Ya Xuerong 2011 | https://d.wanfangdata.com.cn/periodical/ChlQZXJpb2RpY2FsQ0hJTmV3UzIwMjMxMjI2EhF6Z3dzanl6ejIwMTEwOTA3MRoIY3lqdnM0em8%3D | Chinese | title-abstract exclusion |
| 1392 | Wei Yiyun 2013 | https://d.wanfangdata.com.cn/periodical/ChlQZXJpb2RpY2FsQ0hJTmV3UzIwMjMxMjI2Eg96Z3dzdGoyMDEzMDYwMDQaCGN5anZzNHpv | Chinese | title-abstract exclusion |
| 1393 | Wei Yiyun 2014 | https://d.wanfangdata.com.cn/periodical/ChlQZXJpb2RpY2FsQ0hJTmV3UzIwMjMxMjI2Eg94ZHlmeXgyMDE0MTMwMDEaCGN5anZzNHpv | Chinese | title-abstract exclusion |
| 1394 | Gu Songye 2009 | https://d.wanfangdata.com.cn/periodical/ChlQZXJpb2RpY2FsQ0hJTmV3UzIwMjMxMjI2EhF6Z3dzanl6ejIwMDkwNzEwNxoIY3lqdnM0em8%3D | Chinese | title-abstract exclusion |
| 1395 | Yan Bingxin 2016 | https://d.wanfangdata.com.cn/periodical/ChlQZXJpb2RpY2FsQ0hJTmV3UzIwMjMxMjI2Eg94ZHlmeXgyMDE2MDUwNDEaCGN5anZzNHpv | Chinese | title-abstract exclusion |
| 1396 | Yan Yubing 2017 | https://doi.org/10.16462/j.cnki.zhjbkz.2017.08.015 | Chinese | title-abstract exclusion |
| 1397 | Yan Yubing 2016 | https://doi.org/10.16168/j.cnki.issn.1002-9982.2016.04.017 | Chinese | title-abstract exclusion |
| 1398 | Ma Tao 2016 | https://d.wanfangdata.com.cn/periodical/ChlQZXJpb2RpY2FsQ0hJTmV3UzIwMjMxMjI2Eg94ZHlmeXgyMDE2MjMwMjEaCGN5anZzNHpv | Chinese | title-abstract exclusion |
| 1399 | Ma Te 2009 | https://d.wanfangdata.com.cn/periodical/ChlQZXJpb2RpY2FsQ0hJTmV3UzIwMjMxMjI2EhBqZ2xseXNqMjAwOTEyMDA5GghjeWp2czR6bw%3D%3D | Chinese | title-abstract exclusion |
| 1400 | Ma Fuyuan 2018 | https://doi.org/10.3760/cma.j.issn.1001-2036.2018.06.032 | Chinese | title-abstract exclusion |
| 1401 | Ma Hongxia 2017 | https://d.wanfangdata.com.cn/periodical/ChlQZXJpb2RpY2FsQ0hJTmV3UzIwMjMxMjI2EhF6Z3dzanl6ejIwMTcwNzAxNBoIY3lqdnM0em8%3D | Chinese | title-abstract exclusion |
| 1402 | Gao Yunyun 2018 | https://doi.org/10.16462/j.cnki.zhjbkz.2018.10.021 | Chinese | title-abstract exclusion |
| 1403 | Gao Junqing 2009 | https://d.wanfangdata.com.cn/periodical/ChlQZXJpb2RpY2FsQ0hJTmV3UzIwMjMxMjI2EhN6Z3hmY2p3a3p6MjAwOTAxMDE1GghjeWp2czR6bw%3D%3D | Chinese | title-abstract exclusion |
| 1404 | Gao Jianqing 2015 | https://d.wanfangdata.com.cn/periodical/ChlQZXJpb2RpY2FsQ0hJTmV3UzIwMjMxMjI2Eg96Z25qenoyMDE1MTIwMDgaCGN5anZzNHpv | Chinese | title-abstract exclusion |
| 1405 | Gao Yi 2019 | https://doi.org/10.12114/j.issn.1007-9572.2018.00.102 | Chinese | title-abstract exclusion |
| 1406 | Gao Qiuj 2020 | https://doi.org/10.16462/j.cnki.zhjbkz.2020.01.015 | Chinese | title-abstract exclusion |
| 1407 | Gao Ya 2017 | https://doi.org/10.11847/zgggws2017-33-10-16 | Chinese | title-abstract exclusion |
| 1408 | Wei Lingyun 2010 | https://doi.org/10.3969/j.issn.1671-9638.2010.03.007 | Chinese | title-abstract exclusion |
| 1409 | Wei Jing 2012 | https://d.wanfangdata.com.cn/periodical/ChlQZXJpb2RpY2FsQ0hJTmV3UzIwMjMxMjI2Eg96Z3h4d3MyMDEyMDkwMzMaCGN5anZzNHpv | Chinese | title-abstract exclusion |
| 1410 | Huang Guo 2019 | https://d.wanfangdata.com.cn/periodical/ChlQZXJpb2RpY2FsQ0hJTmV3UzIwMjMxMjI2Eg96Z3dzdGoyMDE5MDEwMTUaCGN5anZzNHpv | Chinese | title-abstract exclusion |
| 1411 | Huang Jian 2012 | <https://d.wanfangdata.com.cn/periodical/zgswzpxzz201203033> | Chinese | title-abstract exclusion |
| 1412 | Huang Xueyong 2010 | <https://d.wanfangdata.com.cn/periodical/zgjscbfzzz201008018> | Chinese | title-abstract exclusion |
| 1413 | Huang Wenxian 2014 | https://doi.org/10.3969/j.issn.1001-1528.2014.07.056 | Chinese | title-abstract exclusion |
| 1414 | Huang Hao 2021 | https://doi.org/10.13242/j.cnki.bingduxuebao.003996 | Chinese | title-abstract exclusion |
| 1415 | Huang Lei 2011 | https://doi.org/10.3760/cma.j.issn.1007-631X.2011.11.016 | Chinese | title-abstract exclusion |
| 1416 | Huang Lei 2012 | https://doi.org/10.3760/cma.j.issn.1007-8118.2012.05.010 | Chinese | title-abstract exclusion |
| 1417 | Huang Yanzhi 2016 | https://d.wanfangdata.com.cn/periodical/ChlQZXJpb2RpY2FsQ0hJTmV3UzIwMjMxMjI2EhB6Z3N5emR4MjAxNjAzMDI3GghjeWp2czR6bw%3D%3D | Chinese | title-abstract exclusion |
| 1418 | Li Qian 2023 | https://doi.org/10.20043/j.cnki.MPM.202207398 | Chinese | title-abstract exclusion |
| 1419 | Qi Xiaoqi 2023 | https://doi.org/10.16506/j.1009-6639.2023.09.014 | Chinese | title-abstract exclusion |
| 1420 | Mao Zhenxing 2022 | 10. 19914 /j. CJVI. 2022043 | Chinese | Exclusion reason: clinical-confirmed outcomes |
| 1421 | Zhao Zheng 2023 | https://doi.org/10.3760/cma.j.cn112338-20220417-00310 | Chinese | Exclusion reason: progress and review |
| 1422 | Xu Wenjie 2020 | https://doi.org/10.3760/cma.j.cn112150-20200515-00735 | Chinese | Exclusion reason: progress and review |
| 1423 | Bi Fangchuan 2021 | 10.3969/j.issn.1000-3606.2021.05.016 | Chinese | Exclusion reason: progress and review |
| 1424 | Tang Xueqin 2022 | https://doi.org/10.12182/20220860103 | Chinese | Exclusion reason: descriptive study |
| 1425 | Liu Gequn 2020 | 10.11847/zgggws1127276 | Chinese | Exclusion reason: descriptive study |
| 1426 | Lai Huibing 2021 | http://xdyfyxzz.paperopen.com/#/digest?ArticleID=13612 | Chinese | Exclusion reason: descriptive study |
| 1427 | Yuan Yuan 2014 | https://doi.org/10.13431/j.cnki.immunol.j.20140102 | Chinese | Exclusion reason: pre-clinical study |
| 1428 | Zhang Lin 2022 | https://doi.org/10.19914/j.CJVI.2022009 | Chinese | Exclusion reason: methodology |
| 1429 | Zhang Li 2020 | https://doi.org/10.3760/cma.j.issn.0254-6450.2020.02.024 | Chinese | Exclusion reason: methodology |
| 1430 | Hua Ruijue 2020 | https://doi.org/10.3760/cma.j.cn112338-20191006-00717 | Chinese | Inclusion |
