# Supplementary material for: The efficacy and effectiveness of enterovirus A71 vaccines against hand, foot, and mouth disease: A systematic review and meta-analysis
Source: PLoS One. 2025 May 22;20(5):e0323782. doi: 10.1371/journal.pone.0323782 (PMC12097632; doi:10.1371/journal.pone.0323782)
Supplement: S4 Table — (DOCX) [file pone.0323782.s004.docx]

**Supporting information**

**The efficacy and effectiveness of enterovirus A71 vaccines against hand, foot, and mouth disease: a systematic review and meta-analysis**

# **S4 Table. All data extracted from the primary research sources for the systematic review and meta-analysis.**

| No. | study | Site | Period | Design | Case confirmation | Control | Sample size | Age | gender | Vaccine | Reference group | Dose | Vaccination duration | Outcome | VE (95%CI) |
| --- | --- | --- | --- | --- | --- | --- | --- | --- | --- | --- | --- | --- | --- | --- | --- |
| 1 | Li Y (2019) | Zhengzhou | 2017-2018 | TND | PCR | tested negative for EV-A71 | 1803 | 6-71 months | all | inactivated EV-A71 vaccines | unvaccinated | partial |  | EV-A71 HFMD hospitalisation | 63.1 (13.1, 84.3) |
| 1 | Li Y (2019) | Zhengzhou | 2017-2018 | TND | PCR | tested negative for EV-A71 | 1803 | 6-71 months | all | inactivated EV-A71 vaccines | unvaccinated | full |  | EV-A71 HFMD hospitalisation | 85.4 (53.2, 95.4) |
| 1 | Li Y (2019) | Zhengzhou | 2017-2018 | TND | PCR | tested negative for EV-A71 | 1803 | 6-71 months | all | inactivated EV-A71 vaccines | unvaccinated | full |  | CVA-16 infection | –10.1 (–72.6, 29.7) |
| 1 | Li Y (2019) | Zhengzhou | 2017-2018 | TND | PCR | tested negative for EV-A71 | 1803 | 6-71 months | all | inactivated EV-A71 vaccines | unvaccinated | full |  | CVA-6 infection | –2.5 (–51.4, 30.7) |
| 1 | Li Y (2019) | Zhengzhou | 2017-2018 | TND | PCR | tested negative for EV-A71 | 1803 | 6-23 months | all | inactivated EV-A71 vaccines | unvaccinated | full |  | EV-A71 HFMD hospitalisation | 78 (7.2, 94.8) |
| 1 | Li Y (2019) | Zhengzhou | 2017-2018 | TND | PCR | tested negative for EV-A71 | 1803 | 24-71 months | all | inactivated EV-A71 vaccines | unvaccinated | full |  | EV-A71 HFMD hospitalisation | 91.1 (33.6, 98.8) |
| 1 | Li Y (2019) | Zhengzhou | 2017-2018 | TND | PCR | tested negative for EV-A71 | 1803 | 6-71 months | all | inactivated EV-A71 vaccines | unvaccinated | full |  | mild EV-A71 HFMD hospitalisation | 91.1 (35.1, 98.8) |
| 1 | Li Y (2019) | Zhengzhou | 2017-2018 | TND | PCR | tested negative for EV-A71 | 1803 | 6-71 months | all | inactivated EV-A71 vaccines | unvaccinated | full |  | severe EV-A71 HFMD hospitalisation | 73.3 (–32.6, 94.6) |
| 1 | Li Y (2019) | Zhengzhou | 2017-2018 | TND | PCR | tested negative for EV-A71 | 1803 | 6-23 months | all | inactivated EV-A71 vaccines | unvaccinated | partial |  | EV-A71 HFMD hospitalisation | 40.8 (–71.1, 79.5) |
| 1 | Li Y (2019) | Zhengzhou | 2017-2018 | TND | PCR | tested negative for EV-A71 | 1803 | 24-71 months | all | inactivated EV-A71 vaccines | unvaccinated | partial |  | EV-A71 HFMD hospitalisation | 77.9 (4.3, 94.9) |
| 1 | Li Y (2019) | Zhengzhou | 2017-2018 | TND | PCR | tested negative for EV-A71 | 1803 | 6-71 months | all | inactivated EV-A71 vaccines | unvaccinated | partial |  | mild EV-A71 HFMD hospitalisation | 50.5 (–27.4, 80.8) |
| 1 | Li Y (2019) | Zhengzhou | 2017-2018 | TND | PCR | tested negative for EV-A71 | 1803 | 6-71 months | all | inactivated EV-A71 vaccines | unvaccinated | partial |  | severe EV-A71 HFMD hospitalisation | 47 (–512.1, 95.4) |
| 2 | Guan XH (2019) | Hubei | 2016-2017 | cohort | q-RT-PCR |  | 155995 | 6-71 months | all | CAMS | unvaccinated and partially vaccinated | full |  | EV-A71 HFMD | 94.5 (94.2, 94.8) |
| 2 | Guan XH (2019) | Hubei | 2016-2017 | cohort | q-RT-PCR |  | 155995 | 6-35 months | all | CAMS | unvaccinated and partially vaccinated | full |  | EV-A71 HFMD | 90.8 (31.5, 98.8) |
| 2 | Guan XH (2019) | Hubei | 2016-2017 | cohort | q-RT-PCR |  | 155995 | 36-71 months | all | CAMS | unvaccinated and partially vaccinated | full |  | EV-A71 HFMD | 100 |
| 2 | Guan XH (2019) | Hubei | 2016-2017 | cohort | q-RT-PCR |  | 155995 | 6-71 months | all | CAMS | unvaccinated and partially vaccinated | full |  | EV-A71 HFMD hospitalisation | 93.6 (93.2, 93.9) |
| 2 | Guan XH (2019) | Hubei | 2016-2017 | cohort | q-RT-PCR |  | 155995 | 6-35 months | all | CAMS | unvaccinated and partially vaccinated | full |  | EV-A71 HFMD hospitalisation | 89.2 (18.7, 98.6) |
| 2 | Guan XH (2019) | Hubei | 2016-2017 | cohort | q-RT-PCR |  | 155995 | 36-71 months | all | CAMS | unvaccinated and partially vaccinated | full |  | EV-A71 HFMD hospitalisation | 100 |
| 2 | Guan XH (2019) | Hubei | 2016-2017 | cohort | q-RT-PCR |  | 155995 | 6-71 months | all | CAMS | unvaccinated and partially vaccinated | full |  | severe EV-A71 HFMD | 100 |
| 2 | Guan XH (2019) | Hubei | 2016-2017 | cohort | q-RT-PCR |  | 155995 | 6-35 months | all | CAMS | unvaccinated and partially vaccinated | full |  | severe EV-A71 HFMD | 100 |
| 2 | Guan XH (2019) | Hubei | 2016-2017 | cohort | q-RT-PCR |  | 155995 | 36-71 months | all | CAMS | unvaccinated and partially vaccinated | full |  | severe EV-A71 HFMD | 100 |
| 3 | Jiang LN (2019) | Guangxi | 2017-2018 | TND | RT-PCR | tested negative for EV-A71 and tested positive for CV-A16 or pan-enterovirus | 2779 | 0-12 years | all | inactivated EV-A71 vaccines | unvaccinated | partial |  | severe EV-A71 HFMD | 81.4 (19.9, 95.7) |
| 3 | Jiang LN (2019) | Guangxi | 2017-2018 | TND | RT-PCR | tested negative for EV-A71 and tested positive for CV-A16 or pan-enterovirus | 2779 | 0-12 years | all | inactivated EV-A71 vaccines | unvaccinated | full |  | severe EV-A71 HFMD | 88.3 (67.7, 95.8) |
| 3 | Jiang LN (2019) | Guangxi | 2017-2018 | TND | RT-PCR | tested negative for EV-A71 and tested positive for CV-A16 or pan-enterovirus | 2779 | 6-36 month | all | inactivated EV-A71 vaccines | unvaccinated | partial |  | severe EV-A71 HFMD | 74.5 (–10.9, 94.2) |
| 3 | Jiang LN (2019) | Guangxi | 2017-2018 | TND | RT-PCR | tested negative for EV-A71 and tested positive for CV-A16 or pan-enterovirus | 2779 | 37-60 months | all | inactivated EV-A71 vaccines | unvaccinated | partial |  | severe EV-A71 HFMD | 100 (−210.7051, 100) |
| 3 | Jiang LN (2019) | Guangxi | 2017-2018 | TND | RT-PCR | tested negative for EV-A71 and tested positive for CV-A16 or pan-enterovirus | 2779 | 6-36 month | all | inactivated EV-A71 vaccines | unvaccinated | full |  | severe EV-A71 HFMD | 86.2 (61.9, 95) |
| 3 | Jiang LN (2019) | Guangxi | 2017-2018 | TND | RT-PCR | tested negative for EV-A71 and tested positive for CV-A16 or pan-enterovirus | 2779 | 37-60 months | all | inactivated EV-A71 vaccines | unvaccinated | full |  | severe EV-A71 HFMD | 100 (−39.11278, 100) |
| 3 | Jiang LN (2019) | Guangxi | 2017-2018 | TND | RT-PCR | tested negative for EV-A71 and tested positive for CV-A16 or pan-enterovirus | 2779 | 6-36 month | all | inactivated EV-A71 vaccines | unvaccinated | at least 1 dose |  | severe EV-A71 HFMD | 83.8 (62.5, 93) |
| 3 | Jiang LN (2019) | Guangxi | 2017-2018 | TND | RT-PCR | tested negative for EV-A71 and tested positive for CV-A16 or pan-enterovirus | 2779 | 37-60 months | all | inactivated EV-A71 vaccines | unvaccinated | at least 1 dose |  | severe EV-A71 HFMD | 100 |
| 4 | Wang XL (2019) | Beijing | 2017 | TND | RT-PCR | tested negative for EV-A71 and tested positive for non-EV-A71 | 2184 | 6-59 months | all | inactivated EV-A71 vaccines | unvaccinated | partial |  | mild EV-A71 HFMD | 69.8 (1.6, 90.7) |
| 4 | Wang XL (2019) | Beijing | 2017 | TND | RT-PCR | tested negative for EV-A71 and tested positive for non-EV-A71 | 2184 | 6-59 months | all | inactivated EV-A71 vaccines | unvaccinated | full |  | mild EV-A71 HFMD | 83.7 (54.9, 94.1) |
| 4 | Wang XL (2019) | Beijing | 2017 | TND | RT-PCR | tested negative for EV-A71 and tested positive for non-EV-A71 | 2184 | 6-35 months | all | inactivated EV-A71 vaccines | unvaccinated | partial |  | mild EV-A71 HFMD | 68.2 (−143.0, 95.8) |
| 4 | Wang XL (2019) | Beijing | 2017 | TND | RT-PCR | tested negative for EV-A71 and tested positive for non-EV-A71 | 2184 | 36-59 months | all | inactivated EV-A71 vaccines | unvaccinated | partial |  | mild EV-A71 HFMD | 69.6 (−29.9, 92.9) |
| 4 | Wang XL (2019) | Beijing | 2017 | TND | RT-PCR | tested negative for EV-A71 and tested positive for non-EV-A71 | 2184 | 6-35 months | all | inactivated EV-A71 vaccines | unvaccinated | full |  | mild EV-A71 HFMD | 77.3 (4.6, 94.6) |
| 4 | Wang XL (2019) | Beijing | 2017 | TND | RT-PCR | tested negative for EV-A71 and tested positive for non-EV-A71 | 2184 | 36-59 months | all | inactivated EV-A71 vaccines | unvaccinated | full |  | mild EV-A71 HFMD | 86.8 (44.6, 96.9) |
| 4 | Wang XL (2019) | Beijing | 2017 | TND | RT-PCR | tested negative for EV-A71 and tested positive for non-EV-A71 | 2184 | 6-59 months | all | inactivated EV-A71 vaccines | unvaccinated | full |  | severe EV-A71 HFMD | 100 (−68.1, 100) |
| **5** | Duan XX (2024) | Chengdu | 2017-2022 | TND | RT-PCR | tested negative for EV-A71 | 4883 | aged 6 months and over | all | inactivated EV-A71 vaccines | unvaccinated | partial |  | EV-A71 HFMD | 51.7 (−20.9, 80.7) |
| **5** | Duan XX (2024) | Chengdu | 2017-2022 | TND | RT-PCR | tested negative for EV-A71 | 4883 | aged 6 months and over | all | inactivated EV-A71 vaccines | unvaccinated | full |  | EV-A71 HFMD | 63.4 (35.2, 79.4) |
| **5** | Duan XX (2024) | Chengdu | 2017-2022 | TND | RT-PCR | tested negative for EV-A71 | 4883 | aged 6 months and over | all | inactivated EV-A71 vaccines | unvaccinated | partial |  | CVA-16 HFMD | 37.7 (−4.7, 62.9) |
| **5** | Duan XX (2024) | Chengdu | 2017-2022 | TND | RT-PCR | tested negative for EV-A71 | 4883 | aged 6 months and over | all | inactivated EV-A71 vaccines | unvaccinated | full |  | CVA-16 HFMD | 11.5 (−15.9, 32.4) |
| **5** | Duan XX (2024) | Chengdu | 2017-2022 | TND | RT-PCR | tested negative for EV-A71 | 4883 | aged 6 months and over | all | inactivated EV-A71 vaccines | unvaccinated | partial |  | CVA-10 HFMD | −26.0 (−113.8, 25.7) |
| **5** | Duan XX (2024) | Chengdu | 2017-2022 | TND | RT-PCR | tested negative for EV-A71 | 4883 | aged 6 months and over | all | inactivated EV-A71 vaccines | unvaccinated | full |  | CVA-10 HFMD | −24.8 (−73.2, 10.1) |
| **5** | Duan XX (2024) | Chengdu | 2017-2022 | TND | RT-PCR | tested negative for EV-A71 | 4883 | aged 6 months and over | all | inactivated EV-A71 vaccines | unvaccinated | partial |  | CVA-6 HFMD | −17.2 (−60.5, 14.4) |
| **5** | Duan XX (2024) | Chengdu | 2017-2022 | TND | RT-PCR | tested negative for EV-A71 | 4883 | aged 6 months and over | all | inactivated EV-A71 vaccines | unvaccinated | full |  | CVA-6 HFMD | −3.7 (−24.7, 13.8) |
| **5** | Duan XX (2024) | Chengdu | 2017-2022 | TND | RT-PCR | tested negative for EV-A71 | 4883 | aged 6 months and over | male | inactivated EV-A71 vaccines | unvaccinated | partial |  | EV-A71 HFMD | 67.9 (−34.2, 92.3) |
| **5** | Duan XX (2024) | Chengdu | 2017-2022 | TND | RT-PCR | tested negative for EV-A71 | 4883 | aged 6 months and over | female | inactivated EV-A71 vaccines | unvaccinated | partial |  | EV-A71 HFMD | 29.9 (−135.4, 79.1) |
| **5** | Duan XX (2024) | Chengdu | 2017-2022 | TND | RT-PCR | tested negative for EV-A71 | 4883 | aged 6 months and 2 years | all | inactivated EV-A71 vaccines | unvaccinated | partial |  | EV-A71 HFMD | −2.7 (−170.7, 61.1) |
| **5** | Duan XX (2024) | Chengdu | 2017-2022 | TND | RT-PCR | tested negative for EV-A71 | 4883 | aged 2 years and over | all | inactivated EV-A71 vaccines | unvaccinated | partial |  | EV-A71 HFMD | 100 (40.66954, 100) |
| **5** | Duan XX (2024) | Chengdu | 2017-2022 | TND | RT-PCR | tested negative for EV-A71 | 4883 | aged 6 months and over | all | inactivated EV-A71 vaccines | unvaccinated | partial |  | severe EV-A71 HFMD | 16.6 (−1013, 93.7) |
| **5** | Duan XX (2024) | Chengdu | 2017-2022 | TND | RT-PCR | tested negative for EV-A71 | 4883 | aged 6 months and over | all | inactivated EV-A71 vaccines | unvaccinated | partial |  | mild EV-A71 HFMD | 58.6 (−34.2, 87.2) |
| **5** | Duan XX (2024) | Chengdu | 2017-2022 | TND | RT-PCR | tested negative for EV-A71 | 4883 | aged 6 months and over | all | inactivated EV-A71 vaccines | unvaccinated | partial | 14 days to 6 months | EV-A71 HFMD | 49.9 (−62.0, 84.5) |
| **5** | Duan XX (2024) | Chengdu | 2017-2022 | TND | RT-PCR | tested negative for EV-A71 | 4883 | aged 6 months and over | all | inactivated EV-A71 vaccines | unvaccinated | partial | 6 months and over | EV-A71 HFMD | 56.8 (−219.9, 94.2) |
| **5** | Duan XX (2024) | Chengdu | 2017-2022 | TND | RT-PCR | tested negative for EV-A71 | 4883 | First dose vaccination age < 12 months | all | inactivated EV-A71 vaccines | unvaccinated | partial |  | EV-A71 HFMD | 44.3 (−131.6, 86.6) |
| **5** | Duan XX (2024) | Chengdu | 2017-2022 | TND | RT-PCR | tested negative for EV-A71 | 4883 | First dose vaccination age >= 12 months | all | inactivated EV-A71 vaccines | unvaccinated | partial |  | EV-A71 HFMD | 82.6 (−26.4, 97.6) |
| **5** | Duan XX (2024) | Chengdu | 2017-2022 | TND | RT-PCR | tested negative for EV-A71 | 4883 | aged 6 months and over | male | inactivated EV-A71 vaccines | unvaccinated | full |  | EV-A71 HFMD | 79.4 (41.6, 92.7) |
| **5** | Duan XX (2024) | Chengdu | 2017-2022 | TND | RT-PCR | tested negative for EV-A71 | 4883 | aged 6 months and over | female | inactivated EV-A71 vaccines | unvaccinated | full |  | EV-A71 HFMD | 48.7 (−5.4, 75) |
| **5** | Duan XX (2024) | Chengdu | 2017-2022 | TND | RT-PCR | tested negative for EV-A71 | 4883 | aged 6 months to 2 years | all | inactivated EV-A71 vaccines | unvaccinated | full |  | EV-A71 HFMD | 50.8 (−14.9, 78.9) |
| **5** | Duan XX (2024) | Chengdu | 2017-2022 | TND | RT-PCR | tested negative for EV-A71 | 4883 | aged 2 years and over | all | inactivated EV-A71 vaccines | unvaccinated | full |  | EV-A71 HFMD | 69.2 (32.8, 85.9) |
| **5** | Duan XX (2024) | Chengdu | 2017-2022 | TND | RT-PCR | tested negative for EV-A71 | 4883 | aged 6 months and over | all | inactivated EV-A71 vaccines | unvaccinated | full |  | severe EV-A71 HFMD | 100 (76.4768, 100) |
| **5** | Duan XX (2024) | Chengdu | 2017-2022 | TND | RT-PCR | tested negative for EV-A71 | 4883 | aged 6 months and over | all | inactivated EV-A71 vaccines | unvaccinated | full |  | mild EV-A71 HFMD | 51.6 (11.2, 73.6) |
| **5** | Duan XX (2024) | Chengdu | 2017-2022 | TND | RT-PCR | tested negative for EV-A71 | 4883 | aged 6 months and over | all | inactivated EV-A71 vaccines | unvaccinated | full | 14 days to 6 months | EV-A71 HFMD | 68 (19.8, 87.2) |
| **5** | Duan XX (2024) | Chengdu | 2017-2022 | TND | RT-PCR | tested negative for EV-A71 | 4883 | aged 6 months and over | all | inactivated EV-A71 vaccines | unvaccinated | full | 6 months and over | EV-A71 HFMD | 55.6 (13.1, 77.3) |
| **5** | Duan XX (2024) | Chengdu | 2017-2022 | TND | RT-PCR | tested negative for EV-A71 | 4883 | First dose vaccination age < 12 months | all | inactivated EV-A71 vaccines | unvaccinated | full |  | EV-A71 HFMD | 68.6 (30.9, 85.7) |
| **5** | Duan XX (2024) | Chengdu | 2017-2022 | TND | RT-PCR | tested negative for EV-A71 | 4883 | First dose vaccination age >= 12 months | all | inactivated EV-A71 vaccines | unvaccinated | full |  | EV-A71 HFMD | 49.5 (−1.9, 75) |
| 6 | Hua RJ (2021) | Shanghai | 2017-2017 | cohort | PCR |  | 3018 | 8-20 months | all | CAMS | unvaccinated | full |  | EV-A71 HFMD | 100 (-66.99, 100) |
| 7 | Zhang YT (2024) | Henan, Hunan, and Yunnan | 2019 | TND | RT-PCR | tested negative for EV-A71 | 3223 | 6-71 months | all | inactivated EV-A71 vaccines | unvaccinated | partial |  | EV-A71 HFMD | 90.1 (55.4, 99.4) |
| 7 | Zhang YT (2024) | Henan, Hunan, and Yunnan | 2019 | TND | RT-PCR | tested negative for EV-A71 | 3223 | 6-71 months | all | inactivated EV-A71 vaccines | unvaccinated | full |  | EV-A71 HFMD | 90.9 (80.1, 96.8) |
| 7 | Zhang YT (2024) | Henan, Hunan, and Yunnan | 2019 | TND | RT-PCR | tested negative for EV-A71 | 3223 | 6-35 months | all | inactivated EV-A71 vaccines | unvaccinated | partial |  | EV-A71 HFMD | 86.5 (38.3, 99.2) |
| 7 | Zhang YT (2024) | Henan, Hunan, and Yunnan | 2019 | TND | RT-PCR | tested negative for EV-A71 | 3223 | 6-35 months | all | inactivated EV-A71 vaccines | unvaccinated | full |  | EV-A71 HFMD | 89.1 (73.9, 96.7) |
| 7 | Zhang YT (2024) | Henan, Hunan, and Yunnan | 2019 | TND | RT-PCR | tested negative for EV-A71 | 3223 | 36-71 months | all | inactivated EV-A71 vaccines | unvaccinated | partial |  | EV-A71 HFMD | 100 (−59.67438, 100) |
| 7 | Zhang YT (2024) | Henan, Hunan, and Yunnan | 2019 | TND | RT-PCR | tested negative for EV-A71 | 3223 | 36-71 months | all | inactivated EV-A71 vaccines | unvaccinated | full |  | EV-A71 HFMD | 94.8 (75.7, 99.7) |
| 7 | Zhang YT (2024) | Henan, Hunan, and Yunnan | 2019 | TND | RT-PCR | tested negative for EV-A71 | 3223 | 6-71 months | all | inactivated EV-A71 vaccines | unvaccinated | partial |  | mild EV-A71 HFMD | 86.2 (37.3, 99.2) |
| 7 | Zhang YT (2024) | Henan, Hunan, and Yunnan | 2019 | TND | RT-PCR | tested negative for EV-A71 | 3223 | 6-71 months | all | inactivated EV-A71 vaccines | unvaccinated | full |  | mild EV-A71 HFMD | 86.4 (69.8, 95.2) |
| 7 | Zhang YT (2024) | Henan, Hunan, and Yunnan | 2019 | TND | RT-PCR | tested negative for EV-A71 | 3223 | 6-71 months | all | inactivated EV-A71 vaccines | unvaccinated | partial |  | severe EV-A71 HFMD | 100 (−42.35494, 100) |
| 7 | Zhang YT (2024) | Henan, Hunan, and Yunnan | 2019 | TND | RT-PCR | tested negative for EV-A71 | 3223 | 6-71 months | all | inactivated EV-A71 vaccines | unvaccinated | full |  | severe EV-A71 HFMD | 100 (78.46925, 100) |
| 8 | Wang J (2023) | Shanghai | 2011-2021 | case-control | RT-PCR | tested postive for other EV | 487 | 2 months to 26 years old | all | inactivated EV-A71 vaccines | unvaccinated and partially vaccinated | full |  | EV-A71 HFMD | 48 (-130, 88) |
| 9 | Zhu F (2014) | Jiangsu | 2012 | RCT | PCT or viral isolation | unvaccinated | 10077 | 6 to 35 months | all | Sinovac | unvaccinated | full | 12 months post-vaccination | EV-A71 HFMD | 94.6 (86.6, 97.8) |
| 9 | Zhu F (2014) | Jiangsu | 2012 | RCT | PCT or viral isolation | unvaccinated | 10077 | 6 to 35 months | all | Sinovac | unvaccinated | full | 12 months post-vaccination | EV-A71 herpangina | 100 (-48.4, 100) |
| 9 | Zhu F (2014) | Jiangsu | 2012 | RCT | PCT or viral isolation | unvaccinated | 10077 | 6 to 35 months | all | Sinovac | unvaccinated | full | 12 months post-vaccination | EV-A71 hospitalization | 100 (83.7, 100) |
| 9 | Zhu F (2014) | Jiangsu | 2012 | RCT | PCT or viral isolation | unvaccinated | 10077 | 6 to 35 months | all | Sinovac | unvaccinated | full | 12 months post-vaccination | severe EV-A71 HFMD | 100 (42.6, 100) |
| 9 | Zhu F (2014) | Jiangsu | 2012 | RCT | PCT or viral isolation | unvaccinated | 10077 | 6 to 35 months | all | Sinovac | unvaccinated | full | 12 months post-vaccination | EV-A71 diseases | 88 (78.6, 93.2) |
| 9 | Zhu F (2014) | Jiangsu | 2012 | RCT | PCT or viral isolation | unvaccinated | 10077 | 6 to 35 months | all | Sinovac | unvaccinated | full | 6 months post-vaccination | EV-A71 HFMD | 97.4 (89.5, 99.4) |
| 9 | Zhu F (2014) | Jiangsu | 2012 | RCT | PCT or viral isolation | unvaccinated | 10077 | 6 to 35 months | all | Sinovac | unvaccinated | full | 6 months post-vaccination | EV-A71 herpangina | 100 (-49, 100) |
| 9 | Zhu F (2014) | Jiangsu | 2012 | RCT | PCT or viral isolation | unvaccinated | 10077 | 6 to 35 months | all | Sinovac | unvaccinated | full | 6 months post-vaccination | EV-A71 hospitalization | 100 (83.7, 100) |
| 9 | Zhu F (2014) | Jiangsu | 2012 | RCT | PCT or viral isolation | unvaccinated | 10077 | 6 to 35 months | all | Sinovac | unvaccinated | full | 6 months post-vaccination | severe EV-A71 HFMD | 100 (42.4, 100) |
| 9 | Zhu F (2014) | Jiangsu | 2012 | RCT | PCT or viral isolation | unvaccinated | 10077 | 6 to 35 months | all | Sinovac | unvaccinated | full | 6 months post-vaccination | EV-A71 diseases | 89.3 (79.5, 94.4) |
| 10 | Li JX (2016) | Jiangsu | 2012.1-2013.3 | RCT | PCT or viral isolation | unvaccinated | 10077 | 6 to 35 months | all | Sinovac | unvaccinated | full | 26 months post-vaccination | EV-A71 HFMD | 94.7 (87.8, 97.6) |
| 10 | Li JX (2016) | Jiangsu | 2012.1-2013.3 | RCT | PCT or viral isolation | unvaccinated | 10077 | 6 to 35 months | all | Sinovac | unvaccinated | full | 15-26 months post-vaccination | EV-A71 HFMD | 95.1 (63.6, 99.3) |
| 11 | Zhu FC (2013) | Jiangsu,Beijing | 2012-2013 | RCT | PCR | unvaccinated | 10245 | 6 to 35 months | all | Vigoo | unvaccinated | full | 12 months post-vaccination | EV-A71 HFMD | 90 (67.1, 96.9) |
| 11 | Zhu FC (2013) | Jiangsu,Beijing | 2012-2013 | RCT | PCR | unvaccinated | 10245 | 6 to 35 months | all | Vigoo | unvaccinated | full | 12 months post-vaccination | EV-A71 diseases | 80.4 (58.2, 90.8) |
| 11 | Zhu FC (2013) | Jiangsu,Beijing | 2012-2013 | RCT | PCR | unvaccinated | 10245 | 6 to 35 months | all | Vigoo | unvaccinated | full | 12 months post-vaccination | EV-A71 non-HFMD | 54.3 (-31.4, 84.1) |
| 12 | Wei MW (2017) | Jiangsu,Beijing | 2012-2013 | RCT | PCR | unvaccinated | 10245 | 6 to 35 months | all | Vigoo | unvaccinated | full | 26 months post-vaccination | EV-A71 HFMD | 94.8 (83.5, 98.4) |
| 12 | Wei MW (2017) | Jiangsu,Beijing | 2012-2013 | RCT | PCR | unvaccinated | 10245 | 6 to 35 months | all | Vigoo | unvaccinated | full | 15-26 months post-vaccination | EV-A71 HFMD | 100 (84.15, 100) |
| 13 | Li R (2014) | Guangxi | 2012.3-2013.2 | RCT | PCR | unvaccinated | 12000 | 6 to 71 months | all | CAMS | unvaccinated | full | 12 months post-vaccination | EV-A71 HFMD | 97.4 (92.9, 99) |
| 13 | Li R (2014) | Guangxi | 2012.3-2013.2 | RCT | PCR | unvaccinated | 12000 | 6 to 23 months | all | CAMS | unvaccinated | full | 12 months post-vaccination | EV-A71 HFMD | 97.9 (91.4, 99.5) |
| 13 | Li R (2014) | Guangxi | 2012.3-2013.2 | RCT | PCR | unvaccinated | 12000 | 24 to 71 months | all | CAMS | unvaccinated | full | 12 months post-vaccination | EV-A71 HFMD | 96.5 (85.6, 99.1) |
| 14 | Nguyen TT (2022) | Taiwan,Vietnam | 2019.4-2019.12 | RCT | CODEHOP assays, PCR or viral isolation | unvaccinated | 3049 | 2–71 months | all | MVC | unvaccinated | full | 26 months post-vaccination | EV-A71 diseases | 96.8 (85.5, 100) |
| 14 | Nguyen TT (2022) | Taiwan,Vietnam | 2019.4-2019.12 | RCT | CODEHOP assays, PCR or viral isolation | unvaccinated | 3049 | 2–71 months | all | MVC | unvaccinated | full | 26 months post-vaccination | EV-A71 hospitalization | 81 (-12.3, 100) |

Note. Evaluation of title, abstract and full text were screened separately by Yan XM, Liu YH, and Chen FK, and disagreements were resolved by discussion. Essential variables of each article included in the systematic review were extracted by Yan XM, and checked by Liu YH and Chen FK. The data was extracted in April and May 2024.
