# Supplementary material for: The efficacy and effectiveness of enterovirus A71 vaccines against hand, foot, and mouth disease: A systematic review and meta-analysis
Source: PLoS One. 2025 May 22;20(5):e0323782. doi: 10.1371/journal.pone.0323782 (PMC12097632; doi:10.1371/journal.pone.0323782)
Supplement: S5 Table — (DOCX) [file pone.0323782.s005.docx]

**Supporting information**

**The efficacy and effectiveness of enterovirus A71 vaccines against hand, foot, and mouth disease: a systematic review and meta-analysis**

# **S5 Table. Quality assessment for observational studies according to ROBINS-I**

| **Study** | **Pre-intervention and at-intervention domains** | | | **Post-intervention domains** | | | | **Overall bias** |
| --- | --- | --- | --- | --- | --- | --- | --- | --- |
|  | **Bias due to confounding** | **Bias in selection of participants into the study** | **Bias in classification of interventions** | **Bias due to deviations from intended interventions** | **Bias due to missing data** | **Bias in measurement of outcomes** | **Bias in selection of the reported result** |  |
| Li Y | Moderate risk | Low risk | Low risk | Low risk | Low risk | Low risk | Low risk | Moderate risk |
| Jiang LN | Moderate risk | Low risk | Low risk | Low risk | Low risk | Low risk | Low risk | Moderate risk |
| Wang XL | Moderate risk | Low risk | Low risk | Low risk | Low risk | Low risk | Low risk | Moderate risk |
| Duan XX | Moderate risk | Low risk | Moderate risk | Low risk | Low risk | Low risk | Low risk | Moderate risk |
| Zhang YT | Moderate risk | Low risk | Low risk | Low risk | Low risk | Low risk | Low risk | Moderate risk |
| Wang J | Moderate risk | Low risk | Moderate risk | Low risk | Low risk | Low risk | Low risk | Moderate risk |
| Guan XH | Moderate risk | Low risk | Low risk | Low risk | Low risk | Low risk | Moderate risk | Moderate risk |
| Hua RJ | Moderate risk | Low risk | Low risk | Low risk | Low risk | Low risk | Low risk | Moderate risk |

Note: Moderate risk of overall bias refers to study is judged to be at low or moderate risk of bias for all domains. Low risk of overall bias refers to study is judged to be at low risk of bias for all domains
