# Supplementary material for: The efficacy and effectiveness of enterovirus A71 vaccines against hand, foot, and mouth disease: A systematic review and meta-analysis
Source: PLoS One. 2025 May 22;20(5):e0323782. doi: 10.1371/journal.pone.0323782 (PMC12097632; doi:10.1371/journal.pone.0323782)
Supplement: S6 Table — (DOCX) [file pone.0323782.s006.docx]

**Supporting information**

**The efficacy and effectiveness of enterovirus A71 vaccines against hand, foot, and mouth disease: a systematic review and meta-analysis**

**S6 Table. Diagnostic checks for assumptions of random-effects model**

| Dose | Group | Study No | Normality test | | Heterogeneity test | | | Publication Bias test | |
| --- | --- | --- | --- | --- | --- | --- | --- | --- | --- |
|  |  |  | W | P | I^2^ | tua^2^ | P | tau | P |
| full | non-severe | 6 | 0.94 | 0.64 | 62.80 | 0.27 | 0.07 | -0.07 | 1.00 |
| partial | non-severe | 5 | 0.85 | 0.21 | 0.00 | 0.00 | 0.58 | -1.00 | 0.02 |
| full | severe | 3 | 0.85 | 0.25 | 0.00 | 0.00 | 0.73 | -0.33 | 1.00 |
| partial | severe | 3 | 0.85 | 0.25 | 0.00 | 0.00 | 0.93 | -0.33 | 1.00 |
| full | any | 5 | 0.91 | 0.45 | 0.00 | 0.08 | 0.42 | 0.00 | 1.00 |
| partial | any | 5 | 0.91 | 0.45 | 0.00 | 0.00 | 0.82 | 0.00 | 1.00 |
| full | young | 4 | 0.92 | 0.54 | 32.10 | 0.16 | 0.22 | 0.00 | 1.00 |
| partial | young | 4 | 0.92 | 0.54 | 19.60 | 0.24 | 0.29 | 0.00 | 1.00 |
| full | old | 4 | 0.95 | 0.70 | 0.00 | 0.00 | 0.78 | -0.67 | 0.33 |
| partial | old | 4 | 0.95 | 0.70 | 0.00 | 0.00 | 0.87 | -0.67 | 0.33 |
| full | 12-month | 3 | 0.99 | 0.77 | 31.90 | 0.11 | 0.23 | 0.33 | 1.00 |
| full | 15-month | 2 | - | - | 0.00 | 0.00 | 0.59 | -1.00 | 1.00 |
| full | 26-month | 2 | - | - | 0.00 | 0.00 | 0.94 | -1.00 | 1.00 |
