# Supplementary figures and images for: The efficacy and effectiveness of enterovirus A71 vaccines against hand, foot, and mouth disease: A systematic review and meta-analysis
Source: PLoS One. 2025 May 22;20(5):e0323782. doi: 10.1371/journal.pone.0323782 (PMC12097632; doi:10.1371/journal.pone.0323782)

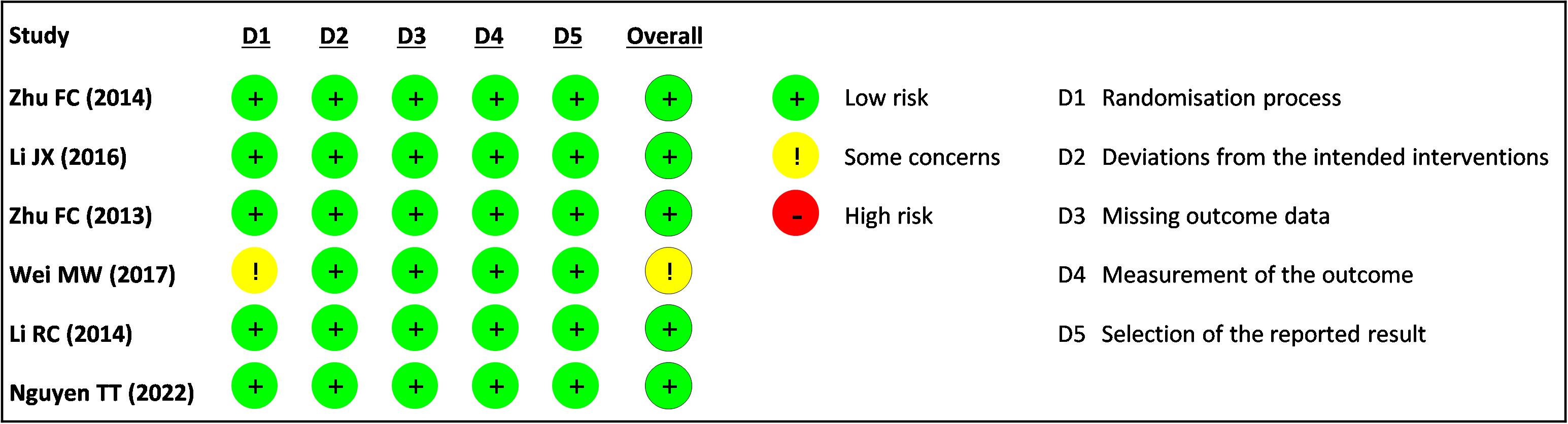

Supplement: S1 Fig — (TIF) [file pone.0323782.s007.tif]

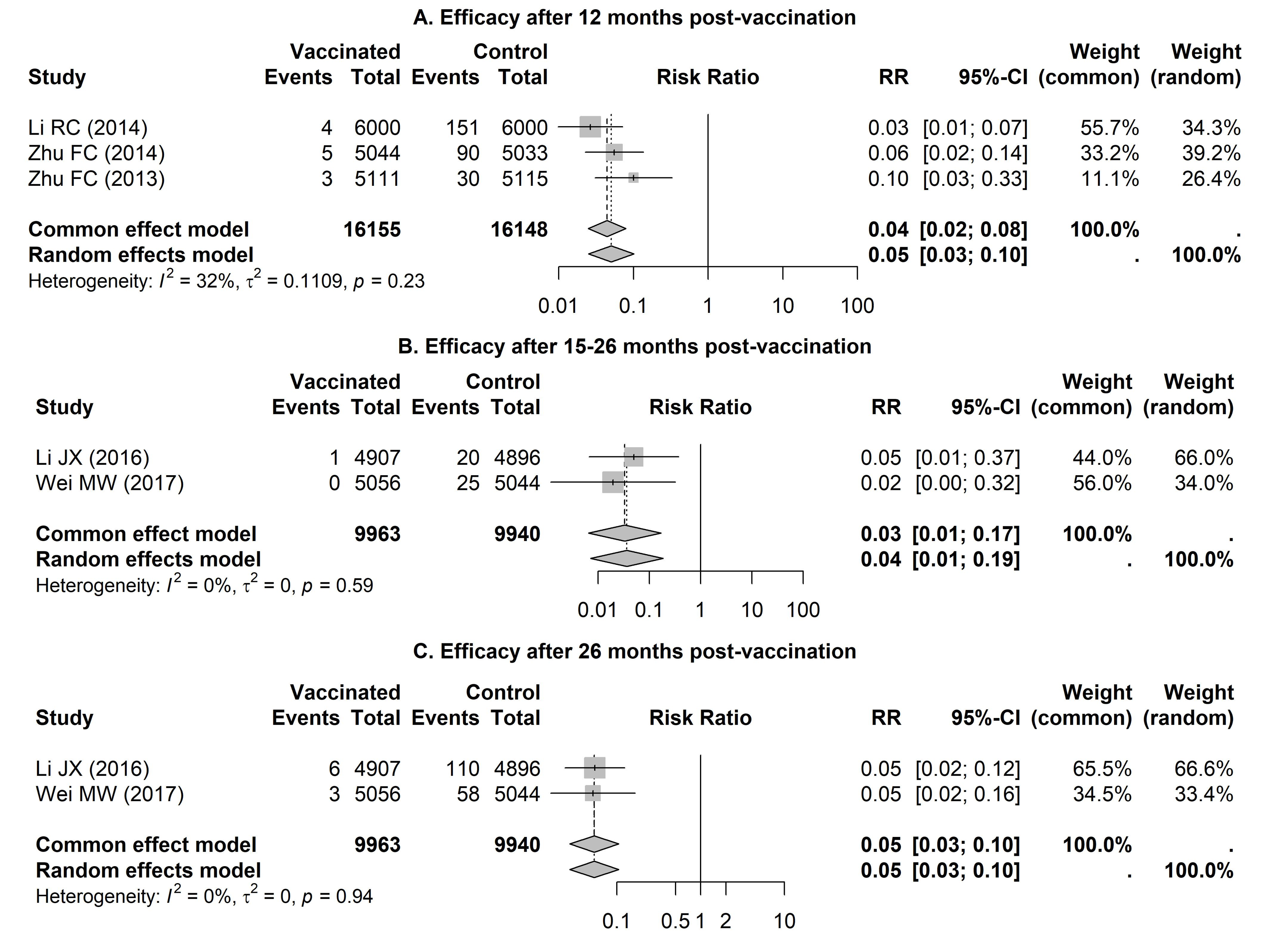

Supplement: S2 Fig — (TIF) [file pone.0323782.s008.tif]

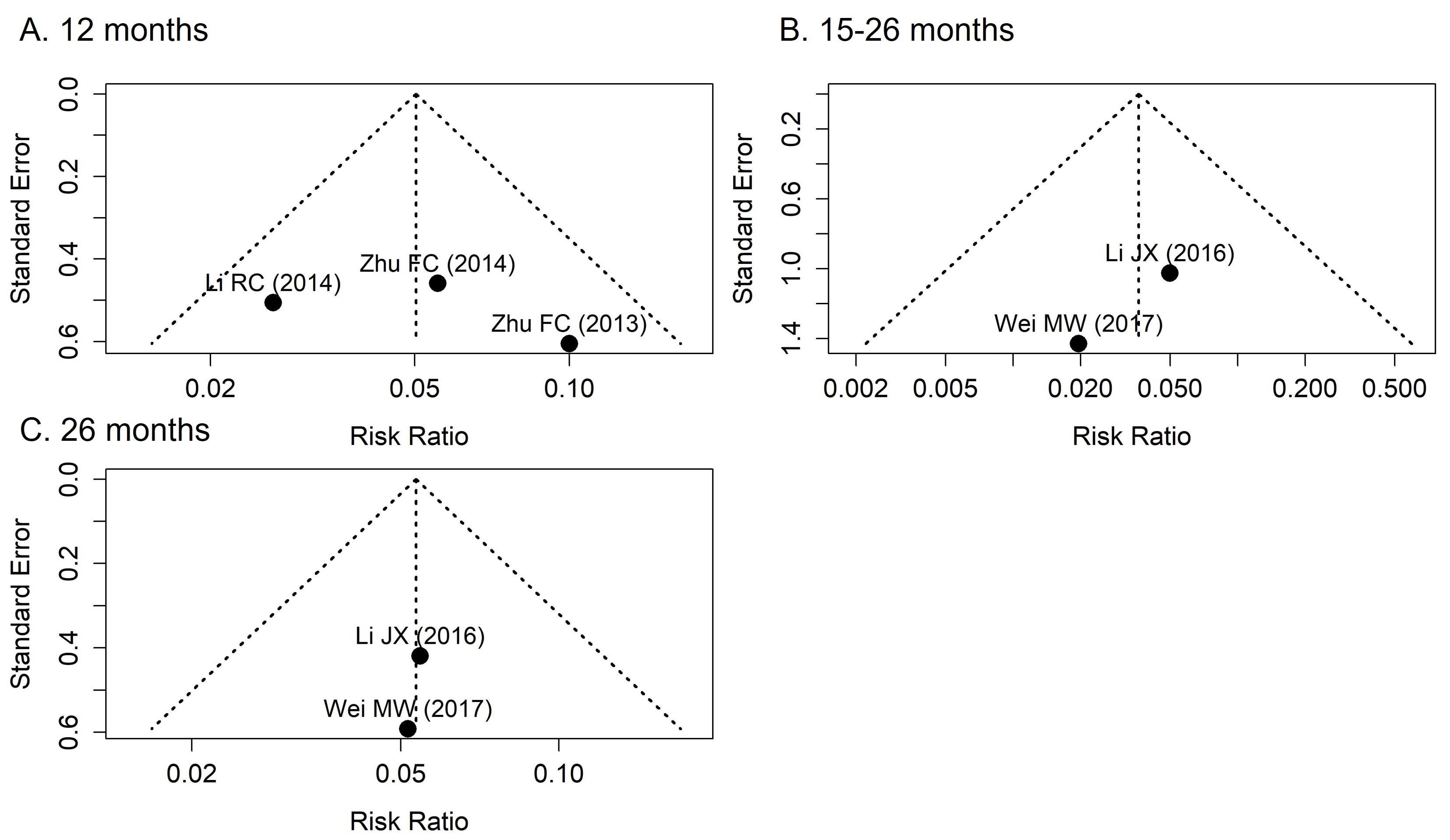

Supplement: S3 Fig — (TIF) [file pone.0323782.s009.tif]

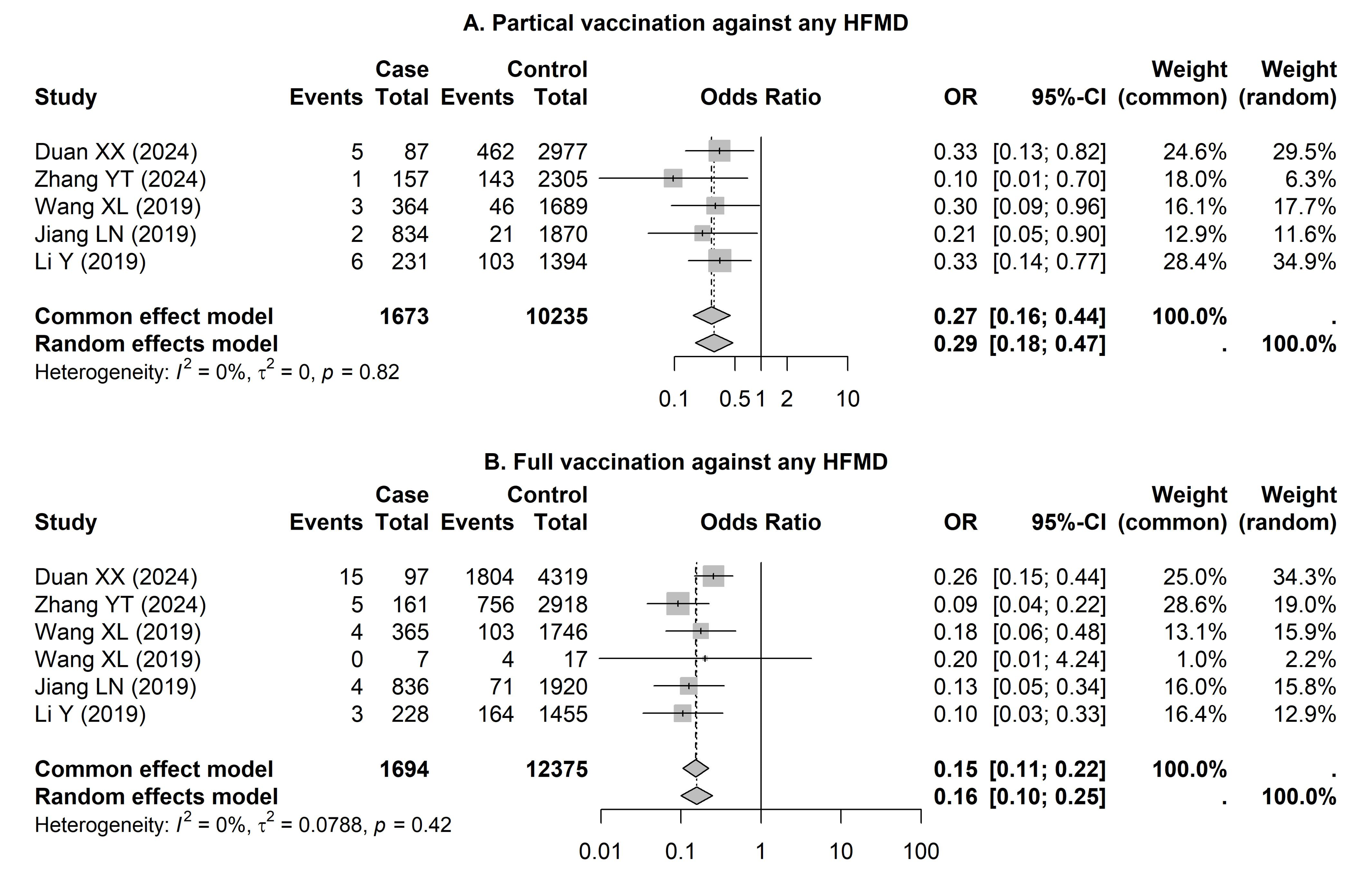

Supplement: S4 Fig — (TIF) [file pone.0323782.s010.tif]

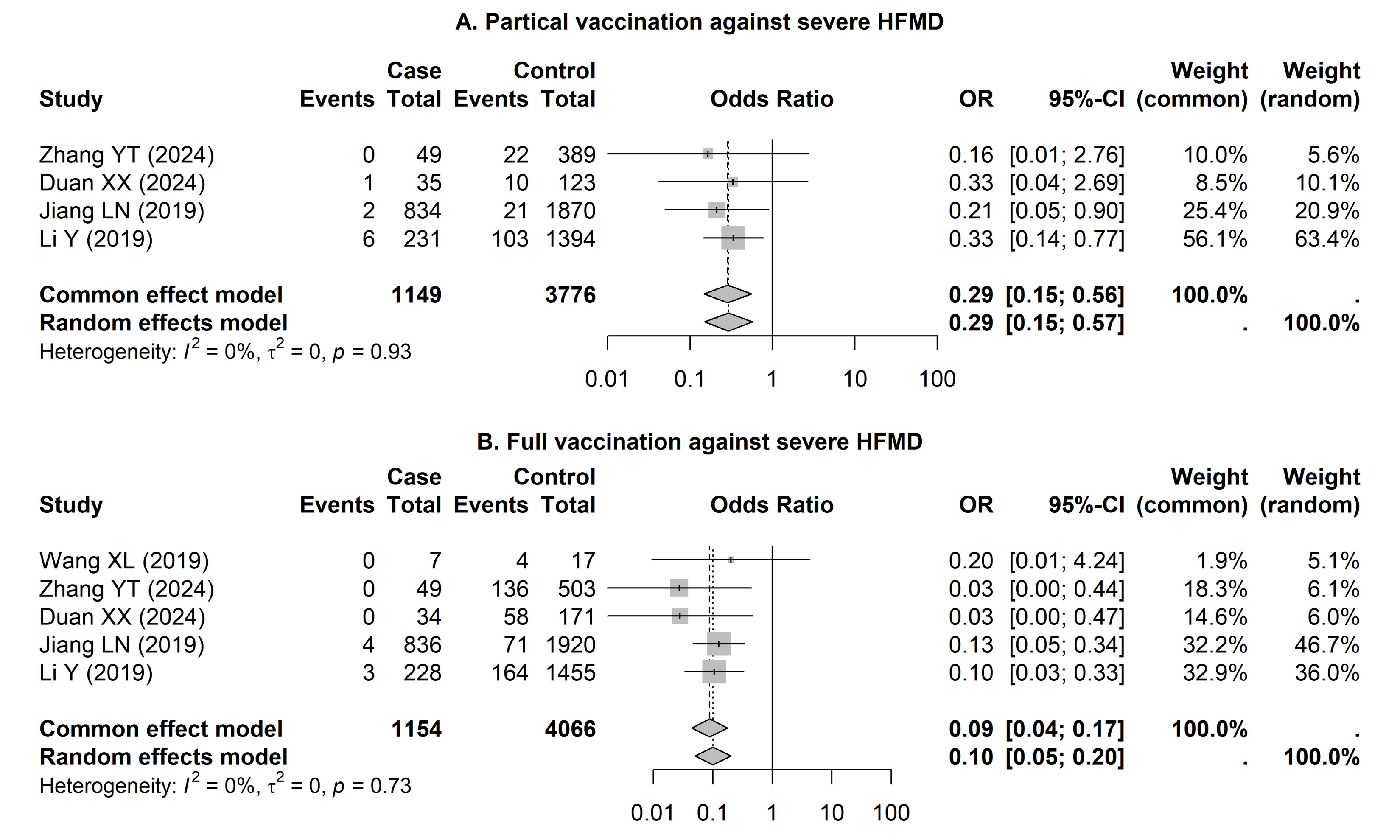

Supplement: S5 Fig — (TIF) [file pone.0323782.s011.tif]

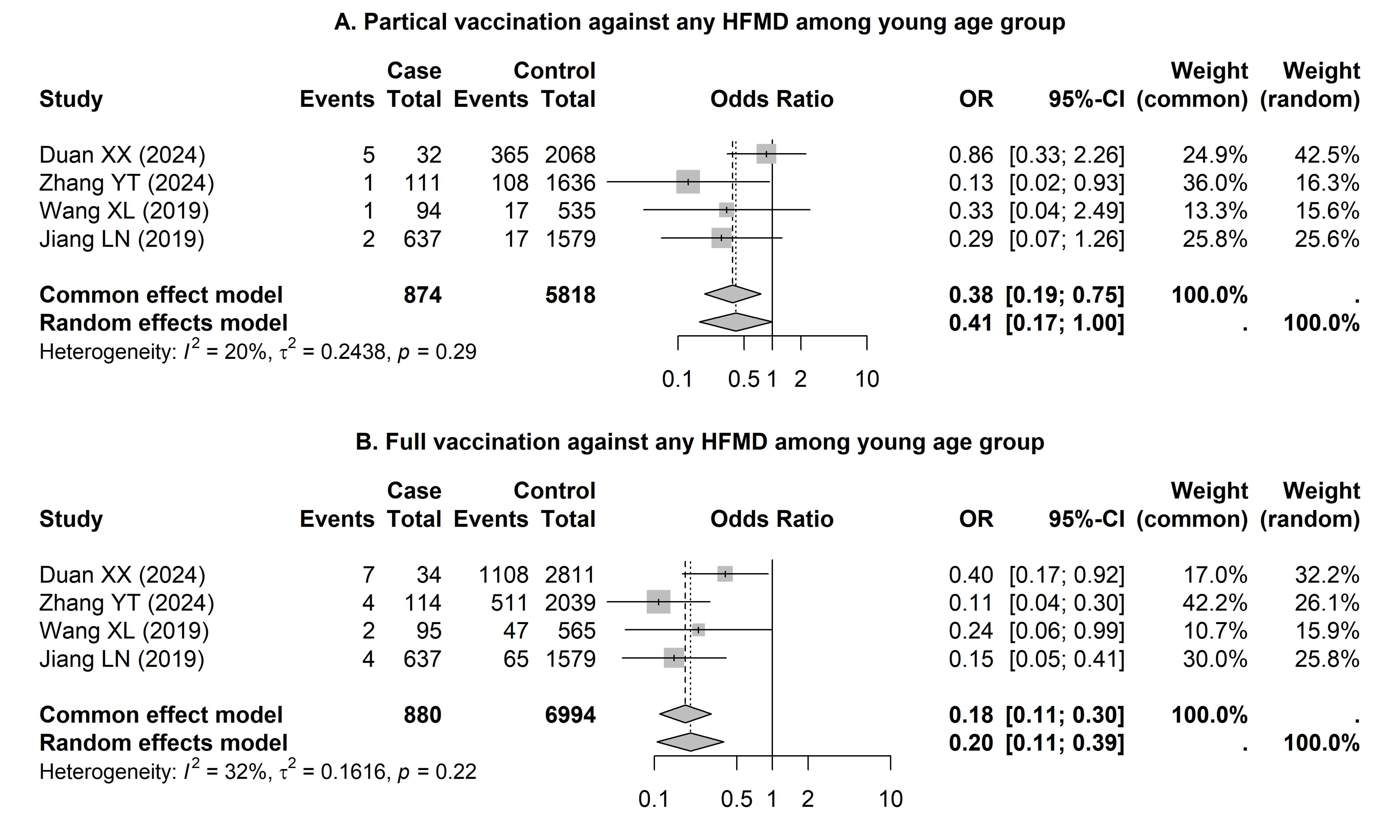

Supplement: S7 Fig — (TIF) [file pone.0323782.s013.tif]

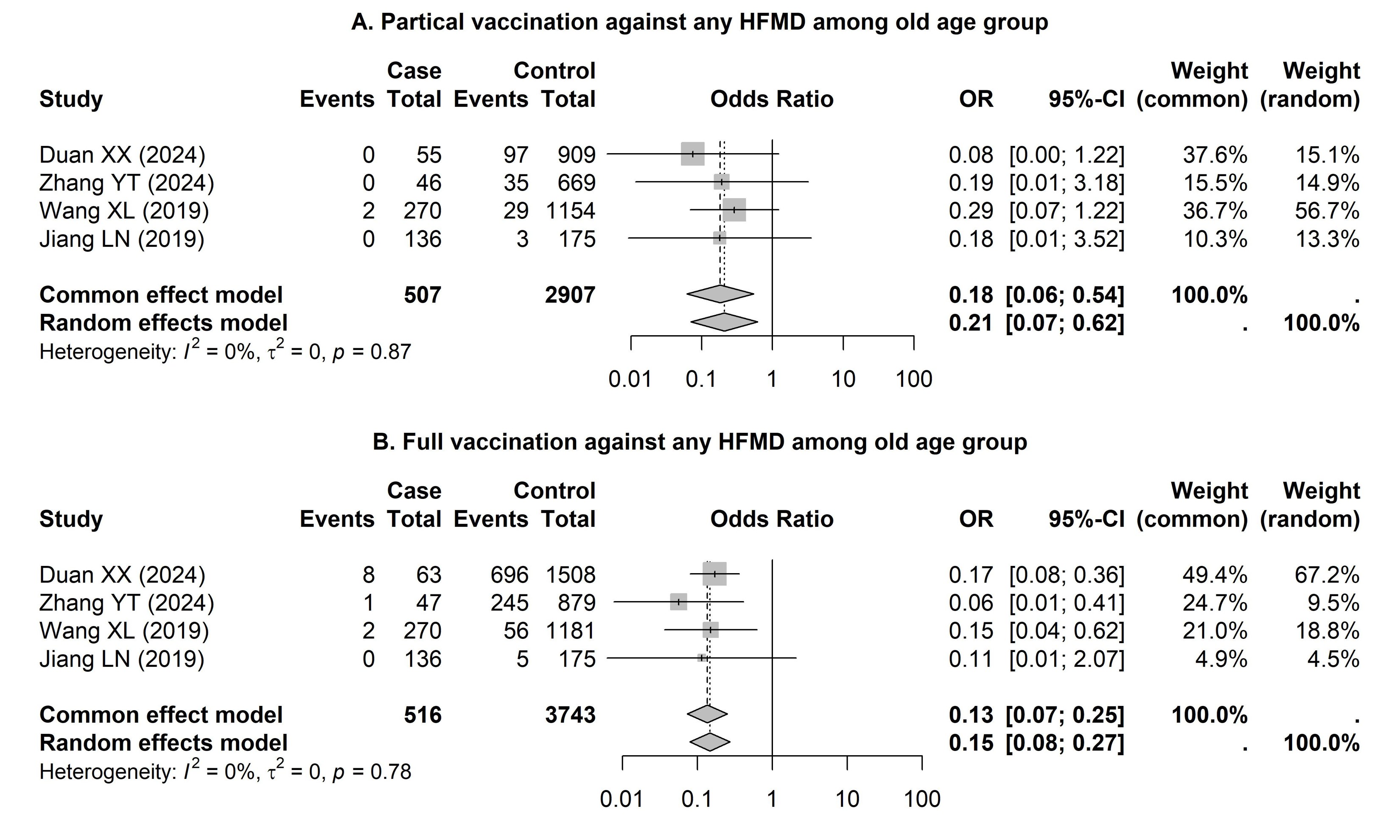

Supplement: S8 Fig — (TIF) [file pone.0323782.s014.tif]

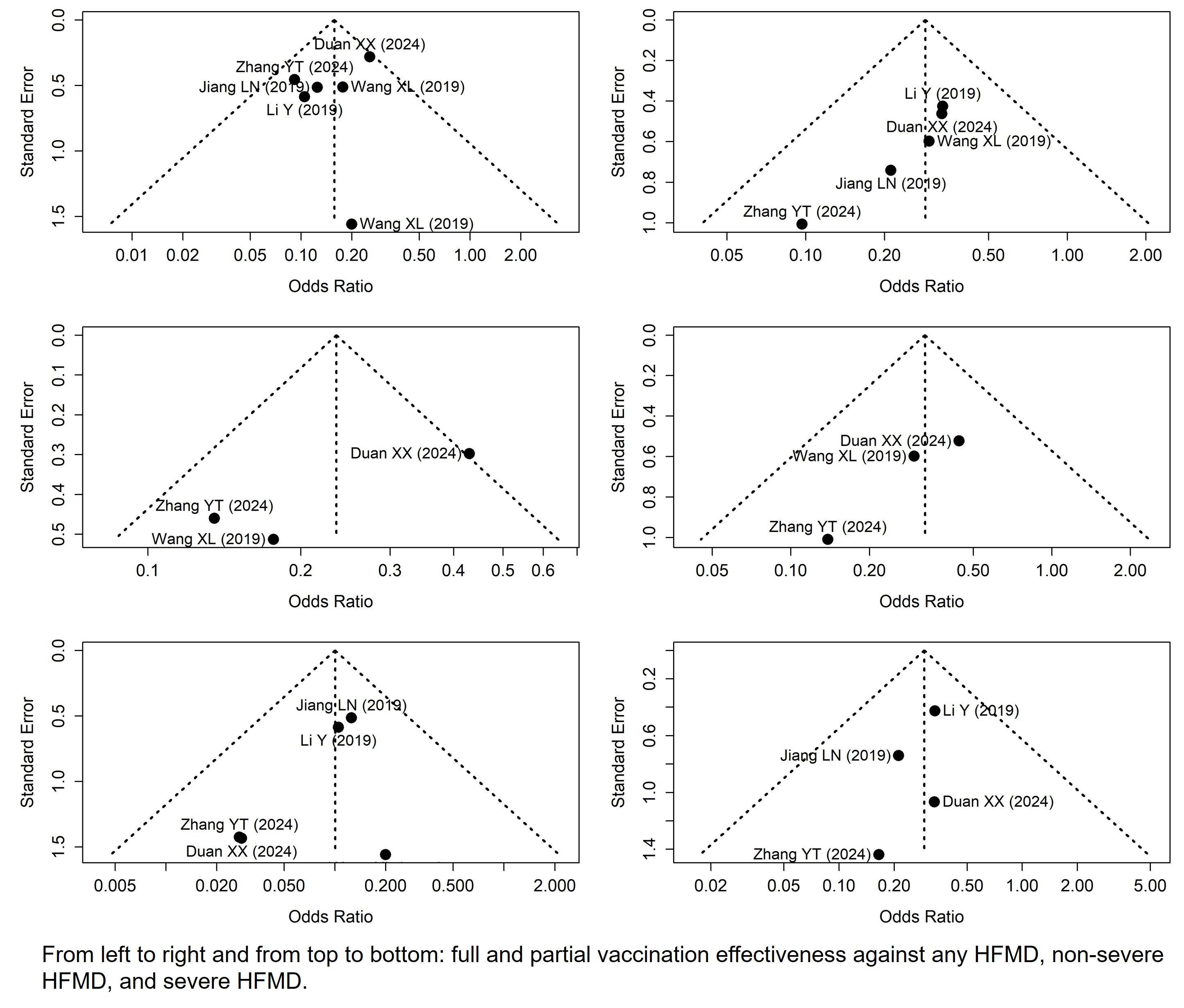

Supplement: S9 Fig — (TIF) [file pone.0323782.s015.tif]

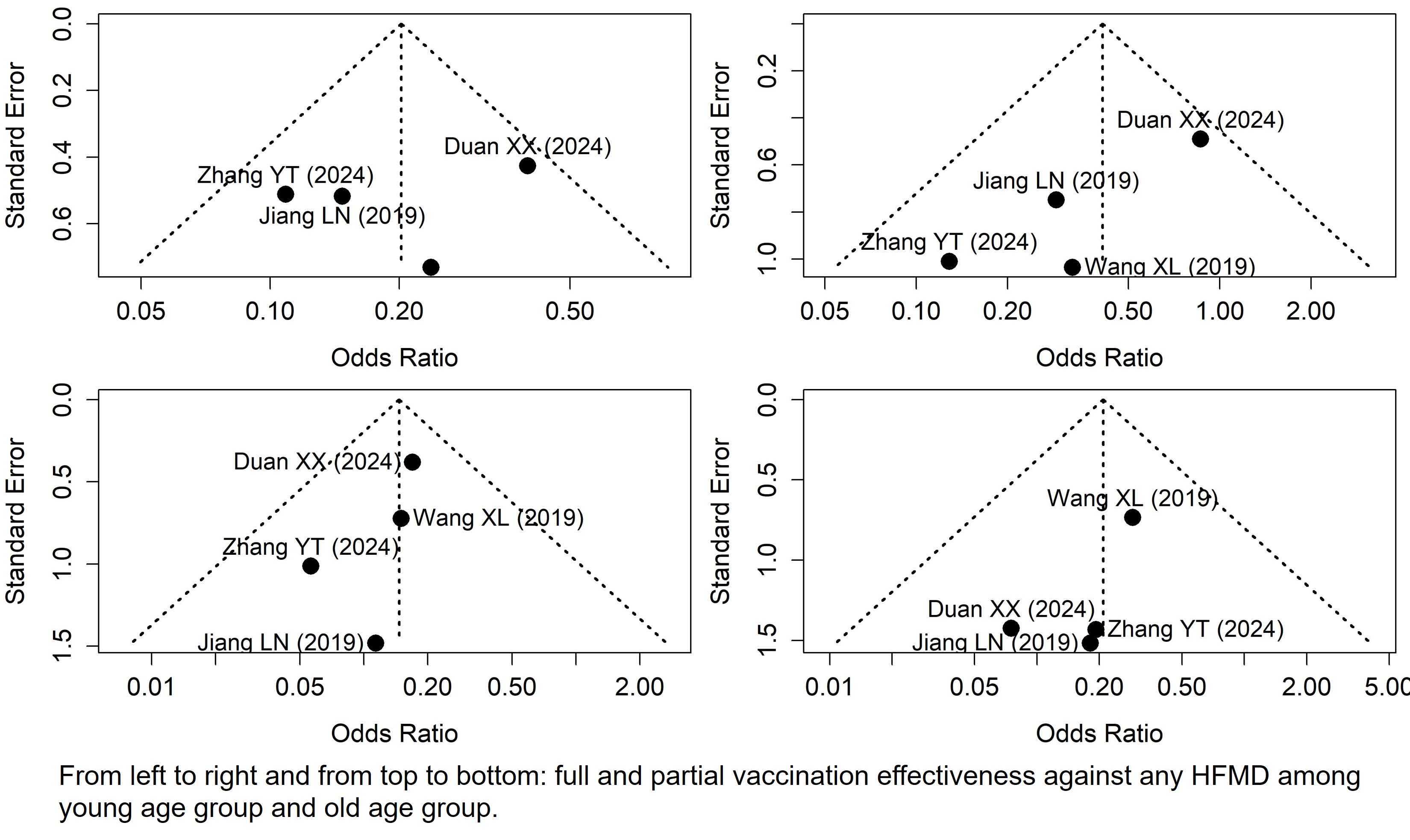

Supplement: S10 Fig — (TIF) [file pone.0323782.s016.tif]
